# Antibacterial and Antitubercular Activities of Cinnamylideneacetophenones

**DOI:** 10.3390/molecules22101685

**Published:** 2017-10-10

**Authors:** Carlos R. Polaquini, Guilherme S. Torrezan, Vanessa R. Santos, Ana C. Nazaré, Débora L. Campos, Laíza A. Almeida, Isabel C. Silva, Henrique Ferreira, Fernando R. Pavan, Cristiane Duque, Luis O. Regasini

**Affiliations:** 1 Laboratory of Green and Medicinal Chemistry, Department of Chemistry and Environmental Sciences, Institute of Biosciences, Humanities and Exact Sciences, São Paulo State University (Unesp), São José do Rio Preto, SP 15054-000, Brazil; carlos_polaquini@hotmail.com (C.R.P.); guilherme_panz@hotmail.com (G.S.T.); carolnazare@uol.com.br (A.C.N.); laiza_araujo1@hotmail.com (L.A.A.); 2 Department of Pediatric Dentistry and Public Health, School of Dentistry, São Paulo State University (Unesp), Araçatuba, SP 16015-050, Brazil; vanessarodrigues_22@hotmail.com (V.R.S.); cristianeduque@yahoo.com.br (C.D.); 3 Department of Biological Sciences, School of Pharmaceutical Sciences, São Paulo State University (Unesp), Araraquara, SP 14800-903, Brazil; leite.debora26@gmail.com (D.L.C.); isabelcrs10@gmail.com (I.C.S.); fernandopavan@fcfar.unesp.br (F.R.P.); 4 Department of Biochemistry and Microbiology, Institute of Biosciences, São Paulo State University (Unesp), Rio Claro, SP 13506-900, Brazil; henfer@rc.unesp.br

**Keywords:** cinnamaldehyde, cinnamylideneacetophenone, antimicrobial, antibacterial, antitubercular, *Mycobacterium*, Claisen-Schmidt reaction

## Abstract

Cinnamaldehyde is a natural product with broad spectrum of antibacterial activity. In this work, it was used as a template for design and synthesis of a series of 17 cinnamylideneacetophenones. Phenolic compounds **3** and **4** exhibited MIC (minimum inhibitory concentration) and MBC (minimum bactericidal concentration) values of 77.9 to 312 µM against *Staphylococcus aureus*, *Streptococcus mutans*, and *Streptococcus sanguinis.* Compounds **2**, **7**, **10**, and **18** presented potent effects against *Mycobacterium tuberculosis* (57.2 µM ≤ MIC ≤ 70.9 µM). Hydrophilic effects caused by substituents on ring B increased antibacterial activity against Gram-positive species. Thus, log *P*_o/w_ were calculated by using high-performance liquid chromatography-photodiode array detection (HPLC-PDA) analyses, and cinnamylideneacetophenones presented values ranging from 2.5 to 4.1. In addition, the effects of **3** and **4** were evaluated on pulmonary cells, indicating their moderate toxicity (46.3 µM ≤ IC_50_ ≤ 96.7 µM) when compared with doxorubicin. Bioactive compounds were subjected to in silico prediction of pharmacokinetic properties, and did not violate Lipinski’s and Veber’s rules, corroborating their potential bioavailability by an oral route.

## 1. Introduction

Cinnamaldehyde (**1**) is the main component in cinnamon bark oil (50–70%), and it is responsible for its spicy and sweet taste [1,2]. Structurally, cinnamaldehyde is a natural simple phenylpropanoid, bearing an α,β-unsaturated aromatic aldehyde. The synthetic applications of **1** are vast and related to its abundance and low economic costs, and have led to a collection of compounds with high chemodiversity [3].

The spectrum of antibacterial activity of **1** has been extensively reported and includes effects against Gram-positive, Gram-negative, and mycobacteria species, such as *Escherichia coli* [4], *Helicobacter pylori* [5], *Pseudomonas aeruginosa* [6], *Salmonella enterica* [7], *Staphylococcus aureus* [8], *Mycobacterium avium* [9], *Mycobacterium bovis*, and *Mycobacterium tuberculosis* [10]. Moreover, **1** demonstrated selectivity for pathogenic species in comparison to commensal intestinal microbiota [11,12]. The mode of action of **1** involves bacterial multi-targets, including membrane disruption [13], cell division inhibition [14], and reactive-oxygen species induction [15].

**Scheme 1:**
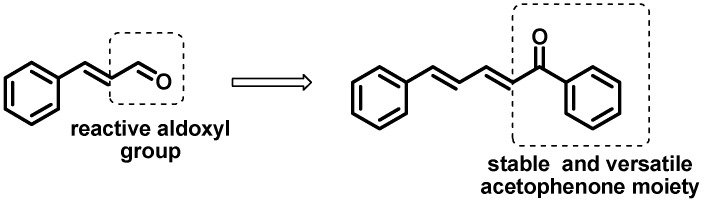
Design of cinnamylideneacetophenones.

In vitro and in vivo toxicity of **1** have been clearly elucidated due to its application as a food additive [16,17]. In this context, **1** was classified as GRAS (generally recognized as safe) by the FDA (Food and Drug Administration) [18]. On the other hand, cinnamaldehyde possess an aliphatic aldoxyl group, which has undesirable functionality due to its high reactivity [19]. Thus, we designed a series of cinnamylideneacetophenones with the replacement of the aldoxyl by an acetophenone moiety. The resonance of an additional benzene ring leads to compounds that are chemically more stable than cinnamaldehyde, allowing insertion of versatile substituents to derive preliminary structure-activity relationships (Scheme 1).

In our ongoing search for new antibacterials from natural product-based synthetic compounds, we synthesized a series of 17 cinnamylideneacetophenones (**2**–**18**). Their antibacterial and antitubercular activities were evaluated against Gram-positive and Gram-negative species, as well as *M. tuberculosis*. The two most active compounds (**3** and **4**) were investigated regarding their selective toxicity on eukaryotic cells by using two human pulmonary cell lineages (MRC-5 and A549). In addition, hydrophilicity of all compounds was measured by HPLC-PDA experiments, which enabled calculation of partition coefficients (log *P*_o/w_). In silico investigations using the Molinspiration online property calculation toolkit contributed to the prediction of absorption, distribution, metabolism and excretion (ADME) properties of selected bioactive cinnamylideneacetophenones.

## 2. Results and Discussion

### 2.1. Chemistry

**Scheme 2:**
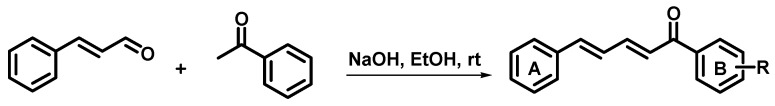
Synthesis of cinnamylideneacetophenones.

Synthesis of cinnamylideneacetophenones **2**–**18** was achieved by Claisen-Schmidt condensation, using protocol reported by Weldon and co-authors, with minor modifications [20,21,22] (Scheme 2). 

Reactions between cinnamaldehyde and corresponding acetophenone were performed under basic catalysis, at room temperature, resulting in yields of 33–98% (Table 1). Cinnamylideneacetophenones exhibited substitutions at *para*- and *meta*-positions on ring B by electron-donating groups (OH, NH_2_, OMe, OCH_2_O, and Me) and electron-withdrawing groups (F, Cl, Br, NO_2_, and CF_3_). In general, a clear and unambiguous relationship between the stereoelectronic effect of substituents on ring B and reaction yields cannot be established.

The structure of compounds was identified by their melting point, UV-Vis, ^1^H-NMR, and ^13^C-NMR spectra analyses and was compared with previous data [20,21,22,23]. Out of 17 compounds, compound **9** was identified as a new chemical entity. UV-Vis spectra performed on HPLC-PDA experiments indicated λ_max_ values ranging from 343 to 361 nm, corroborating the presence of an α,β,γ,δ-unsaturated ketone system in cinnamylideneacetophenone structures. For all compounds, NMR data, including chemical shifts, integrations, multiplicities, and coupling constants, corresponded with the proposed structures. Cinnamylideneacetophenones were analyzed by HPLC-PDA and their purity was measured through peak area values, which ranged from 96.1 to 99.8%. Spectra and chromatograms were presented as supplementary material.

### 2.2. Antibacterial, Antitubercular Activity, and Structure-Activity Relationship (SAR)

For antibacterial evaluations, three Gram-positive and two Gram-negative species were used, including *Staphylococcus aureus* (ATCC 14458), *Streptococcus mutans* (ATCC 25175), *Streptococcus sanguinis* (ATCC 10557), *Pseudomonas aeruginosa* (ATCC 15442) and *Escherichia coli* (ATCC 10536). The antibacterial potency was demonstrated by MIC (minimum inhibitory concentration) and MBC (minimum bactericidal concentration) values. MIC and MBC were determined by broth microdilution method, in 96-well microtiter plates, following the criteria previously described by the National Committee for Clinical Laboratory Standards [24]. Penicillin, vancomycin, and chlorhexidine were used as positive controls. All cinnamylideneacetophenones were assayed in concentrations ranging from 3.9 to 500 µM.

For antitubercular assays, *Mycobacterium tuberculosis* H37Rv (ATCC 27294) was used as a reference strain. Antitubercular potency was expressed as MIC values, which were established by protocol reported by Palomino and co-author, with minor modifications [25,26]. Isoniazid was used as the reference antitubercular drug. Compounds **1**–**18** were evaluated in concentrations ranging from 1 to 200 µM (Table 2).

Cinnamaldehyde (**1**) displayed MIC > 500 µM against *S. aureus*, *S. mutans*, *S. sanguinis*, *P. aeuruginosa*, and *E. coli*. These results corroborated other investigations, which have described MIC values higher than 500 µM. Domadia and collaborators reported MIC values of 1000 mg/L (7566 µM) against *E. coli* [14]. Ferro and co-authors described MIC values of 0.25 mg/mL (1891 µM) against the *S. aureus* strains [27]. On the other hand, our result against *M. tuberculosis* (MIC > 200 µM) was opposite to that described by Ochoa and colleagues, who reported MIC value 3.12 µg/mL (23 µM) [10].

We used unsubstituted cinnamylideneacetophenone (**2**) as a framework to investigate the relationship between substitutions on ring B and antibacterial/antitubercular activities. Firstly, **2** was compared with phenolic compounds **3** and **4**, which suggested the hydroxyl group improved antibacterial activity, mainly against Gram-positive species. Compound **3** demonstrated MIC and MBC values of 77.9 and 156 µM against *S. aureus*, *S. mutans* and *S. sanguinis*, respectively. Compound **4** displayed similar antibacterial potency with MIC and MBC values ranging from 77.9 to 312 µM. On the other hand, the hydroxyl group reduced antitubercular activity, because unsubstituted compound **2** (MIC = 57.2 µM) was more potent than phenolics **3** (MIC = 88.8 µM) and **4** (MIC = 81.9 µM). Also, aminoderivative **5** exhibited activity against *S. mutans* (MIC = MBC = 313 µM) and was not able to act against *S. aureus*, *S. sanguinis,* nor *M. tuberculosis*. These results indicate that electron-donating and hydrophilic groups interfere with bioactivity, and weak acidic compounds **3** and **4** were more active than basic compound **5**.

Second, comparisons were made to confirm the importance of hydrophilic, acidic, and electron-donating groups for bioactivity. Methoxylated compounds **6** and **7** were less active than their hydroxylated congeners **3** and **4** against *S. aureus, S. mutans,* and *S. sanguinis*, with MIC values ≥ 296 µM. 3,4-Methylenedioxylated compound **8**, which is closely similar to **6** and **7**, was inactive with MIC values > 500 µM. Vanillyl compound **9**, which possess *meta*-methoxyl and *para*-hydroxyl groups, was active effect against *S. aureus* (MIC = MBC = 279 µM).

Third, comparisons were made to establish if hydrophilicity or electron donation is pivotal for bioactivity. Cinnamylideneacetophenones carrying hydrophobic and electron-withdrawing substituents (F, Cl, Br, CF_3_, and NO_2_) (**10**–**16**) were inactive against *S. aureus*, *S. mutans,* and *S. sanguinis*. In addition, methylated compounds **17** and **18**, which are compounds substituted by hydrophobic and electron-donating groups, were also inactive. Thus, the electronic effect was not crucial for antibacterial activity, but hydrophilicity increased it. In contrast, antitubercular assays indicated *para*-fluorinated and *para*-methylated compounds **10** (MIC = 67.4 µM) and **18** (MIC = 70.9 µM), respectively, were active against *M. tuberculosis*. This comparison suggested hydrophobic groups reduced the antibacterial activity and the electronic nature of the substituent was irrelevant for antitubercular activity.

Fourth, we evaluated the effect of the position of the substituent on bioactivity. For pairs of regioisomers **6**/**7** and **17**/**18**, the position of the methoxyl and methyl groups, respectively, were essential for antitubercular activity. *Para*-substituted compounds **7** and **18** presented MIC values of 66.6 and 70.9 µM, respectively, against *M. tuberculosis*, while their respective *meta*-substituted compounds were inactive (MIC > 200 µM). The position of hydroxyl and nitro substituents of regioisomer pairs **3**/**4** and **15**/**16** did not interfere with antitubercular activity.

Interestingly, cinnamylideneacetophenones that were bioactive against Gram-positive species were not able to inhibit Gram-negative species growth, such as *P. aeruginosa* and *E. coli*, therefore, they exhibited reduced spectrum. Gram negative bacteria demonstrate a complex barrier system, which makes them less susceptible to action of antibacterial agents. As part of this system, the cell wall is mainly responsible for impermeability due to the presence of an external envelope, composed of lipopolysaccharides, phospholipids, and proteins [28,29].

### 2.3. Determination of Partition Coefficient Log P_o/w_

Preliminary structure-activity relationship (SAR) indicated the essentiality of the hydrophilicity for antibacterial and antitubercular activities. Thus, we calculated the partition coefficient (*n*-octanol/water) (log *P*_o/w_) by using the HPLC-PDA method suggested by Organisation for Economic Co-operation and Development (OECD) protocols [30]. Log *P*_o/w_ values of the cinnamylideneacetophenones **2**–**18** are summarized in Table 2. The reference compounds, aniline, phenol, acetophenone, benzoic acid, benzene, toluene, benzophenone, and thymol, were used to construct a log *K* versus log *P*_o/w_ curve. The capacity factor (log *K*) of the cinnamylideneacetophenones was determined from retention times. Anti-*Staphylococcus* and anti-*Streptococcus* compounds exhibited log *P*_o/w_ values ranging from 2.5 to 3.3. Among these, compounds **3** and **4** were the most potent, with log *P*_o/w_ values of 2.7 and 2.6, respectively. The four most active anti-*Mycobacterium* compounds (MIC ≤ 70.9 µM) presented log *P*_o/w_ values ranging from 3.2 to 3.5. Highly hydrophobic compounds displayed log *P*_o/w_ > 3.5 (**11**–**14** and **17**) and were not able to act against *S. aureus*, *S. mutans*, *S. sanguinis*, and *M. tuberculosis*.

### 2.4. Evaluation of Cytotoxicity and Determination of Selectivity Index

The two most potent compounds (**3** and **4**) were investigated for their selective toxicity against eukaryotic cells. Human lung fibroblast (MRC-5, ATCC, CCL-171) and human lung adenocarcinoma cells (A549, ATCC, CCL-185) were used for cytotoxicity evaluations. The choice for pulmonary cells was based on their importance as host site infection of *S. aureus* and *M. tuberculosis* [26,31]. The cytotoxic assays were performed using protocol described by our group [32]. Compounds **3** and **4** displayed similar effects against both human cells, demonstrating values of IC_50_ (concentration able to inhibit 50% of cell growth) ranging from 46.3 to 96.7 µM (Table 3). These compounds were moderately toxic against pulmonary cells when compared with doxorubicin which exhibited IC_50_ values of 0.7 and 1.8 µM against MRC-5 and A549, respectively. In order to compare the effect towards prokaryotic cells versus eukaryotic cells, the selectivity indexes (SI) of **3** and **4** were calculated as the ratio of cytotoxicity to antimicrobial activities (SI = IC_50_ eukaryotic cell/MIC prokaryotic cells) (Table 3). The effectiveness of a compound is indicated by SI ≥ 10. However, SI ≤ 1 indicates toxicity of that compound for eukaryotic cells and low selectivity for the prokaryotic cells. Despite **3** and **4** demonstrating promising antibacterial and antitubercular activities, these compounds showed SI values ranging from 0.5 to 1.2.

Antimicrobial agents with in vitro toxicity against human cells should not be ignored. Although SI values are relevant as a general orientation, this ratio cannot be used as the unique parameter to decide if a compound can continue to preclinical trials [33,34]. In this context, in vivo toxicity trials are highly indicated to capture the potential of **3** and **4** as valuable molecules.

**Figure 1:**
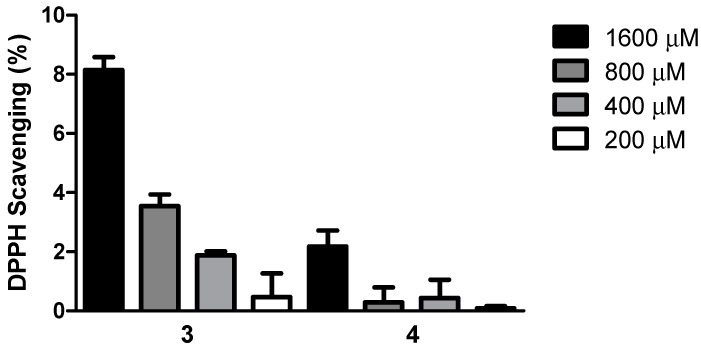
Radical scavenging ability of selected cinnamylideneacetophenones. DPPH = 2,2-diphenyl-1-picrylhydrazyl.

The low selectivity of the cinnamylideneacetophenones **3** and **4** can be correlated with their phenolic nature. Studies report the ability of phenolic compounds to act on widespread radical processes shared by eukaryotic and prokaryotic cells [35]. We hypothesized this behavior may be responsible by simultaneous activity toward bacteria and pulmonary cells. Therefore, we evaluated the radical scavenging ability of **3** and **4** using DPPH (2,2-diphenyl-1-picrylhydrazyl) protocol [36,37]. Compounds **3** and **4** were evaluated at 1600, 800, 400, and 200 µM and exhibited all percentages of DPPH scavenging ability below 8% (Figure 1), suggesting their low efficacy on radical processes. Altogether, low selectivity of **3** and **4** cannot be strongly related to radical processes. Quercetin and gallic acid were used as positive controls due to their phenolic nature and potent radical scavenging ability [38,39]. These compounds were tested in the same concentrations used for compounds **3** and **4**, demonstrating percentages of DPPH scavenging ability ≥95%.

### 2.5. In Silico Prediction of ADME of Cinnamylideneacetophenones

Several reasons contribute to the attrition of drug candidates, including pharmacokinetic difficulties, insufficient efficacy, and adverse reactions in animal and humans. In the development phase of oral drugs, nearly 30% of drug candidates fail due to poor ADME properties [40]. Bioactive cinnamylideneacetophenones were also subjected to pharmacokinetic filters. An in silico study was performed for prediction of ADME properties using the Molinspiration property explorer toolkits [41]. In Table 4, we summarize the computed molecular properties of the selected compounds, such as absorption (%ABS), topological polar surface area (TPSA), lipophilicity (*mi*log *P*), molecular weight (MW), number of hydrogen bond donors (HBD), number of hydrogen bond acceptors (HBA), violations of Lipinski’s rule of five (Ro5), and number of rotatable bonds (NROTB).

TPSA has been used as descriptor for characterizing absorption and passive transportation properties through biological membranes, allowing a good prediction of transport of candidate drugs in the intestines and through the blood-brain barrier (BBB). TPSA is sum of van der Waals surface areas of electronegative atoms (oxygen and nitrogen). Erlt and co-authors described TPSA values useful for prediction of drug transport properties [42]. Compounds with TPSA values in the range ≤150 Å^2^ have good intestinal absorption and ≤90 Å have BBB penetration. All selected compounds exhibited TPSA values ≤46.53 Å^2^. The four more potent antitubercular cinnamylideneacetophenones (**2**, **7**, **10**, and **18**) displayed TPSA values ranging from 17.07 to 26.30 Å^2^, suggesting their efficient BBB penetration, which is a crucial property to combat tuberculous meningitis [43]. TPSA was used to calculate the percentage of absorption (%ABS) according to the equation: %ABS = 109 − 0.345 × TPSA. All selected compounds demonstrated high %ABS, ranging from 92.9 to 100. The three most potent anti-*Mycobacterium* compounds (**2**, **10**, and **18**) exhibited a %ABS value of 100, which is relevant for their oral absorption, as well as for penetration through the highly hydrophobic mycolic acid layer of *M. tuberculosis* [44].

In order to achieve the optimum properties intended for oral administration, Lipinski and collaborators established a series of rules for drug candidates such as: (a) a log *P* less than 5, (b) a molecular weight less than 500 Da, (c) less than five hydrogen bond donors (sum of OH and NH groups), and (d) less than 10 hydrogen bond acceptors (sum of oxygen and nitrogen) [45]. Selected cinnamylideneacetophenones did not violate Lipinski’s parameters, corroborating their oral drug likeness properties.

Among pharmacokinetic difficulties to drug candidates, low oral bioavailability has been considered a central reason for interrupting further drug development phases. In this context, Veber and collaborators suggested a filter for drug candidates, containing two essential criteria for oral bioavailability in rats: (a) TPSA equal or less than 140 Å^2^ and (b) ten or fewer rotatable bonds [46]. All selected compounds exhibited NROTB ≤5 and TPSA values ≤46.53 Å^2^, making them potentially bioavailable by an oral route.

## 3. Materials and Methods

### 3.1. Synthesis of Cinnamylideneacetophenones

All starting reagents were purchased from Sigma-Aldrich. Compounds were synthesized by Claisen-Schmidt condensation, according to the method reported by Weldon and co-authors, with minor modifications [20,21,22]. Cinnamaldehyde (6 mmol) was added to solution of respective acetophenone (5 mmol) in ethanol (5 mL). The solution was stirred at room temperature for 30 min, followed by dropwise addition of 5 mL ethanolic solution of NaOH (1.0 M). The progress of reaction was checked by thin-layer chromatography (TLC) analyses. Reaction medium was poured into crushed ice. Precipitated crude products were filtered, washed with cold water, and dried at room temperature. Soluble crude products were partitioned with ethyl acetate (3 × 25 mL). Organic phase was concentrated under reduced pressure. Compounds **2**, **7**, **8** and **10**–**7** were recrystallized in ethanol. Compounds **3**–**6**, **9** and **18** were recrystallized in mixtures of hexane and chloroform.

### 3.2. Antibacterial Activity of Cinnamylideneacetophenones

Microorganisms used in this study were *Staphylococcus aureus* (ATCC 14458), *Streptococcus mutans* (ATCC 25175), *Streptococcus sanguinis* (ATCC 10557), *Pseudomonas aeruginosa* (ATCC 15442), and *Escherichia coli* (ATCC 10536). MIC and MBC values were determined by broth microdilution method, in 96-well microtiter plates, following the criteria previously described by the National Committee for Clinical Laboratory Standards [24]. Bacterial cell cultures in Brain Heart Infusion broth (BHI) at the mid-log phase were harvested by centrifugation for 10 min at 3000× *g*. Supernatant was discarded and the pellet resuspended in Mueller-Hinton broth (Difco Laboratories, Detroit, MI, USA). The final bacterial suspension was 5–10 × 10^5^ CFU (colony forming units) per mL. Compounds were diluted in sterile deionized water in concentrations ranging from 500 to 3.9 µM. Bacterial suspension was inoculated in each well. The plates were incubated at 37 °C for 24 h in 5% CO_2_. Thus, 15 µL of 0.01% resazurin staining was added to each well and incubated for 4 h to determine cell viability. Wells corresponding to MIC and at least three previous wells were homogenized, serially diluted, and plated on Mueller-Hinton agar to determine MBC. The plates were incubated at 37 °C for 24 h in 5% CO_2_. Number of colonies forming units per mL (CFU/mL) was determined. The MBC was considered when the compounds killed more than 99% of the tested bacterial culture. Penicillin G, vancomycin, chlorhexidine, and culture medium were used as positive and negative controls, respectively. Three independent assays were performed.

### 3.3. Antitubercular Activity of Cinnamylideneacetophenones

The antitubercular activity of compounds was determined by the REMA (Resazurin Microtiter Assay) method [25,26]. Stock solutions of compounds were prepared in DMSO (dimethyl sulfoxide) and diluted in Middlebrook 7H9 broth (Difco) supplemented with oleic acid, albumin, dextrose, and catalase (OADC), to obtain the final concentration range from 100 to 1 µM. Suspension of *Mycobacterium tuberculosis* H37Rv strain (ATCC 27294) was cultured in Middlebrook 7H9 broth supplemented with OADC. When the culture obtained a turbidity of McFarland standard No. 1, it was adjusted by 3–5 × 10^5^ CFU per mL. Thus, 100 µL of inoculum were added to each well in a 96-well microplate with 100 µL of the compound solution. Plates were incubated for 7 days at 37 °C and 5% CO_2_. Resazurin was added (30 µL of 0.01%). The fluorescence of the wells was measured after 24 h on Cytation 3 (BioTek, Winooski, VT, USA) equipment. MIC value was defined as the lowest concentration resulting in 90% inhibition of growth of *M. tuberculosis*. Isoniazid and culture medium were used as positive and negative controls, respectively. Three independent assays were performed.

### 3.4. Partition Coefficient (log P_o/w_) of Cinnamylideneacetophenones

Values of log *P*_o/w_ were determined using the HPLC method according to OECD-Guidelines for the Testing of Chemicals [30], on Shimadzu (Kyoto, Japan) HPLC-PDA (model CBM 20-A) equipped with a UV-Vis detector (model SPD-20A), binary pumping system (model LC-20AD), solvent degasser (model DGU-20A_3r_), and a Phenomenex Gemini C-18 column (Torrance, CA, USA) (250 mm × 4.6 mm; 5 µm; 110 Å). Analysis was performed on isocratic mode in MeOH:H_2_O (3:1) and 1.0 mL/min. Injected volume of sample was 20.0 µL and the wavelength in the detector was adjusted according to λ_max_ of each compound. Reference compounds phenol, acetophenone, benzoic acid, toluene, and thymol were used to construct the curve log *K* × log *P*_o/w_. Capacity factor of the cinnamylideneacetophenones was determined from their retention times and interpolated in linearity curve log *K* × log *P*_o/w_. Experiments were performed in duplicate.

### 3.5. Toxic Effect on Lung Cells of Selected Cinnamylideneacetophenones

Cytotoxicity of compounds was evaluated according to Silva and colleagues [32]. The cell lineages used were MRC-5 (human lung fibroblast cell line—ATCC, CCL-171) and A549 (human lung adenocarcinoma epithelial cell line—ATCC, CCL-185). Cells were routinely maintained in complete medium supplemented with 10% fetal bovine serum (FBS) plus amphotericin B (2 mg/L) and gentamicin (50 mg/L) at 37 °C in a humidified 5% CO_2_ atmosphere. For cytotoxicity assay, 2.5 × 10^4^ cells/mL were seeded into a 96-well cell culture plate with a total volume of 100 µL for 24 h. Cells were treated with compounds at concentrations ranging from 100 to 1 µM. After 24 h incubation, medium was removed, and 50 µL of resazurin (0.01% *w*/*v*) was added to each well, and the plates were incubated at 37 °C for 3 h. Fluorescence of wells was measured on a Synergy H1 microplate reader (BioTek, Winooski, VT, USA) in which were used excitation (530 nm) and emission (590 nm) filters. The IC_50_ values were calculated from a calibration curve by regression analysis. Doxorubicin and untreated cells constituted the positive and negative controls, respectively. Three independents assays were performed.

### 3.6. DPPH Scavenging Ability of Selected Cinnamylideneacetophenones

Radical scavenging activity of compounds **3** and **4** was determined using 2,2-diphenyl-1-picrylhydrazyl (DPPH) [36,37]. Aliquots of 10 µL of **3**, **4**, and positive controls (quercetin and gallic acid) at concentrations 1600, 800, 400, and 200 μM (in EtOH) were individually added in 190 µL DPPH (100 μM). The mixtures were incubated in the dark at 25 °C for 30 min. Remaining DPPH was determined spectroscopically at 490 nm by blanking against absolute ethanol. Radical scavenging ability was expressed by using mean values obtained from triplicates as the percentage of radicals reduced by calculating the following equation: *DPPH scavenging* % = [1 − (*A_sample_*/*A*) × 100], where *A* is test absorbance without sample (only EtOH and DPPH) and *Asample* is test absorbance with compounds.

## 4. Conclusions

In summary, a series of cinnamylideneacetophenones containing several substituents on ring B were designed and synthesized as part of our ongoing search for antibacterial and antitubercular compounds based on natural products. Five compounds (**3**–**6** and **9**) exhibited potent antibacterial activity against Gram-positive species. Seven compounds (**2**–**4**, **7**, **9**, **10** and **18**) were able to inhibit *M. tuberculosis* growth. Preliminary SAR investigations suggested hydrophilicity is the central parameter for antibacterial and antitubercular activities. Furthermore, our results corroborated the potential of cinnamaldehyde as a privileged starting material and template for synthetic collection of hits; it did not violate Lipinski’s and Veber’s boundaries.

## Figures and Tables

**Table 1:** Yields of cinnamylideneacetophenones.

Entry	Name	R	Yield (%)
**2**	(2*E*,4*E*)-1,5-diphenylpenta-2,4-dien-1-one	H	33
**3**	(2*E*,4*E*)-1-(3-hydroxyphenyl)-5-phenylpenta-2,4-dien-1-one	*m*-OH	91
**4**	(2*E*,4*E*)-1-(4-hydroxyphenyl)-5-phenylpenta-2,4-dien-1-one	*p*-OH	82
**5**	(2*E*,4*E*)-1-(3-aminophenyl)-5-phenylpenta-2,4-dien-1-one	*m*-NH_2_	35
**6**	(2*E*,4*E*)-1-(3-methoxyphenyl)-5-phenylpenta-2,4-dien-1-one	*m*-OMe	78
**7**	(2*E*,4*E*)-1-(4-methoxyphenyl)-5-phenylpenta-2,4-dien-1-one	*p*-OMe	35
**8**	(2*E*,4*E*)-1-(3,4-methylenedioxy)-5-phenylpenta-2,4-dien-1-one	-OCH_2_O-	83
**9**	(2*E*,4*E*)-1-(4-hydroxy-3-methoxyphenyl)-5-phenylpenta-2,4-dien-1-one	*m*-OMe,*p*-OH	98
**10**	(2*E*,4*E*)-1-(4-fluorophenyl)-5-phenylpenta-2,4-dien-1-one	*p*-F	71
**11**	(2*E*,4*E*)-1-(4-chlorophenyl)-5-phenylpenta-2,4-dien-1-one	*p*-Cl	72
**12**	(2*E*,4*E*)-1-(4-bromophenyl)-5-phenylpenta-2,4-dien-1-one	*p*-Br	86
**13**	(2*E*,4*E*)-1-(3,4-dichlorophenyl)-5-phenylpenta-2,4-dien-1-one	*m*,*p*-diCl	79
**14**	(2*E*,4*E*)-1-(3-(trifluoromethyl)phenyl)-5-phenylpenta-2,4-dien-1-one	*m*-CF_3_	88
**15**	(2*E*,4*E*)-1-(3-nitrophenyl)-5-phenylpenta-2,4-dien-1-one	*m*-NO_2_	85
**16**	(2*E*,4*E*)-1-(4-nitrophenyl)-5-phenylpenta-2,4-dien-1-one	*p*-NO_2_	84
**17**	(2*E*,4*E*)-1-(3-methylphenyl)-5-phenylpenta-2,4-dien-1-one	*m*-Me	49
**18**	(2*E*,4*E*)-1-(4-methylphenyl)-5-phenylpenta-2,4-dien-1-one	*p*-Me	53

**Table 2:** Antibacterial and antitubercular activities and log *P*_o/w_ of cinnamylideneacetophenones.

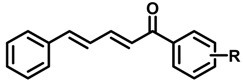											
Entry	R	Sa *		Sm *		Ss *		Pa *	Ec *	Mt *	log *P*_o/w_
		MIC	MBC	MIC	MBC	MIC	MBC	MIC	MIC	MIC	
**1**	-	>500	-	>500	-	>500	-	>500	>500	>200	-
**2**	H	>500	-	>500	-	>500	-	>500	>500	57.2	3.2
**3**	*m*-OH	77.9	156	77.9	156	77.9	156	>500	>500	88.8	2.7
**4**	*p*-OH	77.9	77.9	156	312	156	312	>500	>500	81.9	2.6
**5**	*m*-NH_2_	>500	-	313	313	>500	-	>500	>500	>200	2.5
**6**	*m*-OMe	296	296	>500	-	>500	-	>500	>500	>200	3.3
**7**	*p*-OMe	>500	-	>500	-	>500	-	>500	>500	66.6	3.2
**8**	-OCH_2_O-	>500	-	>500	-	>500	-	>500	>500	>200	3.2
**9**	*m*-OMe,*p*-OH	279	279	>500	-	>500	-	>500	>500	83.5	2.7
**10**	*p*-F	>500	-	>500	-	>500	-	>500	>500	67.4	3.3
**11**	*p*-Cl	>500	-	>500	-	>500	-	>500	>500	>200	3.7
**12**	*p*-Br	>500	-	>500	-	>500	-	>500	>500	>200	3.8
**13**	*m*,*p*-diCl	>500	-	>500	-	>500	-	>500	>500	>200	4.1
**14**	*m*-CF_3_	>500	-	>500	-	>500	-	>500	>500	>200	3.7
**15**	*m*-NO_2_	>500	-	>500	-	>500	-	>500	>500	>200	3.3
**16**	*p*-NO_2_	>500	-	>500	-	>500	-	>500	>500	>200	3.2
**17**	*m*-Me	>500	-	>500	-	>500	-	>500	>500	>200	3.6
**18**	*p*-Me	>500	-	>500	-	>500	-	>500	>500	70.9	3.5
**PEN**	-	-	-	-	-	0.4	0.4	-	118	-	-
**VAN**	-	3.3	6.8	-	-	3.3	3.3	-	53.8	-	-
**CHX**	-	-	-	2.4	4.7	4.7	9.5	19.4	-	-	-
**ISO**	-	-	-	-	-	-	-	-	-	0.3	-

* MIC = minimum inhibitory concentration; MBC = minimum bactericidal concentration. MIC and MBC in µM. Sa = *Staphylococcus aureus*; Sm = *Streptococcus mutans*; Ss = *Streptococcus sanguinis*; Pa = *Pseudomonas aeruginosa*; Ec = *Escherichia coli*; Mt = *Mycobacterium tuberculosis*; PEN = penicillin; VAN = vancomycin; CHX = chlorhexidine; ISO = isoniazid; - not determined.

**Table 3:** Toxicity against human lung cells and selectivity index of selected cinnamylideneacetophenones.

Entry	MRC-5	SI		A549	SI	
	IC_50_ *	*Sa*	*Mt*	IC_50_ *	*Sa*	*Mt*
**3**	46.3	0.6	0.5	96.7	1.2	1.2
**4**	53.1	0.7	0.6	77.5	1.0	0.9
**doxorubicin**	0.7	-	-	1.8	-	-

* IC_50_ in µM.

**Table 4:** Lipinski’s and Veber’s parameters of selected cinnamylideneacetophenones.

Entry	%ABS	TPSA (Å^2^)	*mi*log *P*	MW	HBD	HBA	Lipinski’s Violations	NROBT	Veber’s Violations
**Ro5**	-	-	≤5.00	<500	≤5	≤10	1	-	-
**Vb**	-	≤140	-	-	-	-	-	≤10	0
**1**	100	17.07	2.48	132.16	1	0	0	2	0
**2**	100	17.07	4.33	234.30	1	0	0	4	0
**3**	96.1	37.30	3.83	250.30	2	1	0	4	0
**4**	96.1	37.30	3.85	250.30	2	1	0	4	0
**5**	94.1	43.09	3.38	249.31	2	2	0	4	0
**6**	99.9	26.30	4.36	264.32	2	0	0	5	0
**7**	99.9	26.30	4.39	263.32	2	0	0	5	0
**9**	92.9	46.53	3.67	280.32	3	1	0	5	0
**10**	100	17.07	4.49	252.29	1	0	0	4	0
**18**	100	17.07	4.78	248.32	1	0	0	4	0

%ABS = percent absorption; TPSA = topological polar surface area; *mi*log *P* = lipophilicity; MW = molecular weight; HBD = number of hydrogen bond donors; HBA = number of hydrogen bond acceptors; NROBT = number of rotatable bonds; Ro5 = Boundaries of Lipinski’s rules; Vb = Boundaries of Veber’s rules.

## Supplementary Materials

Supplementary materials are available online.
