# Supplementary material for: Antibacterial and Antitubercular Activities of Cinnamylideneacetophenones
Source: Molecules. 2017 Oct 10;22(10):1685. doi: 10.3390/molecules22101685 (PMC6151560; doi:10.3390/molecules22101685)

Supplementary material

Antibacterial and Antitubercular Activities of Cinnamylideneacetophenones

Carlos R. Polaquini[a,†], Guilherme S. Torrezan[a,†], Vanessa R. Santos[b], Ana C. Nazaré[a], Débora L. Campos[c], Laíza A. Almeida[a], Isabel C. Silva[c], Henrique Ferreira[d], Fernando R. Pavan[c], Cristiane Duque[b] and Luis O. Regasini[a,]*

[a]Laboratory of Green and Medicinal Chemistry, Department of Chemistry and Environmental Sciences, Institute of Biosciences, Humanities and Exact Sciences, São Paulo State University (Unesp), São José do Rio Preto, SP, Brazil

[b]Department of Pediatric Dentistry and Public Health, Araçatuba Dental School, São Paulo State University (Unesp), Araçatuba, SP, Brazil

[c]Department of Biological Sciences, School of Pharmaceutical Sciences, São Paulo State University (Unesp), Araraquara, SP, Brazil

[d]Department of Biochemistry and Microbiology, Institute of Biosciences, São Paulo State University (Unesp), Rio Claro, SP, Brazil

* Corresponding author. Tel.: +55 17 3221-2362.

E-mail address: regasini@ibilce.unesp.br (L.O. Regasini).

[†] These two authors contributed equally to this work.

1. SPECTROSCOPY DATA ANALYSES

Melting points of the cinnamylideneacetophenones were determined on a capillary point apparatus with a digital thermometer. UV-Vis spectrum and purity of compounds were recorded on HPLC-PAD analyses performed for calculations of partition coeficiente, using MeOH:H_2O (3:1) as mobile phase. ^{1}H NMR and ^{13}C NMR spectra were recorded on a Bruker Avance III 14.1 T (600 MHz), Bruker Avance III 9.4 T (400 MHz) and Bruker Fourier 7.1 T (300 MHz) spectrometers. Compounds were solubilized in deuterated chloroform ($CDCl_3$). The chemical shifts (δ) and coupling constants (J) were expressed in ppm and Hz, respectively. Multiplicities were reported as singlet (s), doublet (d), doublet of doublet (dd), doublet of doublet of doublets (ddd) and multiplet (m).

1.1. (2*E*,4*E*)-1,5-diphenylpenta-2,4-dien-1-one (**2**)

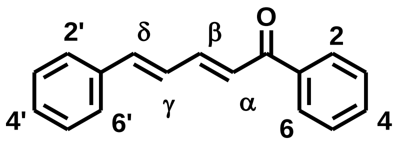

2

Pale yellow crystal

Yield: 33 %

Purity: 99.1%

Melting point: 99–102 °C

UV-Vis: λ_{max} 345 nm

^{1}H NMR (400 MHz): 8.01 (d; 7.2; H-2 and H-6); 7.67–7.50 (H-β, H-3, H-4, H-5, H-2' and H-6'), 7.42–7.35 (H-3', H-4' and H-5), 7.12 (d; 15.2; H-α), 7.06–7.05 (H-γ and H-δ).

^{13}C NMR (100 MHz): 190.6 (C=O), 144.9 (C-β), 142.0 (C-δ), 138.2 (C-1), 136.1 (C-1’), 132.7 (C-4), 129.2 (C-α), 128.9 (C-2 and C-6), 128.6 (C-3’ and C-5’), 128.4 (C-3 and C-5), 127.3 (C-2 and C-6), 126.7 (C-4’), 125.5 (C-γ).

1.2. (2*E*,4*E*)-1-(3-hydroxyphenyl)-5-phenylpenta-2,4-dien-1-one (**3**)

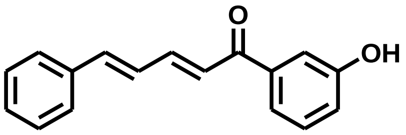

3

Brown crystal

Yield: 91 %

Purity: 98.4%

Melting point: 192−195 °C

UV-Vis: λ_{max} 346 nm

^{1}H NMR (600 MHz): 7.66−7.62 (m; H-β), 7.57 (dd; 1.8 and 1.8; H-2), 7.56 (d; 7.8; H-6), 7.53 (dd; 1.2 and 7.8; H-2’ and H-6’), 7.42−7.36 (H-5, H-3’, H-4’ and H-5’), 7.12−7.11 (m; H-4), 7.10 (d; 15, H-α), 7.06−7.05 (H-γ and H-δ), 6.06 (s; 3-OH).

^{13}C NMR (150 MHz): 190.8 (C=O), 156.5 (C-3), 145.5 (C-β), 142.5 (C-δ), 139.5 (C-1), 136.0 (C-1’), 129.9 (C-5), 129.4 (C-α), 128.9 (C-3’ and C-5’), 127.4 (C-2’ and C-6’), 126.8 (C-4’), 125.2 (C-γ), 120.9 (C-6), 120.4 (C-4), 115.1 (C-2).

1.3. (2*E*,4*E*)-1-(4-hydroxyphenyl)-5-phenylpenta-2,4-dien-1-one (**4**)

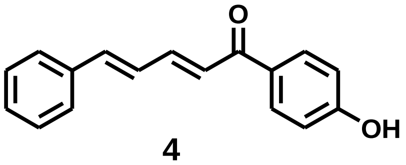

4

Yellow solid

Yield: 82 %

Purity: 96.7%

Melting point: 197−200 °C

UV-Vis: λ_{max} 350 nm

^{1}H NMR (600 MHz): 7.98 (d; 8.4; H-2 and H-6), 7.65−7.61 (m; H-β), 7.52 (dd; 1.2 and 7.8; H-2’ and H-6’), 7.41−7.35 (H-3’, H-4’ and H-5’), 7.14 (d; 14.4; H-α), 7.05−7.04 (H-γ and H-δ), 6.97 (d; 8.4; H-3 and H-5).

^{13}C NMR (150 MHz): 189.3 (C=O), 160.5 (C-4), 144.6 (C-β), 141.8 (C-δ), 136.1 (C-1’), 131.1 (C-2 and C-6), 130.9 (C-1), 129.2 (C-α), 128.9 (C-3’ and C-5’), 127.3 (C-2’ and C-6’), 127.0 (C-4’), 125.2 (C-γ), 115.6 (C-3 and C-5).

1.4. (2*E*,4*E*)-1-(3-aminophenyl)-5-phenylpenta-2,4-dien-1-one (**5**)

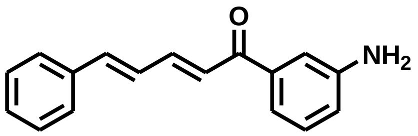

5

Yellow solid

Yield: 35 %

Purity: 99.8%

Melting point: 168−171 °C

UV-Vis: λ_{max} 343 nm

^{1}H NMR (600 MHz): 7.62−7.58 (m; H-β), 7.52 (dd; 1.2 and 7.8; H-2’ and H-6’), 7.41−7.33 (H-2, H-6, H-3’, H-4’ and H-5’), 7.29 (dd; 7.8 and 7.8; H-5), 7.07 (d; 15; H-α), 7.03−7.02 (H-γ and H-δ), 6.92 (ddd; 0.6, 2.4 and 7.8; H-4), 3.32 (s; 3-NH_2).

^{13}C NMR (150 MHz): 190.7 (C=O), 146.6 (C-3), 144.7 (C-β), 141.8 (C-δ), 139.3 (C-1), 136.1 (C-1’), 129.4 (C-5), 129.2 (C-α), 128.9 (C-3’ and C-5’), 127.3 (C-2’ and C-6’), 127.0 (C-4’), 125.7 (C-γ), 119.5 (C-4), 118.9 (C-6), 114.5 (C-2).

1.5. (2*E*,4*E*)-1-(3-methoxyphenyl)-5-phenylpenta-2,4-dien-1-one (**6**)

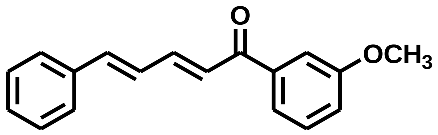

6

Yellow crystal

Yield: 78 %

Purity: 98.7%

Melting point: 84–87 °C

UV-Vis: λ_{max} 349 nm

^{1}H NMR (600 MHz): 7.66–7.61 (m; H-β), 7.58 (ddd; 1.2, 1.2 and 7.6; H-6), 7.54–7.53 (H-5, H-2' and H-6'), 7.44–7.34 (H-2, H-3', H-4'and H-5'), 7.15 (ddd; 1.2, 2.6 and 8.2; H-4), 7.10 (d; 15; H-α), 7.06–7.05 (H-γ and H-δ), 3.90 (s; 3-OCH_3).

^{13}C NMR (150 MHz): 190.2 (C=O), 159.9 (C-3), 144.9 (C-β), 142.0 (C-δ), 139.6 (C-1), 136.1 (C-1'), 129.6 (C-5), 129.3 (C-α), 128.9 (C-3' and C-5'), 127.3 (C-2' and C-6'), 126.7 (C-4'), 125.4 (C-γ), 121.0 (C-6), 119.3 (C-4), 112.6 (C-2), 55.5 (3-OCH_3).

1.6. (2*E*,4*E*)-1-(4-methoxyphenyl)-5-phenylpenta-2,4-dien-1-one (**7**)

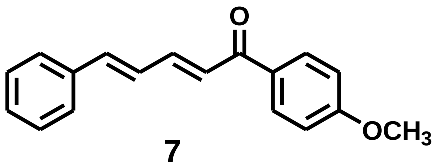

7

Yellow crystal

Yield: 35 %

Purity: 99.0%

Melting point: 91–93 °C

UV-Vis: λ_{max} 349 nm

^{1}H NMR (400 MHz): 8.02 (d; 9.0; H-2 and H-6), 7.65–7.59 (m; H-β), 7.52 (dd; 1.4 and 8.2; H-2’ and H-6’), 7.42–7.34 (H-3’, H-4’ and H-5’), 7.13 (d; 14.8; H-α), 7.05–7.03 (H-γ and H-δ), 7.00 (d; 9.0; H-3 and H-5), 3.91 (s; 4-OCH$_3$).

^{13}C NMR (100 MHz): 188.7 (C=O), 163.3 (C-4), 144.0 (C-β), 141.4 (C-δ), 136.2 (C-1’), 131.5 (C-1), 130.7 (C-2 and C-6), 129.1 (C-α), 128.8 (C-3’ and C-5’), 127.2 (C-2’ and C-6’), 127.1 (C-4’), 125.3 (C-γ), 113.8 (C-3 and C-5), 55.5 (4-OCH$_3$).

1.7. (2*E*,4*E*)-1-(3,4-methylenedioxy)-5-phenylpenta-2,4-dien-1-one (**8**)

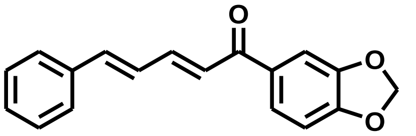

8

Yellow crystal

Yield: 83 %

Purity: 99.8%

Melting point: 123–125 °C

UV-Vis: λ_{max} 356 nm

^{1}H NMR (600 MHz): 7.62 (dd; 1.5 and 8.1; H-2’ and H-6’), 7.63–7.59 (m; H-β), 7.53–7.51 (H-2 and H-6), 7.40 (dd; 7.2 and 8.1; H-3’ and H-5’), 7.36–7.33 (m; H-4’), 7.08 (d; 15; H-α), 7.04–7.03 (H-γ and H-δ), 6.91 (d; 8.4; H-5), 6.08 (s; –OCH$_2$O–).

^{13}C NMR (150 MHz): 188.2 (C=O), 151.6 (C-4), 148.3 (C-3), 144.3 (C-β), 141.6 (C-δ), 136.2 (C-1’), 133.0 (C-1), 129.2 (C-α), 128.9 (C-3’ and C-5’), 127.3 (C-2’ and C-6’), 127.0 (C-4’), 125.1 (C-γ), 124.5 (C-6), 108.4 (C-5), 107.9 (C-2), 101.9 (–OCH$_2$O–).

1.8. (2*E*,4*E*)-1-(4-hydroxy-3-methoxyphenyl)-5-phenylpenta-2,4-dien-1-one (**9**)

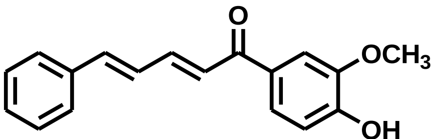

9

Yellow crystal

Yield: 98 %

Purity: 98.5%

Melting point: 207–210 °C

UV-Vis: λ_{max} 356 nm

^{1}H NMR (300 MHz): 7.66–7.58 (H-β, H-2 and H-6), 7.52 (dd; 1.5 and 8.1; H-2’ and H-6’), 7.42–7.33 (H-3’, H-4’ and H-5’), 7.14 (d; 15; H-α), 7.05–7.03 (H-γ and H-δ), 7.00 (d; 8.4; H-5), 6.10 (s; 4-OH), 4.00 (s; 3-OCH_3).

^{13}C NMR (75 MHz): 188.5 (C=O), 150.3 (C-3), 146.8 (C-4), 144.0 (C-β), 141.5 (C-δ), 136.2 (C-1’), 131.1 (C-1), 129.1 (C-α), 128.9 (C-3’ and C-5’), 127.3 (C-2’ and C-6’), 127.1 (C-4’), 125.0 (C-γ), 123.5 (C-6), 113.8 (C-5), 110.4 (C-2), 56.1 (4-OCH_3).

1.9. (2*E*,4*E*)-1-(4-fluorophenyl)-5-phenylpenta-2,4-dien-1-one (**10**)

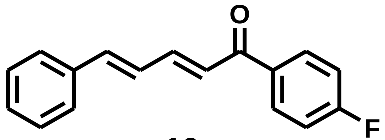

10

Yellow solid

Yield: 71 %

Purity: 99.1%

Melting point: 88–91 °C

UV-Vis: λ_{max} 346 nm

^{1}H NMR (300 MHz): 8.03 (d; 8.7; H-2 and H-6), 7.67–7.58 (m; H-β), 7.52 (dd; 1.3 and 7.9; H-2’ and H-6’), 7.43–7.35 (H-3’, H-4’, H-5’), 7.18 (dd; 8.7 and 9.0; H-3 and H-5), 7.11–7.03 (H-α, H-γ and H-δ).

^{13}C NMR (75 MHz): 188.8 (C=O), 165.5 (d; J_{CF} = 252 Hz; C-4), 145.1 (C-β), 142.2 (C-δ), 136.0 (C-1’), 134.5 (C-1), 131.0 (d; J_{CF} = 9.0 Hz; C-2 and C-6), 129.3 (C-α), 128.9 (C-3’ and C-5’), 127.3 (C-2’ and C-6’), 126.8 (C-4’), 124.9 (C-γ), 115.7 (d; J_{CF} = 21 Hz; C-3 and C-5).

1.10. (2*E*,4*E*)-1-(4-chlorophenyl)-5-phenylpenta-2,4-dien-1-one (**11**)

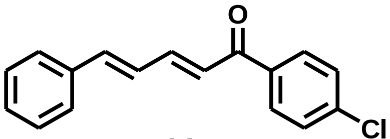

11

Yellow solid

Yield: 72 %

Purity: 99.8%

Melting point: 124–127 °C

UV-Vis: λ_{max} 349 nm

^{1}H NMR (300 MHz): 7.94 (d; 8.7; H-2 and H-6), 7.67–7.59 (m; H-β), 7.54–7.35 (H-3, H-5, H-2’, H-3’, H-4’, H-5’ and H-6’), 7.09–7.03 (H-α, H-γ and H-δ).

^{13}C NMR (75 MHz): 189.2 (C=O), 145.4 (C-β), 142.5 (C-δ), 139.1 (C-4), 136.5 (C-1), 136.0 (C-1’), 129.8 (C-2 and C-6), 129.4 (C-α), 128.9 (C-3, C-5, C-3’ and C-5’), 127.4 (C-2’ and C-6’), 126.8 (C-4’), 124.8 (C-γ).

1.11. (2*E*,4*E*)-1-(4-bromophenyl)-5-phenylpenta-2,4-dien-1-one (**12**)

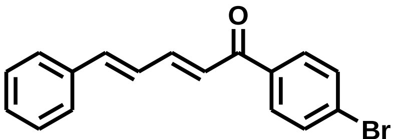

12

Yellow solid

Yield: 86 %

Purity: 98.7 %

Melting point: 142−145 °C

UV-Vis: λ_{max} 350 nm

^{1}**H NMR (300 MHz):** 7.86 (d; 8.5; H-2 and H-6), 7.65 (d; 8.5; H-3 and H-5), 7.62−7.59 (m; H-β), 7.52 (dd; 1.5 and 8.1; H-2' and H-6'), 7.43−7.35 (H-3', H-4' and H-5'), 7.08−7.03 (H-α, H-γ and H-δ).

^{13}**C NMR (75 MHz):** 189.4 (C=O), 145.4 (C-β), 142.5 (C-δ), 136.9 (C-1), 136.0 (C-1'), 131.9 (C-3 and C-5), 129.9 (C-2 and C-6), 129.4 (C-α), 128.9 (C-3' and C-5'), 127.8 (C-4), 127.4 (C-2' and C-6'), 126.8 (C-4'), 124.7 (C-γ).

1.12. (2*E*,4*E*)-1-(3,4-dichlorophenyl)-5-phenylpenta-2,4-dien-1-one (**13**)

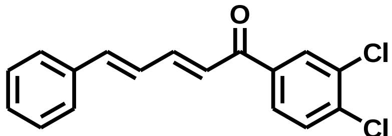

13

Yellow crystal

Yield: 79 %

Purity: 97.5%

Melting point: 165−167 °C

UV-Vis: λ_{max} 353 nm

^{1}H NMR (600 MHz): 8.08 (d; 2.1; H-2), 7.82 (dd; 2.1 and 8.4; H-6), 7.67–7.63 (m; H-β), 7.59 (d; 8.4; H-5), 7.53 (dd; 1.2 and 7.8; H-2' and H-6'), 7.42–7.35 (H-3', H-4' and H-5'), 7.10–7.02 (H-α, H-γ and H-δ).

^{13}C NMR (150 MHz): 187.9 (C=O), 146.0 (C-β), 143.1 (C-δ), 137.8 (C-4), 137.2 (C-1), 135.9 (C-3), 133.2 (C-1'), 130.7 (C-2), 130.4 (C-5), 129.6 (C-α), 128.9 (C-3' and C-5'), 127.4 (C-6, C-2' and C-6'), 126.6 (C-4'), 124.1 (C-γ).

1.13. (2*E*,4*E*)-1-(3-(trifluoromethyl)phenyl)-5-phenylpenta-2,4-dien-1-one (**14**)

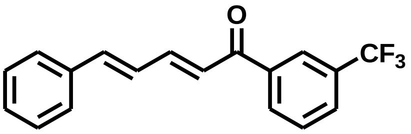

14

Yellow solid

Yield: 88%

Purity: 96.1%

Melting point: 126–130 °C

UV-Vis: λ_{max} 351 nm

^{1}H NMR (600 MHz): 8.25 (m; H-2), 8.18 (d; 7.8; H-6), 7.85 (d; 7.2; H-4), 7.70–7.67 (m; H-β), 7.65 (d; 7.8; H-5), 7.54 (dd; 1.8 and 8.1; H-2' and H-6'), 7.43–7.36 (H-3', H-4' and H-5'), 7.12 (d; 15.0; H-α), 7.09–7.08 (H-γ and H-δ).

^{13}C NMR (150 MHz): 189.0 (C=O), 146.1 (C-β), 143.3 (C-δ), 138.8 (C-1), 135.9 (C-1'), 131.5 (C-6), 131.2 (d; J_{CF} = 33 Hz; C-3), 129.5 (C-α), 129.3 (C-5), 129.1 (C-4), 128.9 (C-3' and C-5'), 127.4 (C-2' and C-6'), 126.7 (C-4'), 125.2 (C-2), 124.4 (C-γ), 123.8 (d; J_{CF} = 271 Hz; 4-CF_3).

1.14. (2*E*,4*E*)-1-(3-nitrophenyl)-5-phenylpenta-2,4-dien-1-one (**15**)

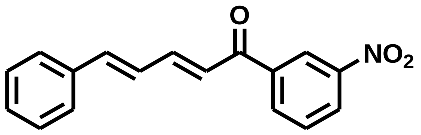

15

Yellow solid

Yield: 85 %

Purity: 97.8%

Melting point: 128–130 °C

UV-Vis: λ_{max} 354 nm

^{1}H NMR (400 MHz): 8.82 (dd; 2.0 and 2.0; H-2), 8.45 (ddd; 1.2, 2.0 and 8.0; H-4), 8.33 (ddd; 1.2, 2.0 and 7.8; H-6), 7.72 (dd; 7.8 and 8.0; H-5), 7.74–7.68 (m; H-β), 7.55 (dd; 1.4 and 8.2; H-2’ and H-6’), 7.44–7.38 (H-3’, H-4’ and H-5’), 7.13 (d; 15.6; H-α), 7.11–7.09 (H-γ and H-δ).

^{13}C NMR (100 MHz): 187.9 (C=O), 148.4 (C-3), 146.7 (C-β), 143.6 (C-δ), 139.6 (C-1), 135.8 (C-1’), 134.0 (C-6), 129.9 (C-5), 129.7 (C-α), 129.0 (C-3’ and C-5’), 127.5 (C-2’ and C-6’), 126.9 (C-4’), 126.5 (C-4), 123.9 (C-γ), 123.2 (C-2).

1.15. (2*E*,4*E*)-1-(4-nitrophenyl)-5-phenylpenta-2,4-dien-1-one (**16**)

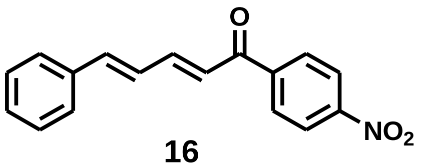

16

Yellow solid

Yield: 84 %

Purity: 97.7%

Melting point: 177–180 °C

UV-Vis: λ_{max} 361 nm

^{1}H NMR (400 MHz): 8.36 (d; 8.8; H-3 and H-5), 8.12 (d; 8.8; H-2 and H-6), 7.70–7.64 (m; H-β), 7.54 (dd; 1.8 and 8.2; H-2’ and H-6’), 7.44–7.38 (H-3’, H-4’ and H-5’), 7.10–7.05 (H-α, H-γ and H-δ).

^{13}C NMR (100 MHz): 188.9 (C=O), 149.9 (C-4), 146.8 (C-β), 143.7 (C-δ), 143.1 (C-1), 135.7 (C-1’), 129.8 (C-α), 129.3 (C-2 and C-6), 129.0 (C-3’ and C-5’), 127.5 (C-2’ and C-6’), 126.5 (C-4’), 124.5 (C-γ), 123.8 (C-3 and C-5).

1.16. (2*E*,4*E*)-1-(3-methylphenyl)-5-phenylpenta-2,4-dien-1-one (**17**)

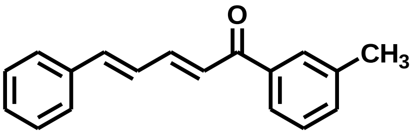

17

Orange oil

Yield: 49 %

Purity: 96.4%

UV-Vis: λ_{max} 344 nm

^{1}H NMR (600 MHz): 7.82 (m; H-2), 7.81–7.79 (m; H-6), 7.65–7.60 (m; H-β), 7.53 (dd; 1.2 and 7.8; H-2’ and H-6’), 7.42–7.39 (H-4, H-5, H-3’ and H-5’), 7.37–7.34 (m; H-4’), 7.12 (d; 15; H-α), 7.06–7.05 (H-γ and H-δ), 2.46 (s; 3-CH_3).

^{13}C NMR (150 MHz): 190.7 (C=O), 144.7 (C-β), 141.8 (C-δ), 138.4 (C-3), 138.3 (C-1), 136.1 (C-1’), 133.5 (C-4), 129.2 (C-α), 129.0 (C-2), 128.9 (C-3’ and C-5’), 128.5 (C-5), 127.3 (C-2’ and C-6’), 127.0 (C-4’), 125.6 (C-γ and C-6), 21.4 (3-CH_3).

1.17. (2*E*,4*E*)-1-(4-methylphenyl)-5-phenylpenta-2,4-dien-1-one (**18**)

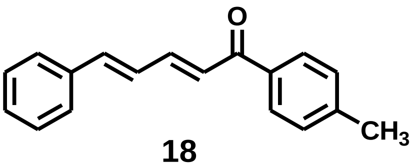

18

Yellow solid

Yield: 53 %

Purity: 99.9%

Melting point: 77–79 °C

UV-Vis: λ_{max} 346 nm

^{1}H NMR (300 MHz): 7.91 (d; 8.4; H-2 and H-6), 7.66–7.57 (m; H-β), 7.52 (dd; 1.5 and 8.1; H-2' and H-6'), 7.42–7.29 (H-3', H-4' and H-5'), 7.31 (d; 8.4; H-3 and H-5), 7.12 (d; 14.7; H-α), 7.05–7.03 (H-γ and H-δ), 2.45 (s; 4-CH_3).

^{13}C NMR (150 MHz): 190.0 (C=O), 144.5 (C-β), 143.5 (C-4), 141.7 (C-δ), 136.2 (C-1'), 135.6 (C-1), 129.3 (C-2 and C-6), 129.2 (C-α), 128.9 (C-3 and C-5), 128.6 (C-3' and C-5'), 127.3 (C-2' and C-6'), 127.0 (C-4'), 125.5 (C-γ), 21.7 (4-CH_3).

Figure S1. i) ^{1}H NMR spectrum of cinnamylideneacetophenone **2**

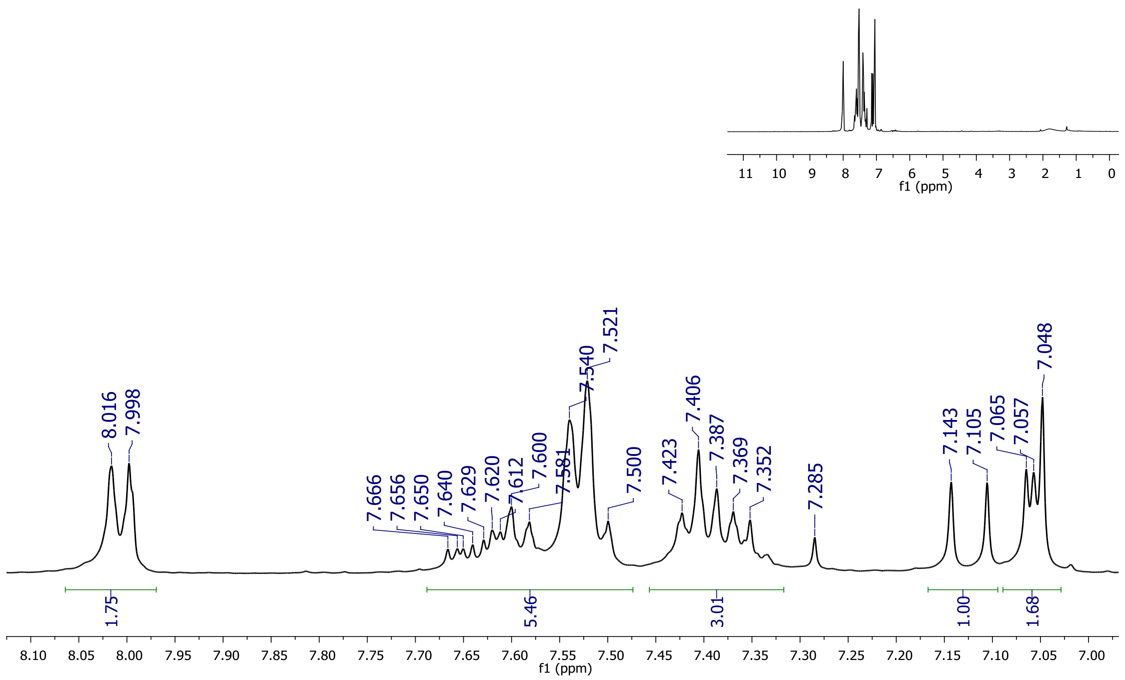

Figure S1. ii) ^{13}C NMR spectrum of cinnamylideneacetophenone **2**

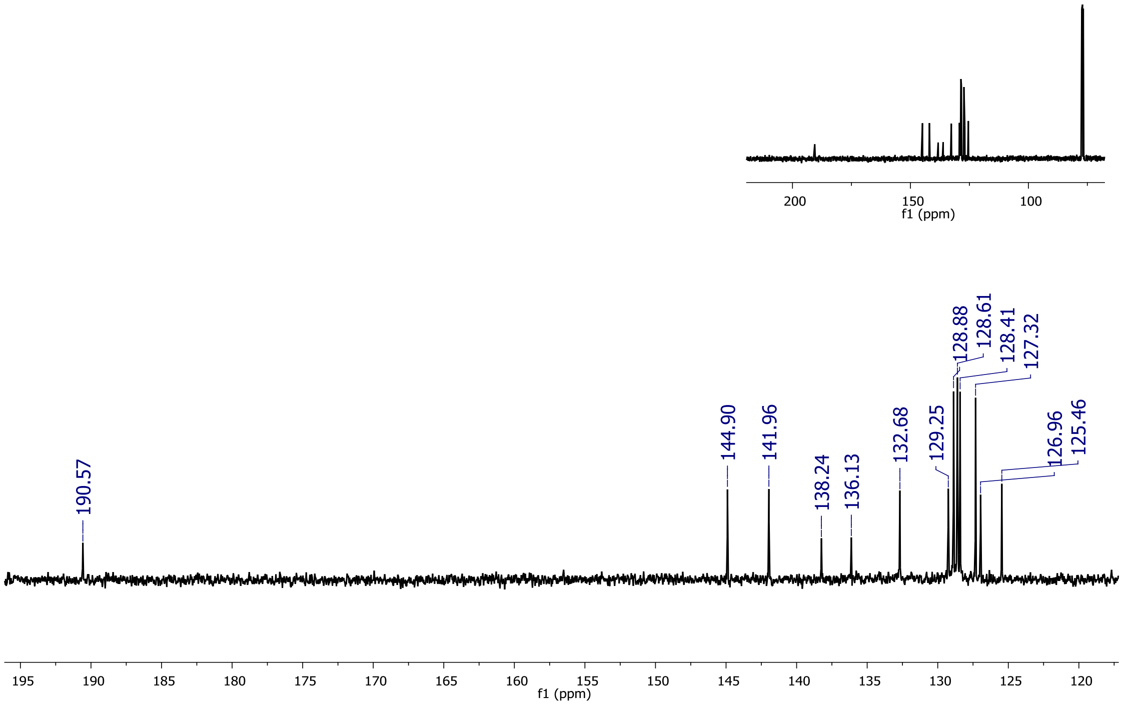

Figure S1. iii) UV-Vis spectrum of cinnamylideneacetophenone **2**, MeOH/H_2O (3:1)

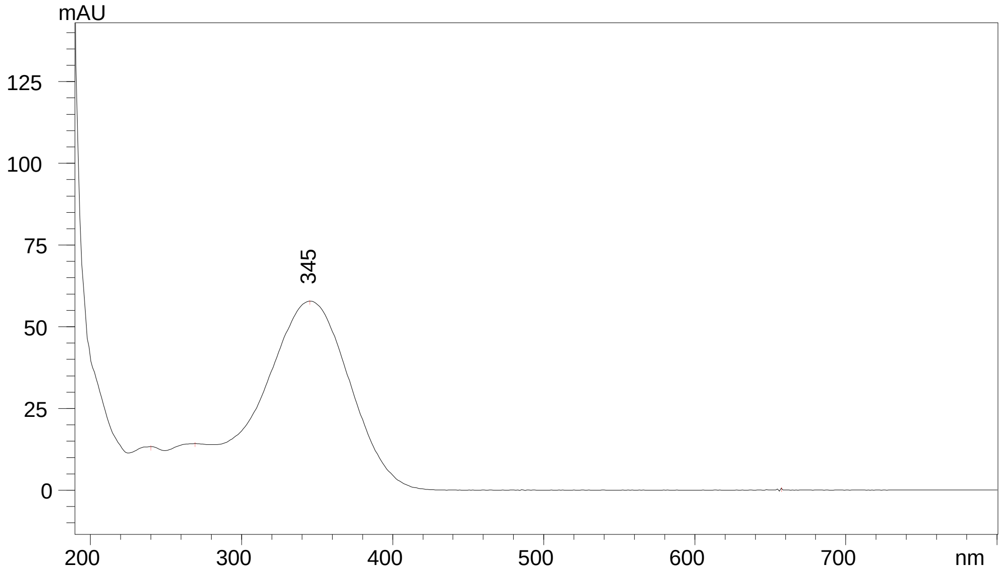

Figure S1. iv) HPLC chromatogram of cinnamylideneacetophenone **2**

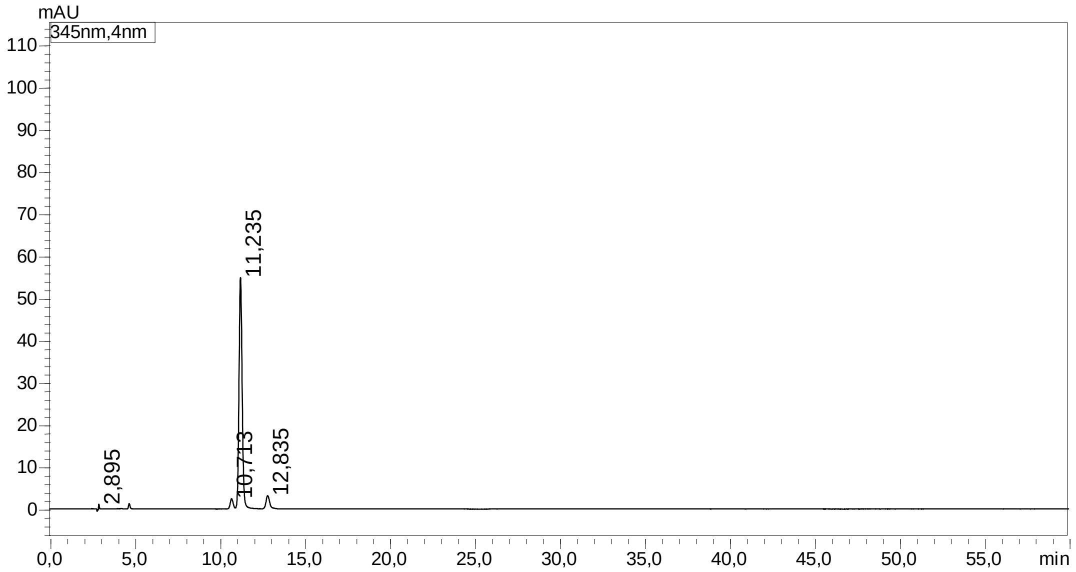

Figure S2. i) ^{1}H NMR spectrum of cinnamylideneacetophenone **3**

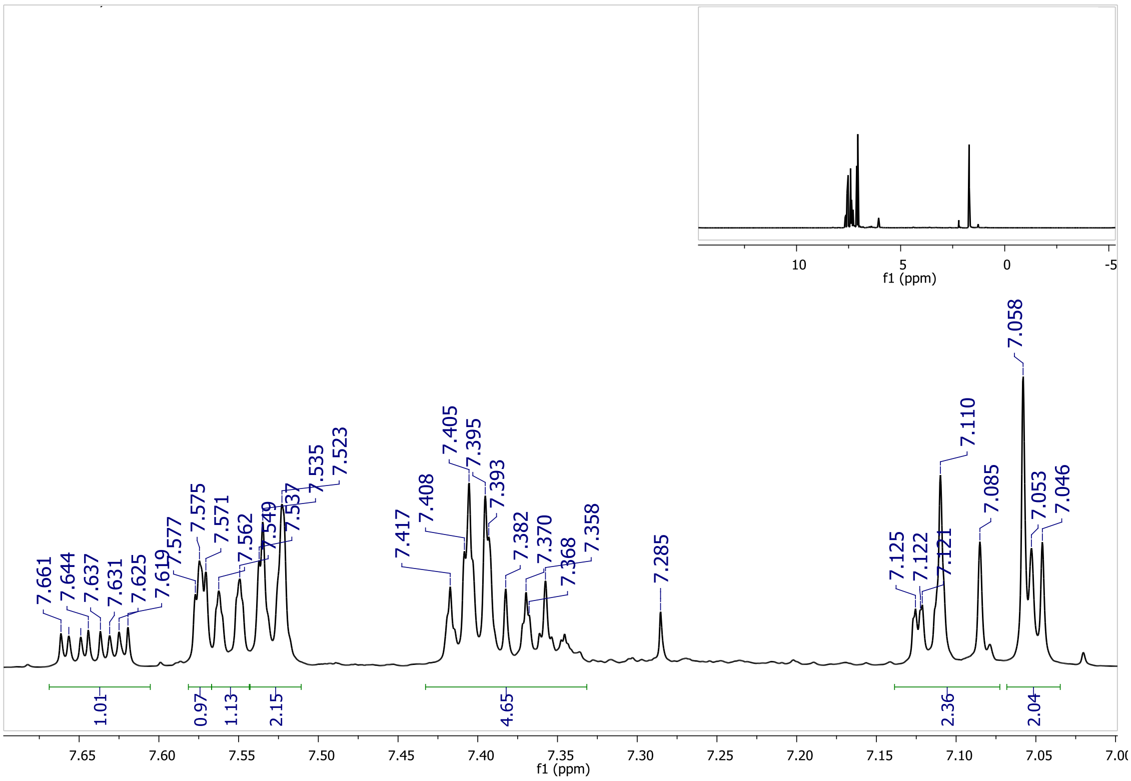

Figure S2. ii) ^{13}C NMR spectrum of cinnamylideneacetophenone **3**

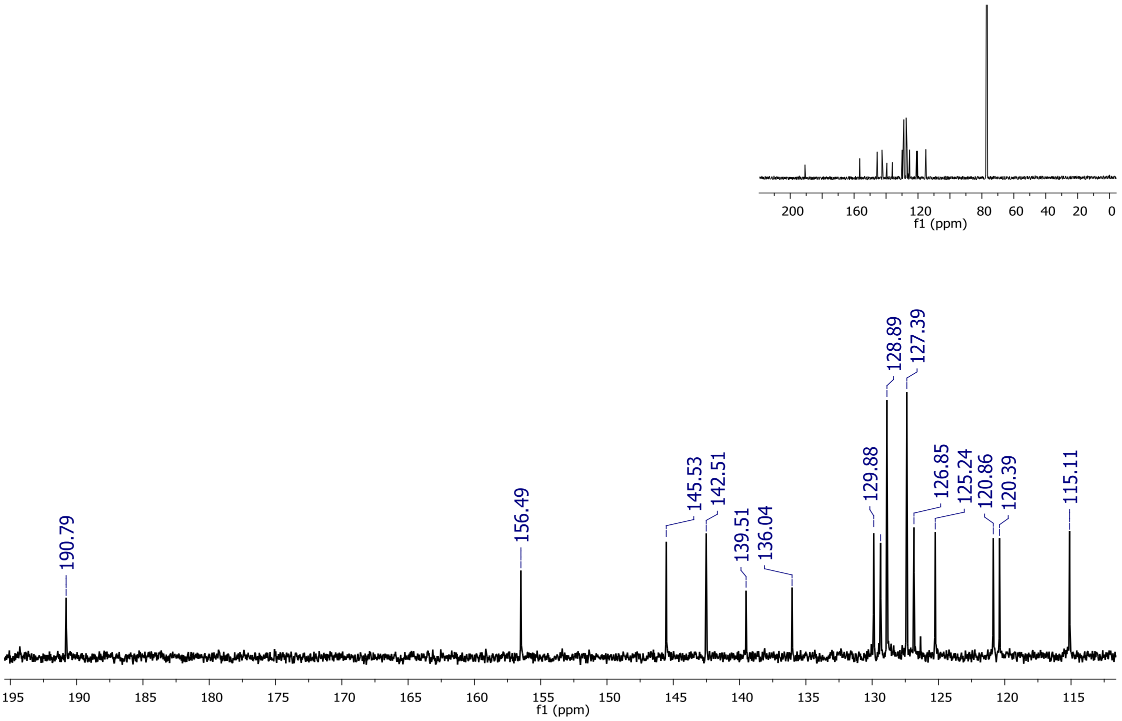

Figure S2. iii) UV-Vis spectrum of cinnamylideneacetophenone **3**, MeOH/H_2O (3:1)

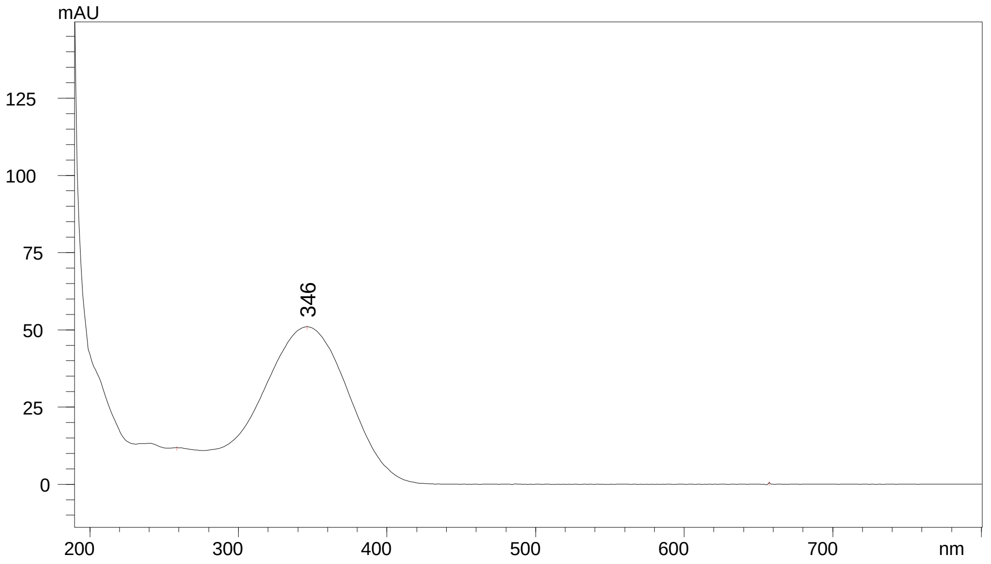

Figure S2. iv) HPLC chromatogram of cinnamylideneacetophenone **3**

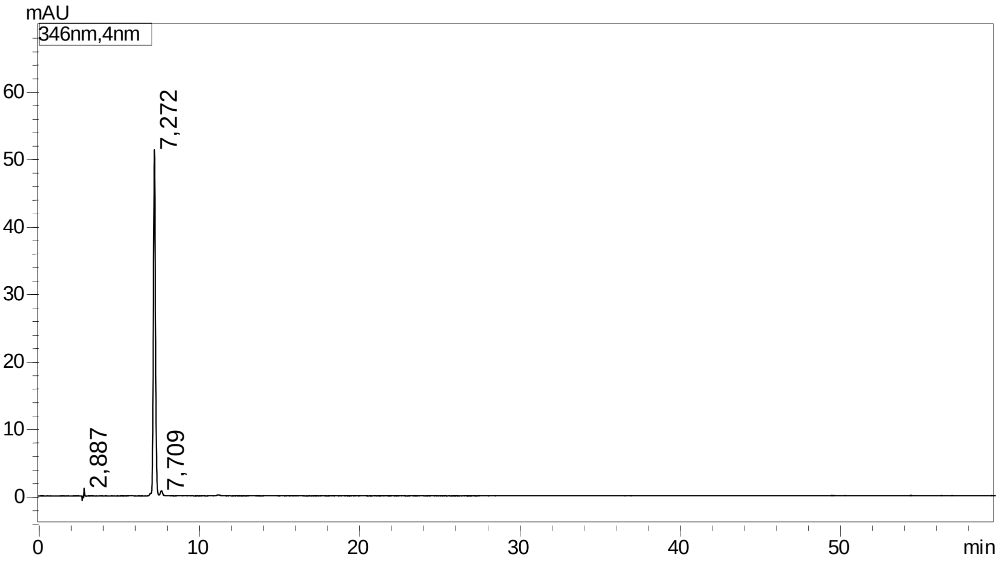

Figure S3. i) ^{1}H NMR spectrum of cinnamylideneacetophenone **4**

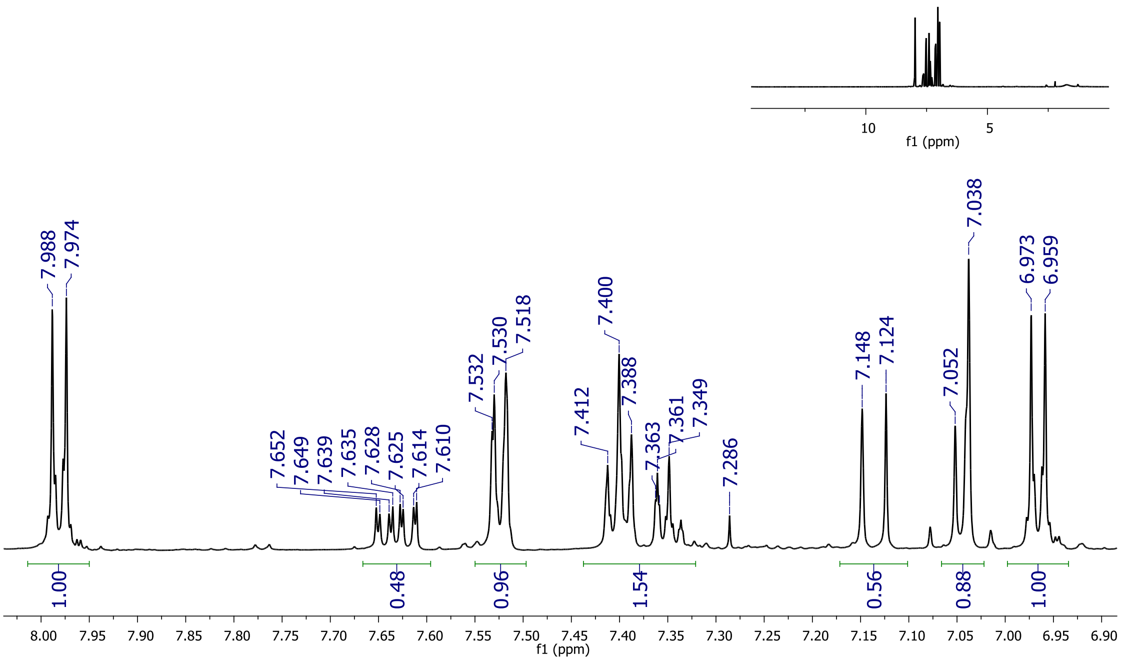

Figure S3. ii) ^{13}C NMR spectrum of cinnamylideneacetophenone **4**

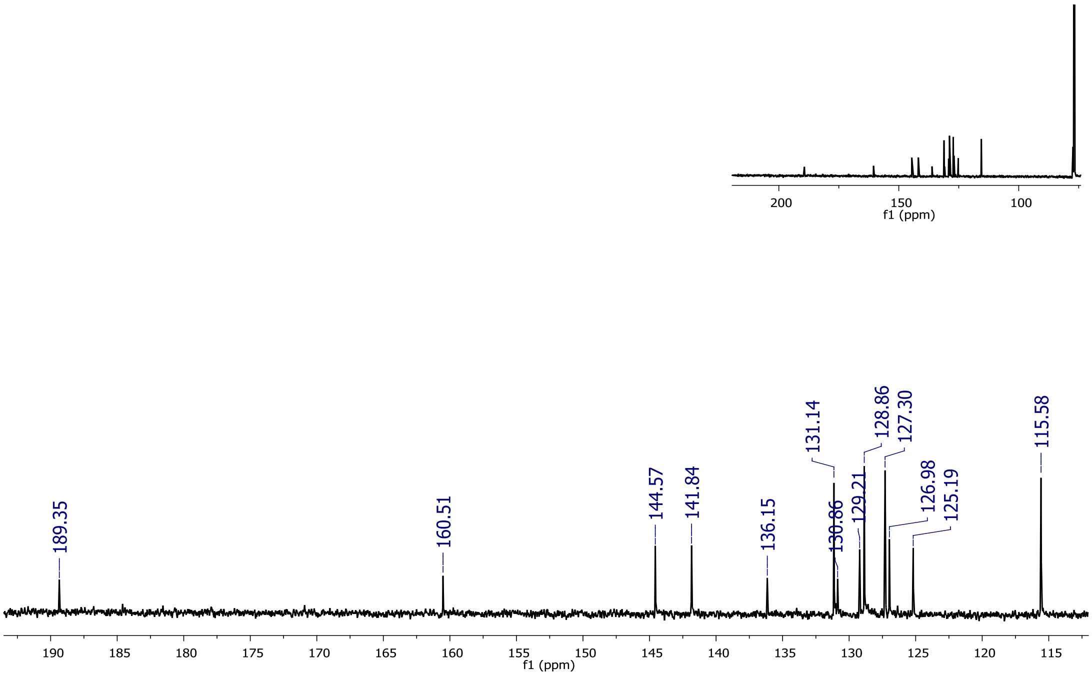

Figure S3. iii) UV-Vis spectrum of cinnamylideneacetophenone **4**, MeOH/H_2O (3:1)

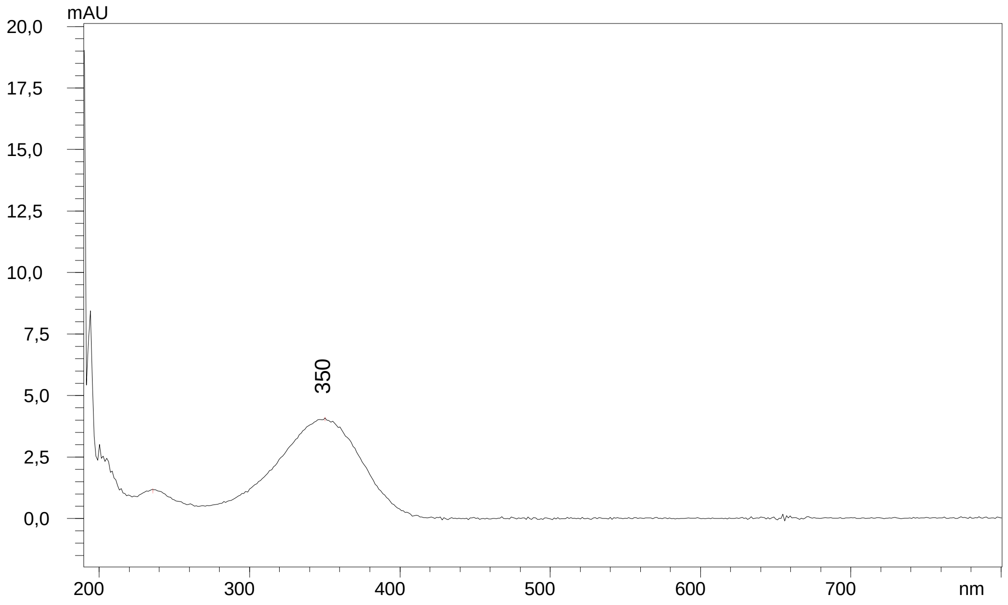

Figure S3. iv) HPLC chromatogram of cinnamylideneacetophenone **4**

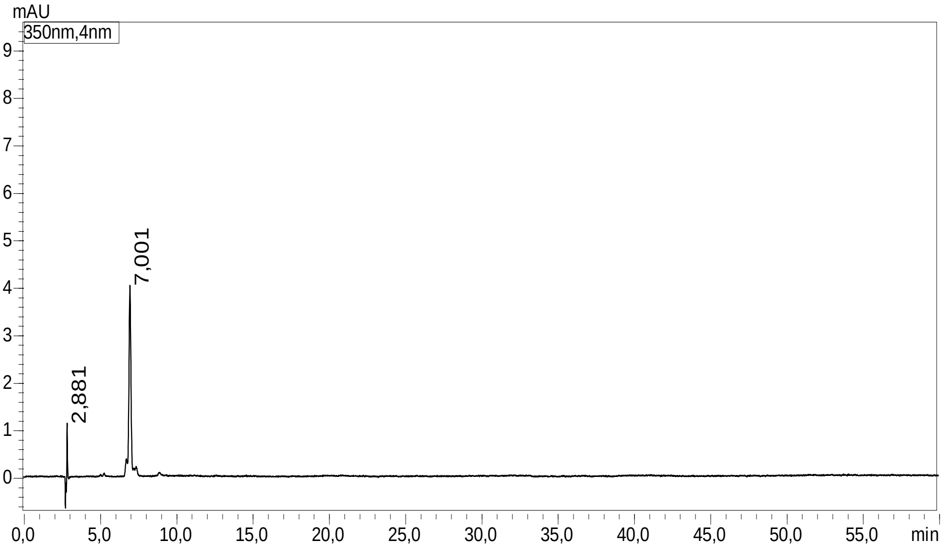

Figure S4. i) ^{1}H NMR spectrum of cinnamylideneacetophenone **5**

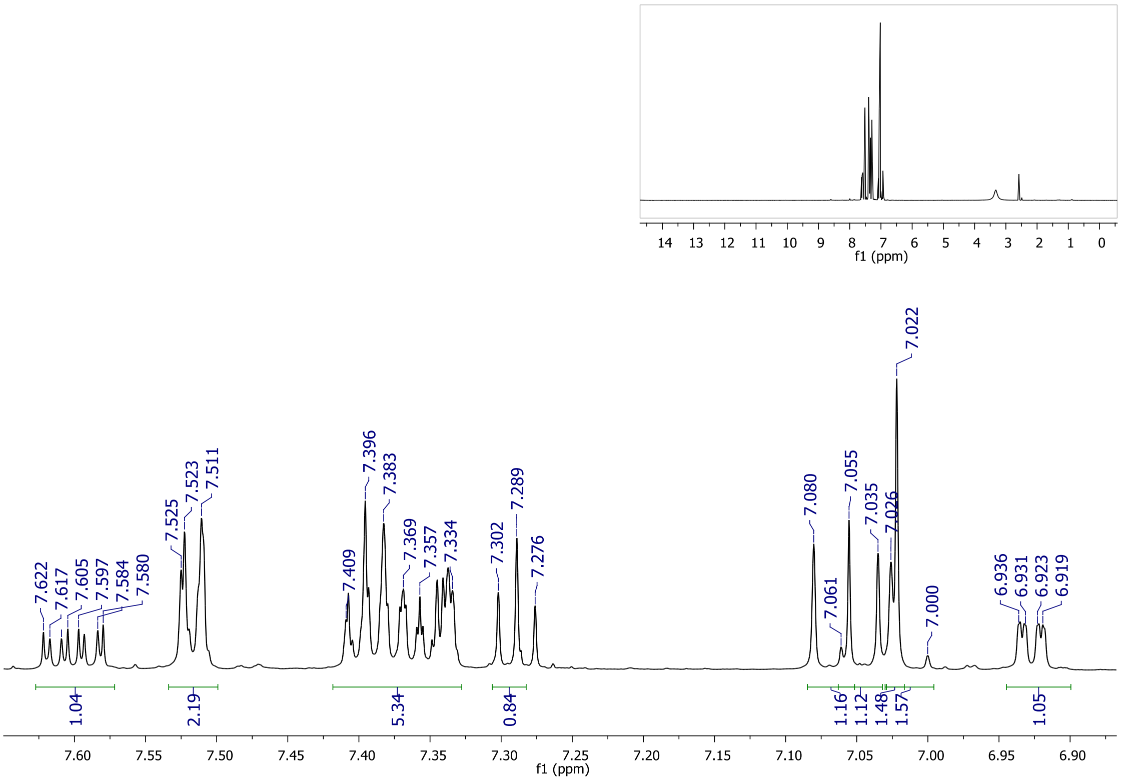

Figure S4. ii) ^{13}C NMR spectrum of cinnamylideneacetophenone **5**

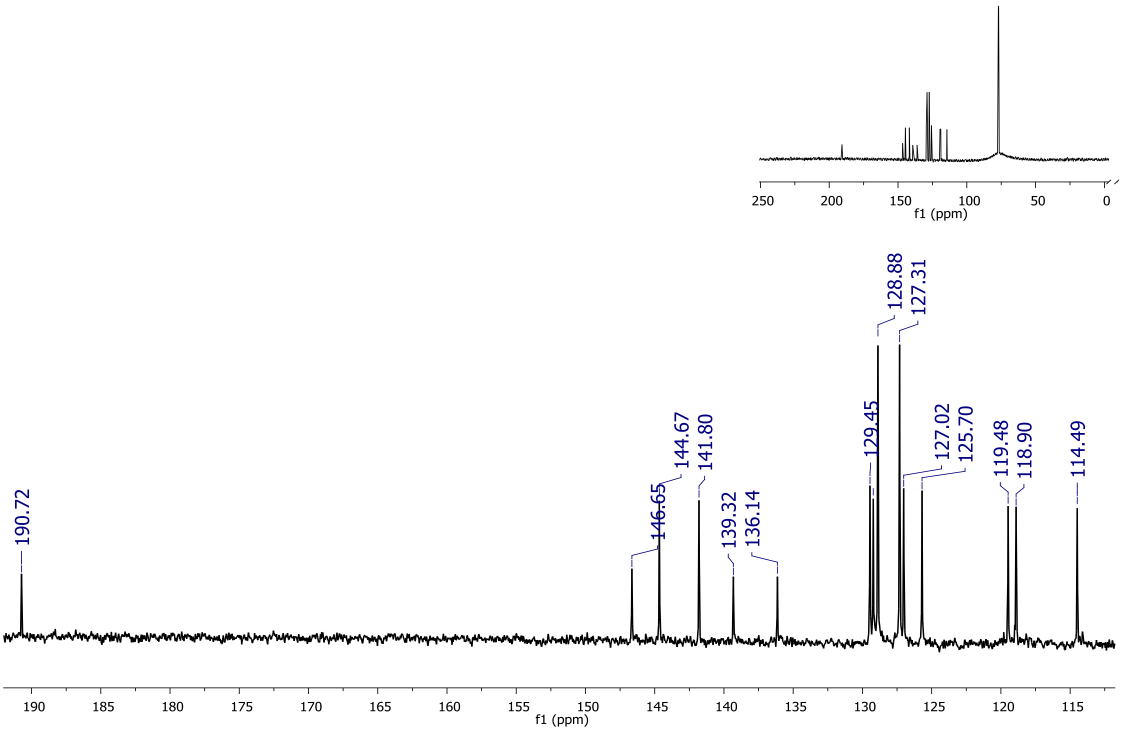

Figure S4. iii) UV-Vis spectrum of cinnamylideneacetophenone **5**, MeOH/H_2O (3:1)

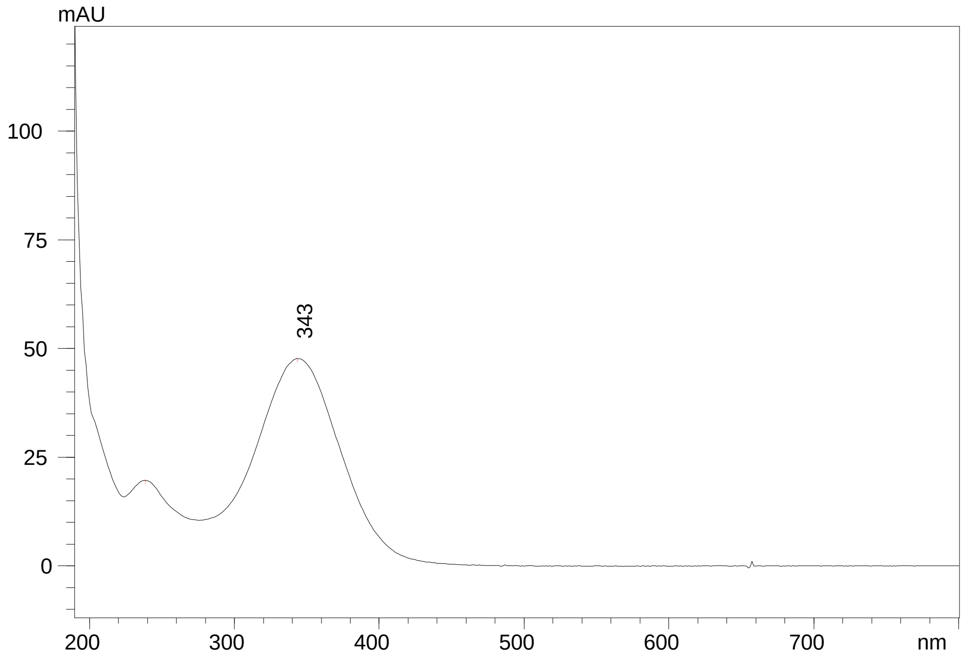

Figure S4. iv) HPLC chromatogram of cinnamylideneacetophenone **5**

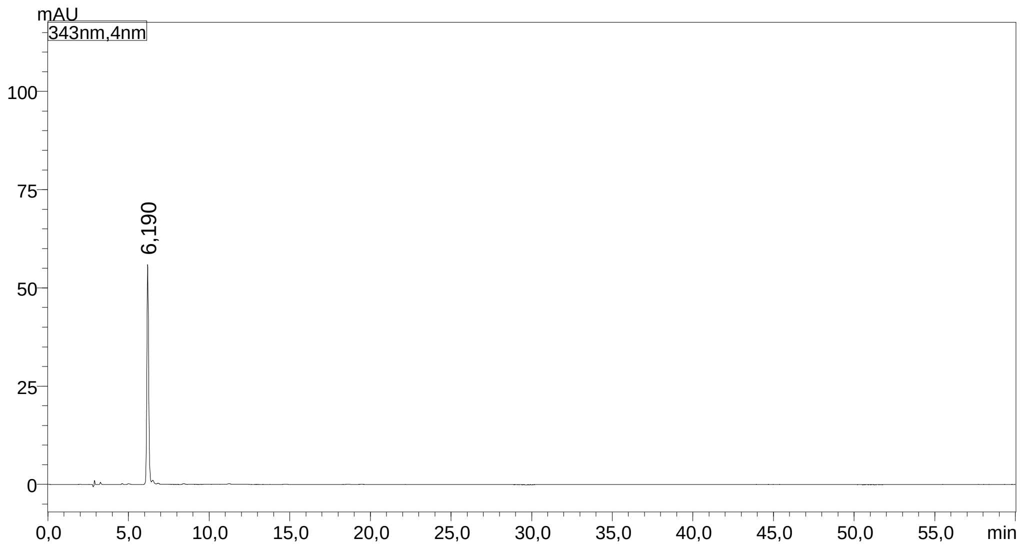

Figure S5. ^{1}H NMR spectrum of cinnamylideneacetophenone **6**

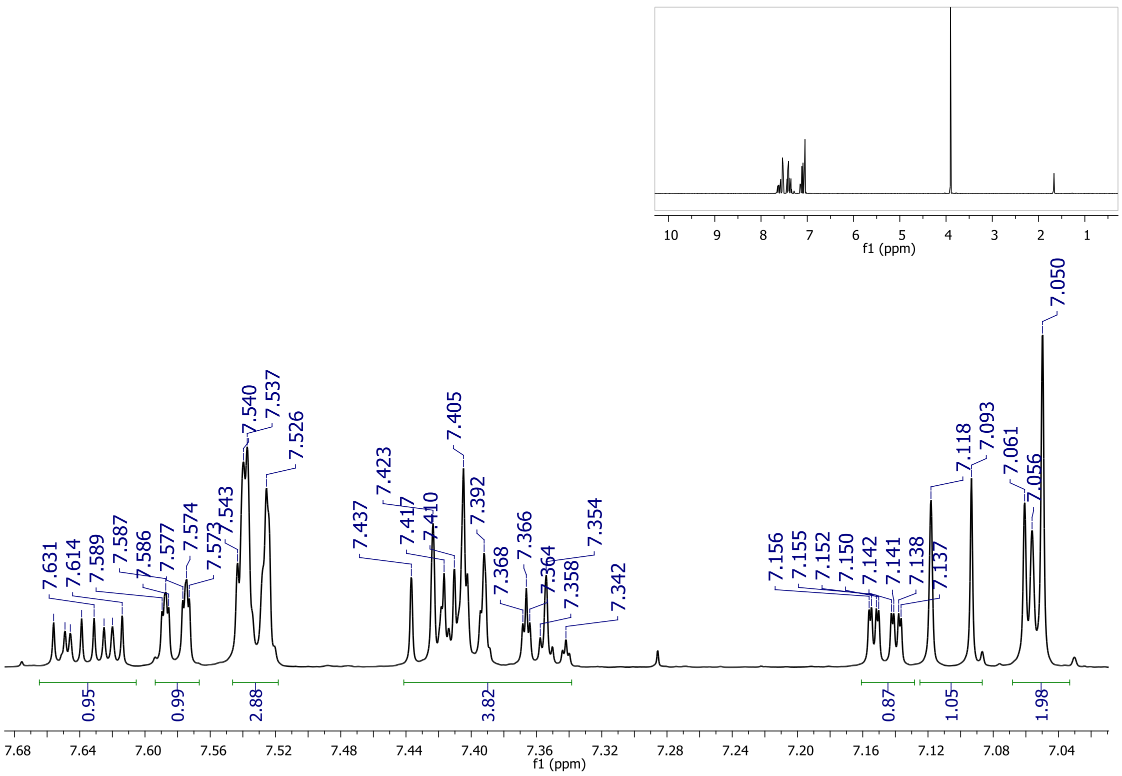

Figure S5. ii) ^{13}C NMR spectrum of cinnamylideneacetophenone **6**

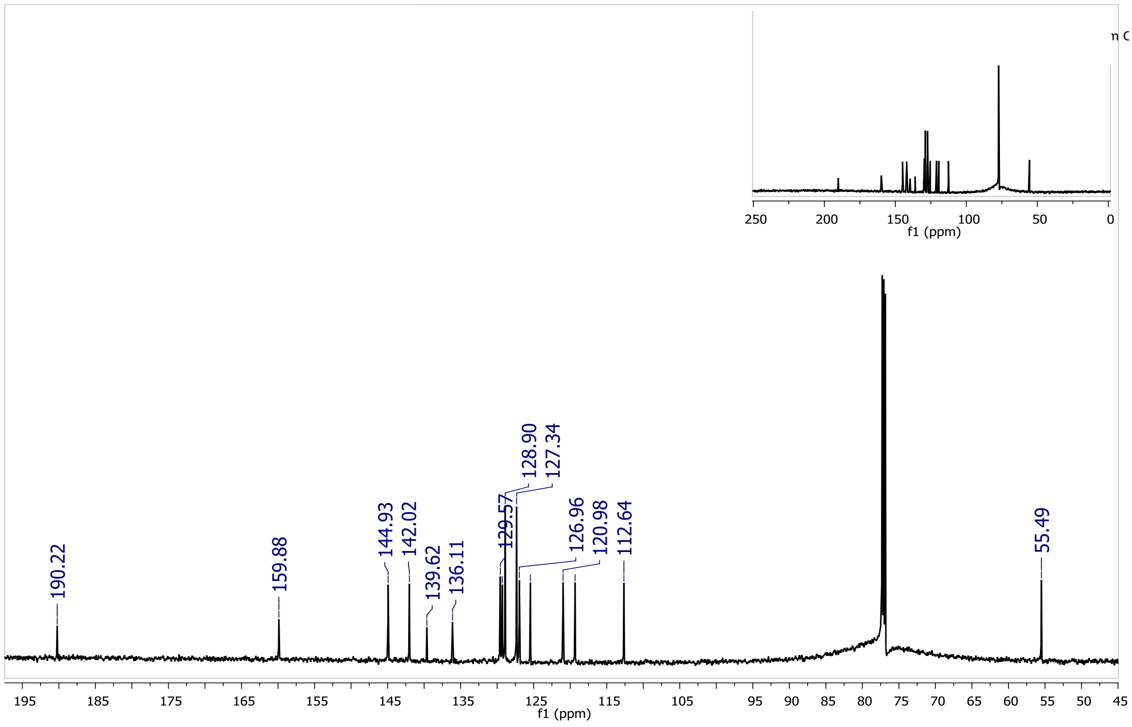

Figure S5. iii) UV-Vis spectrum of cinnamylideneacetophenone **6**, MeOH/H_2O (3:1)

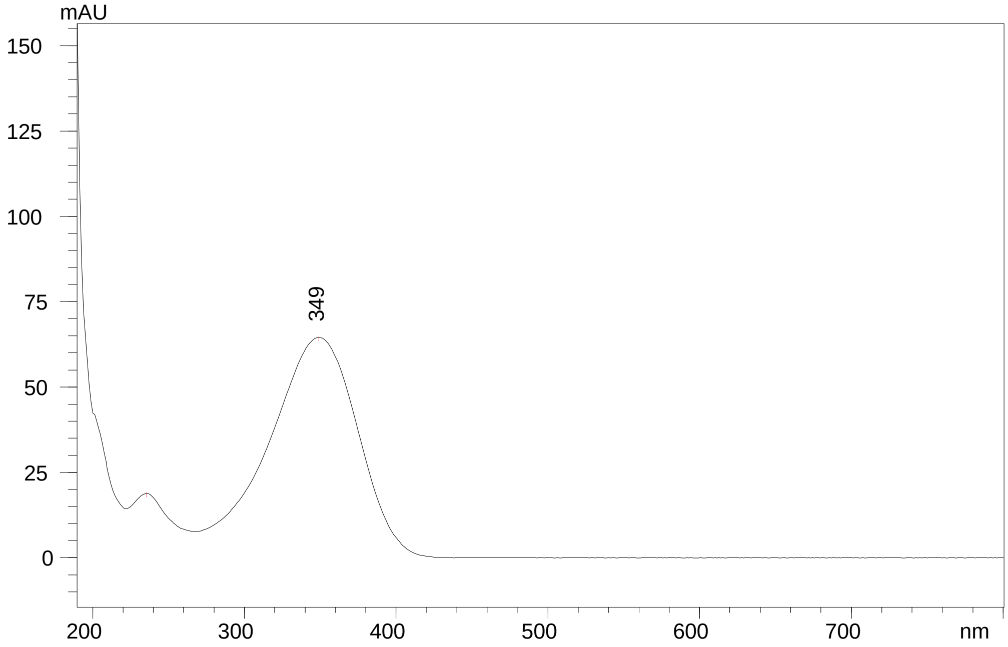

Figure S5. iv) HPLC chromatogram of cinnamylideneacetophenone **6**

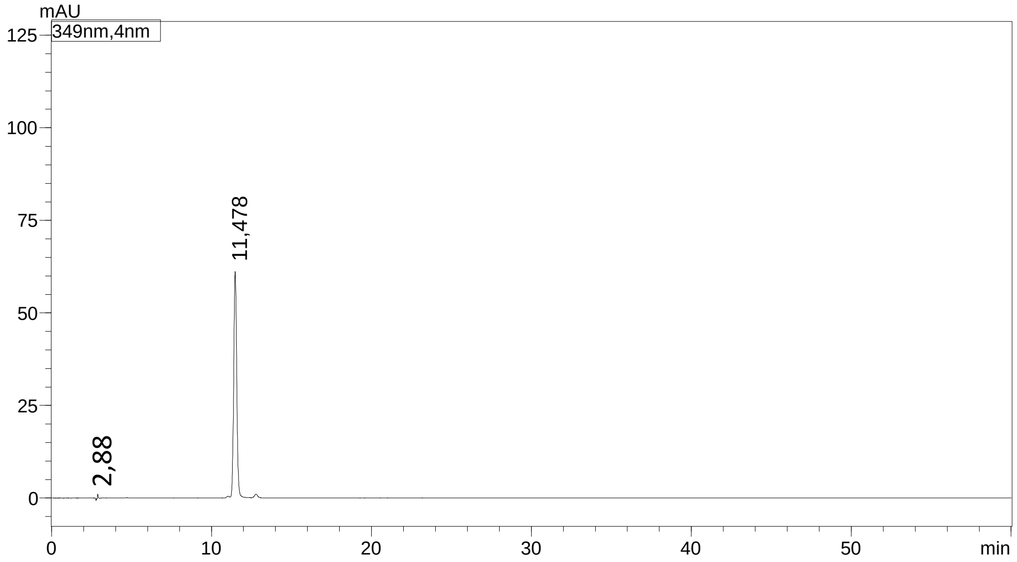

Figure S6. i) ^{1}H NMR spectrum of cinnamylideneacetophenone **7**

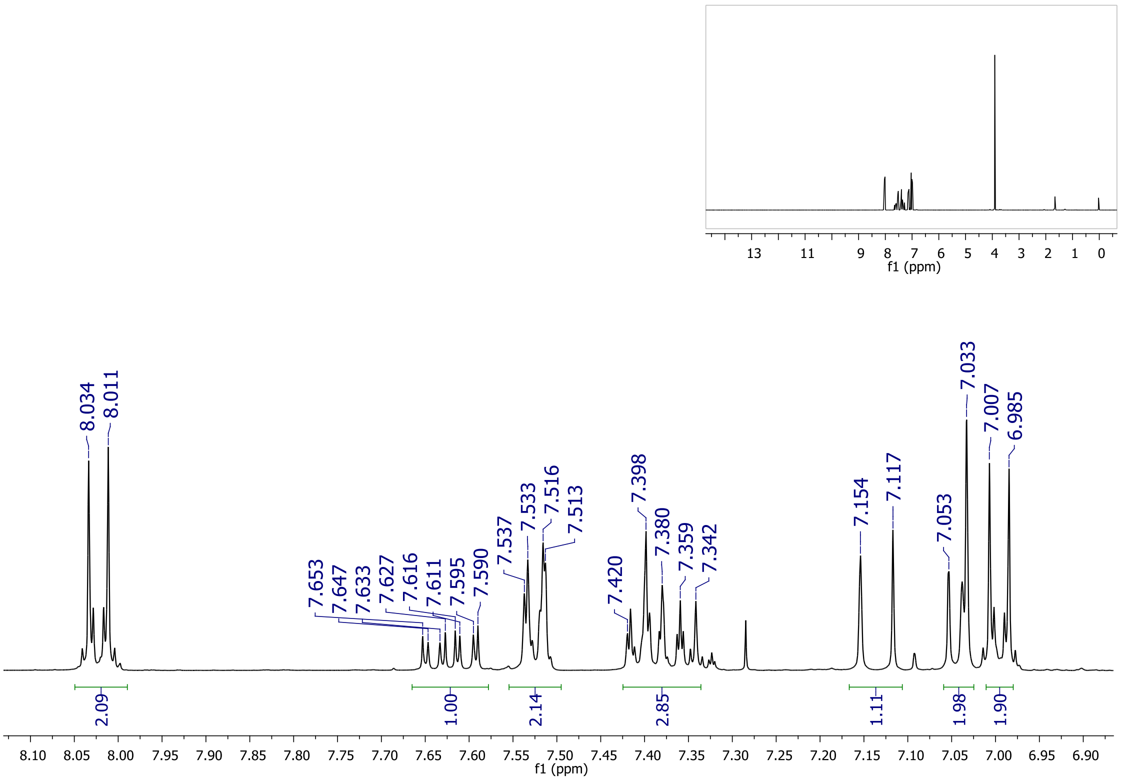

Figure S6. ii) ^{13}C NMR spectrum of cinnamylideneacetophenone **7**

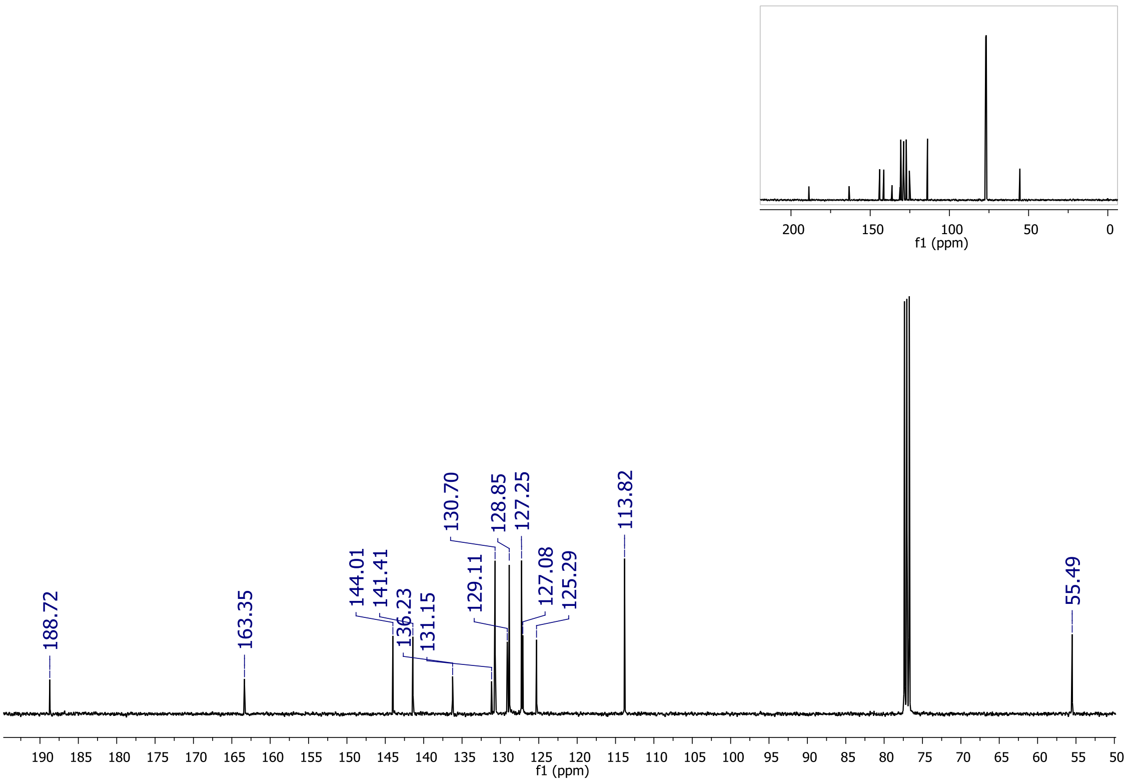

Figure S6. iii) UV-Vis spectrum of cinnamylideneacetophenone **7**, MeOH/H_2O (3:1)

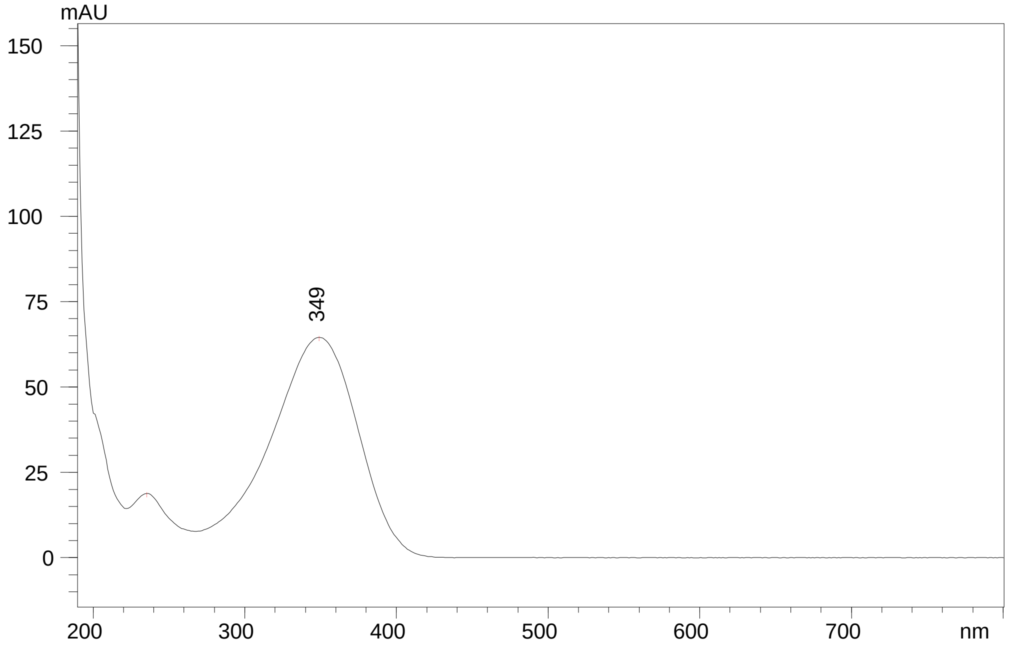

Figure S6. iv) HPLC chromatogram of cinnamylideneacetophenone **7**

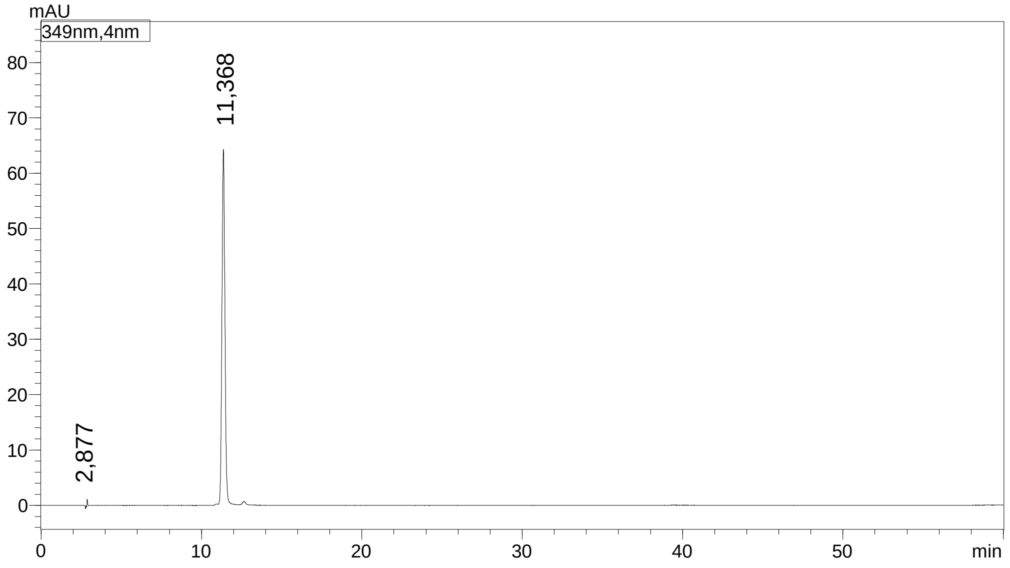

Figure S7. i) ^{1}H NMR spectrum of cinnamylideneacetophenone **8**

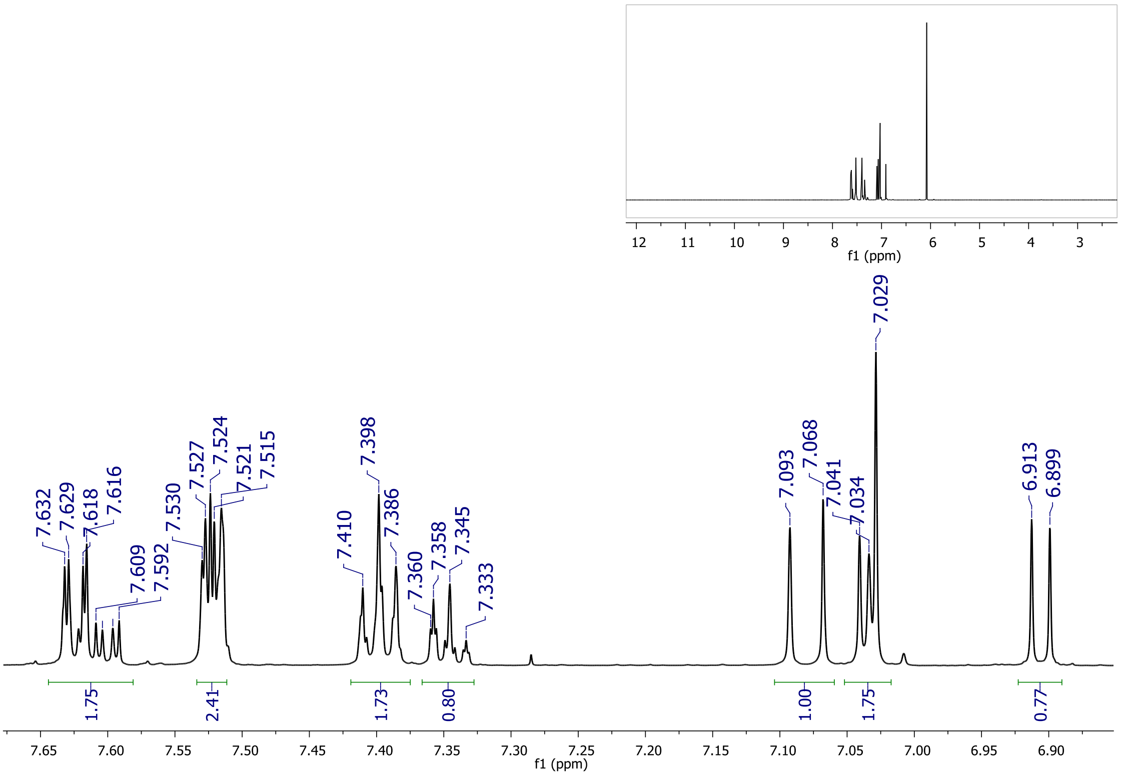

Figure S7. ii) ^{13}C-NMR spectrum of cinnamylideneacetophenone **8**

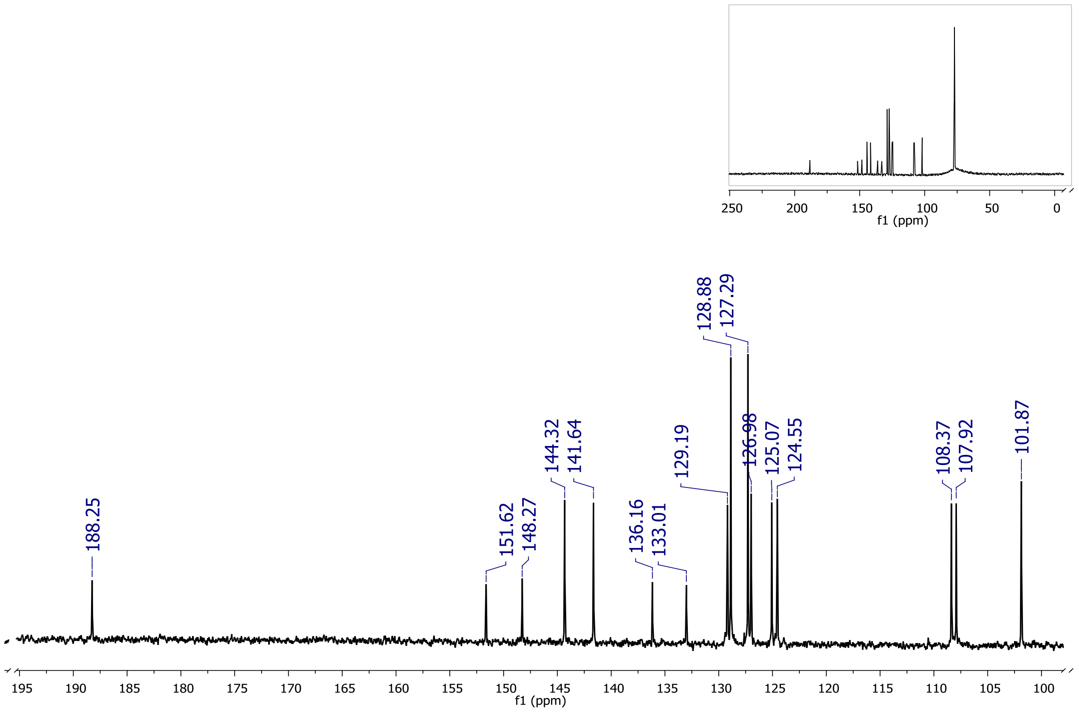

Figure S7. iii) UV-Vis spectrum of cinnamylideneacetophenone **8**, MeOH/H_2O (3:1)

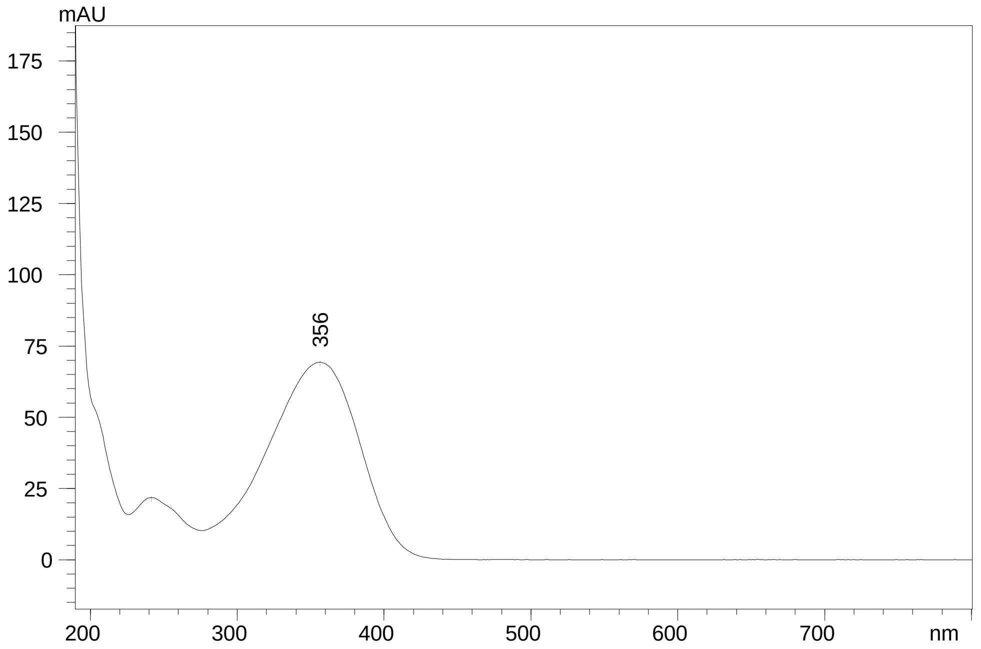

Figure S7. iv) HPLC chromatogram of cinnamylideneacetophenone **8**

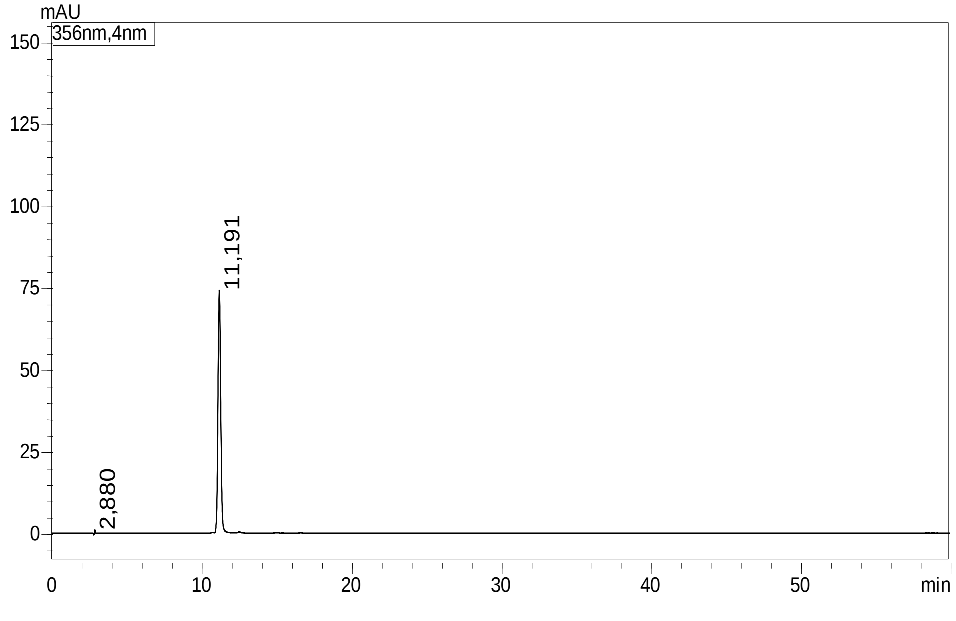

Figure S8. i) ^{1}H NMR spectrum of cinnamylideneacetophenone **9**

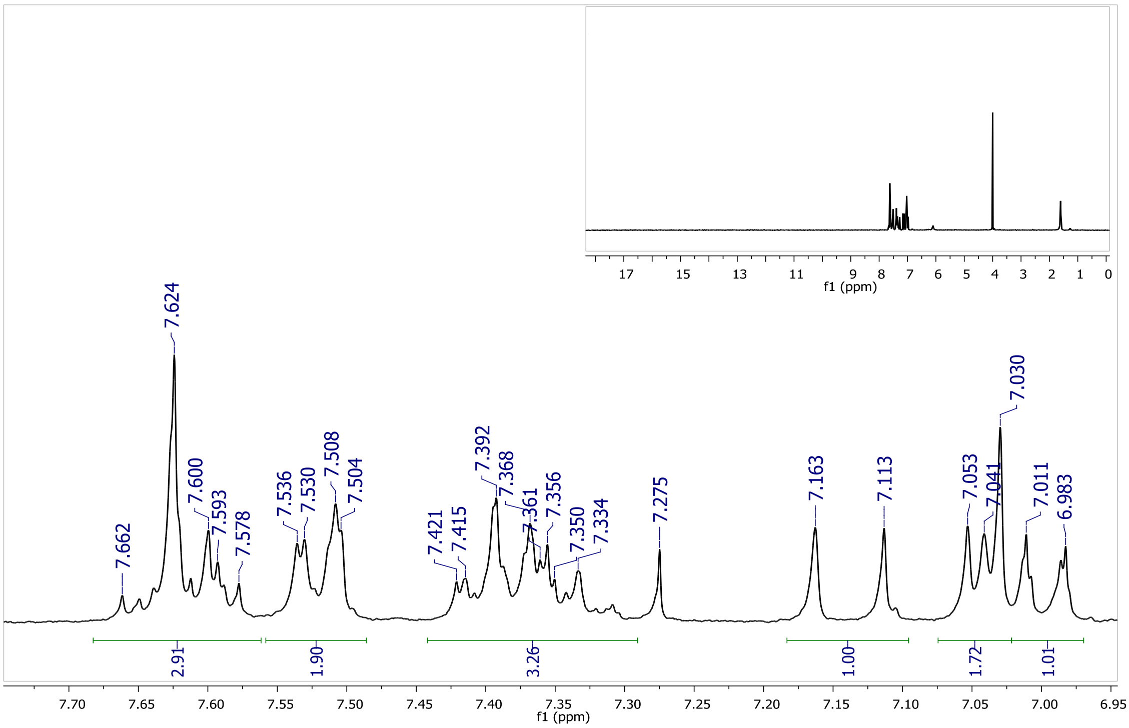

Figure S8. ii) ^{13}C NMR spectrum of cinnamylideneacetophenone **9**

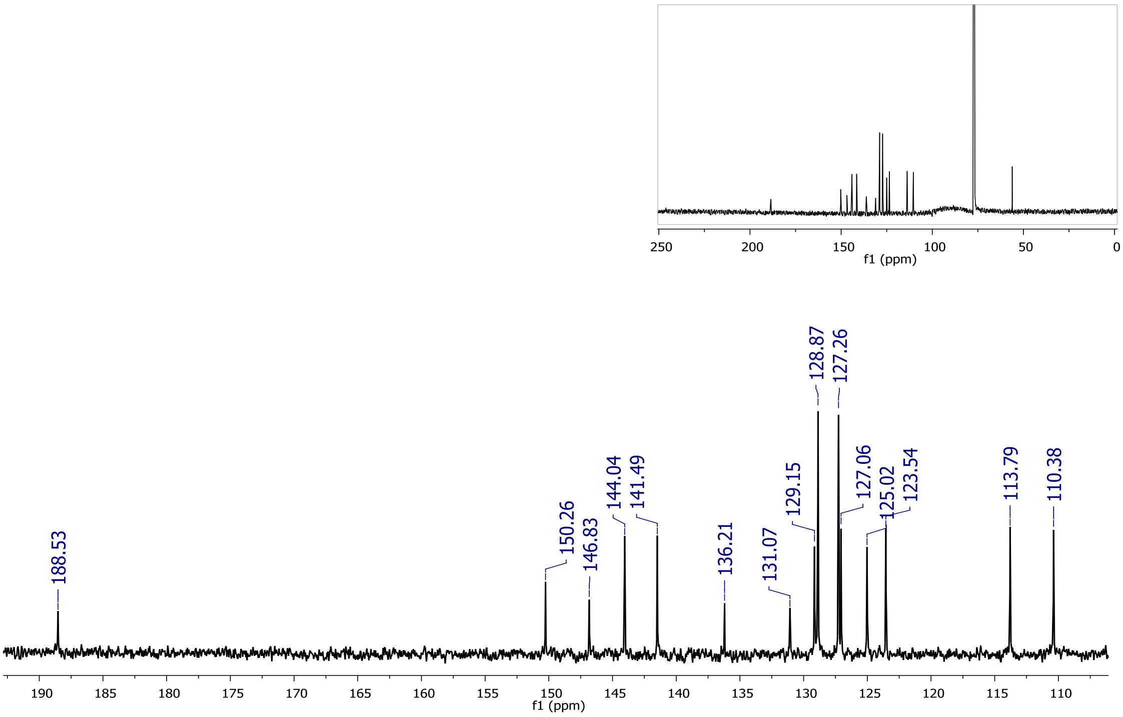

Figure S8. iii) UV-Vis spectrum of cinnamylideneacetophenone **9**, MeOH/H_2O (3:1)

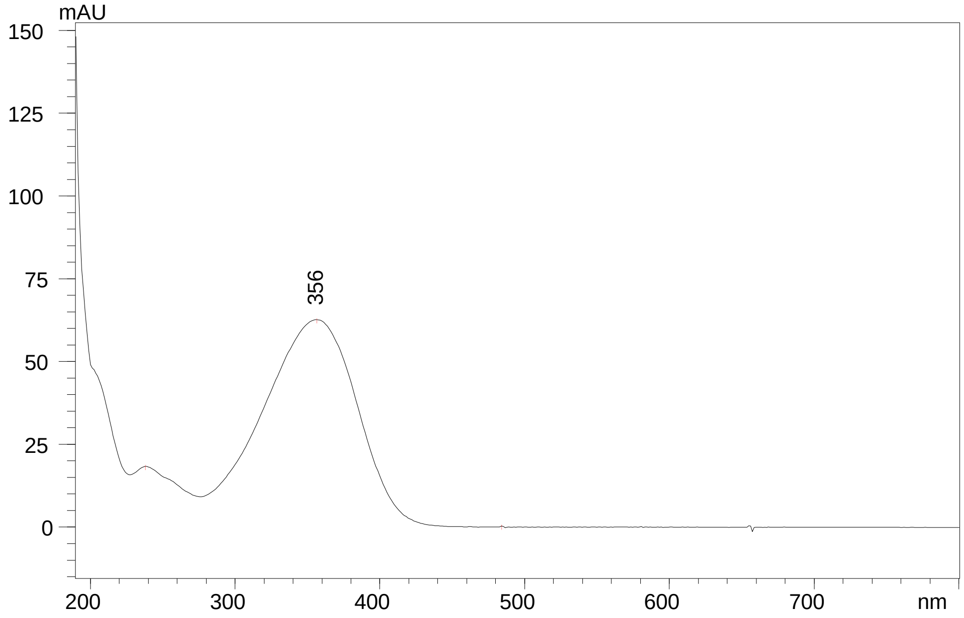

Figure S8. iv) HPLC chromatogram of cinnamylideneacetophenone **9**

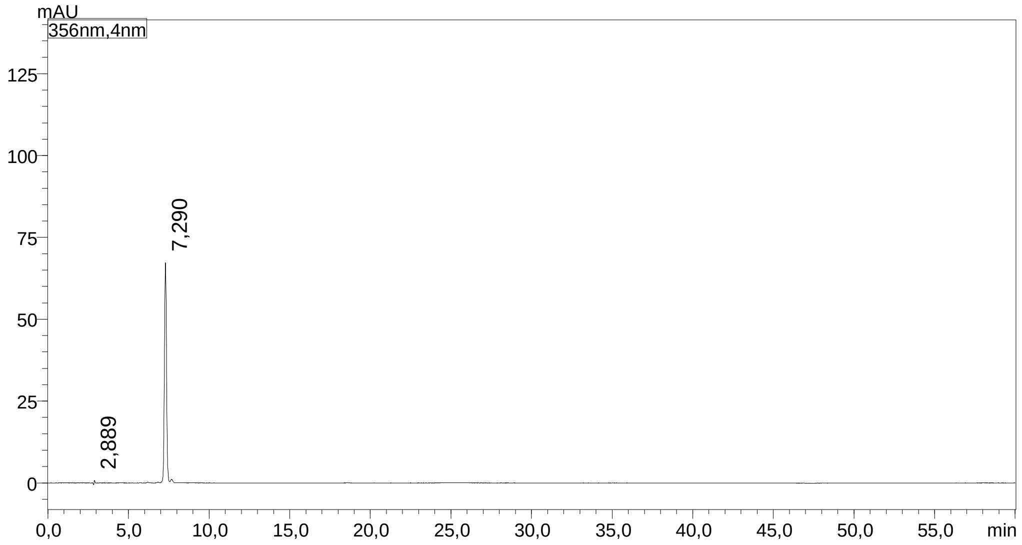

Figure S9. i) ^{1}H NMR spectrum of cinnamylideneacetophenone **10**

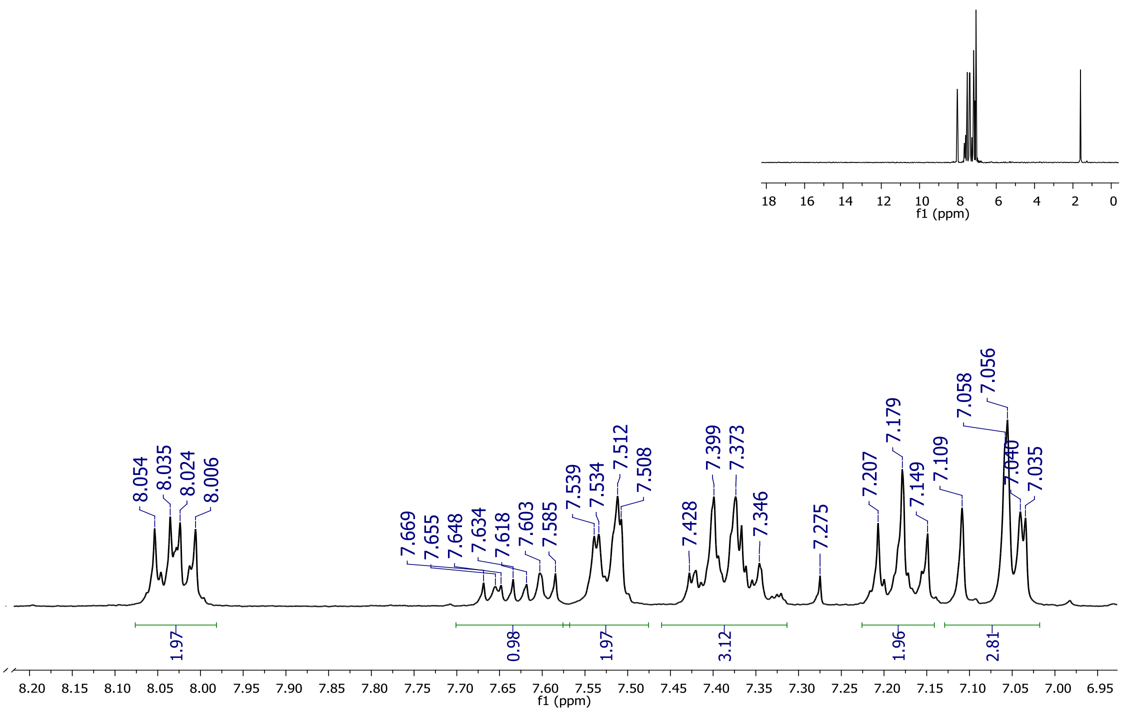

Figure S9. ii) ^{13}C NMR spectrum of cinnamylideneacetophenone **10**

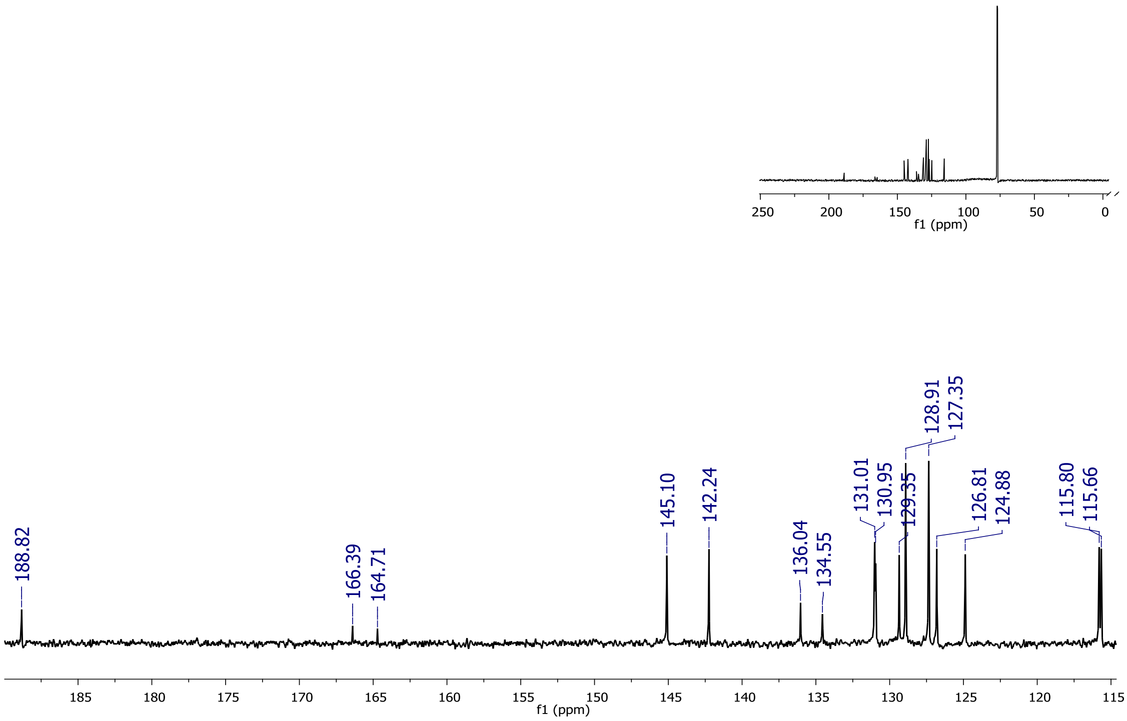

Figure S9. iii) UV-Vis spectrum of cinnamylideneacetophenone **10**, MeOH/H_2O (3:1)

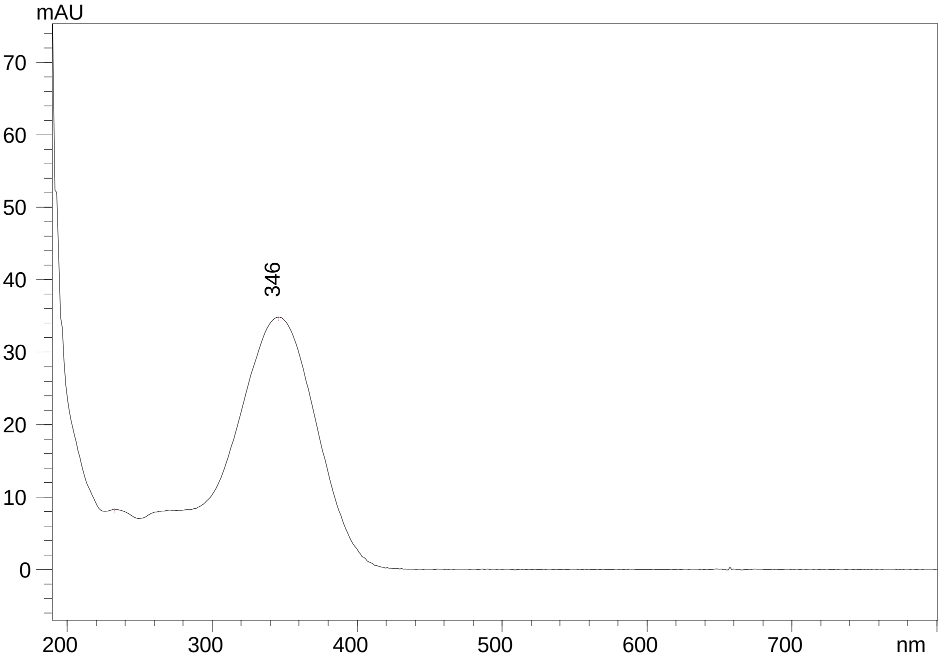

Figure S9. iv) HPLC chromatogram of cinnamylideneacetophenone **10**

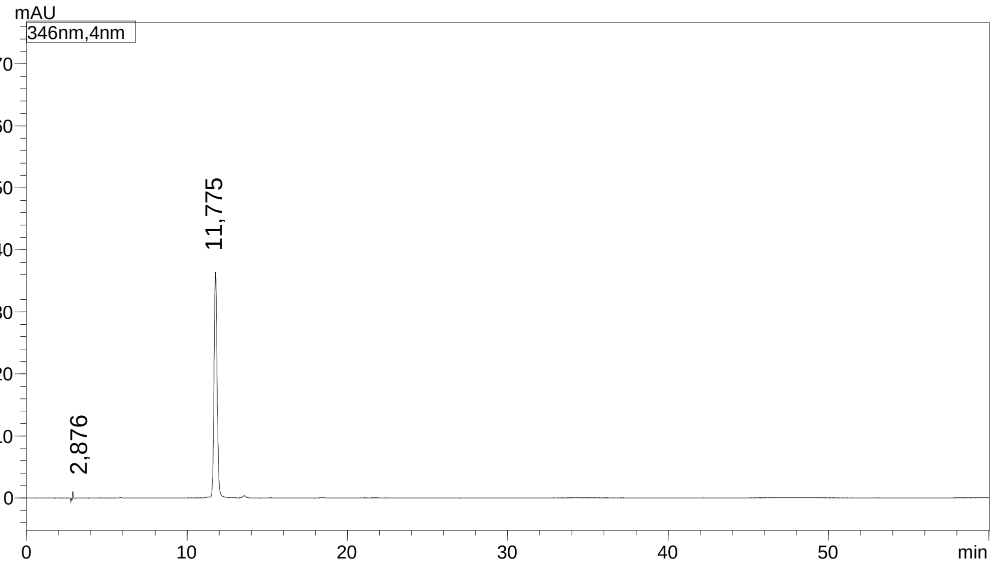

Figure S10. i) ^{1}H NMR spectrum of cinnamylideneacetophenone **11**

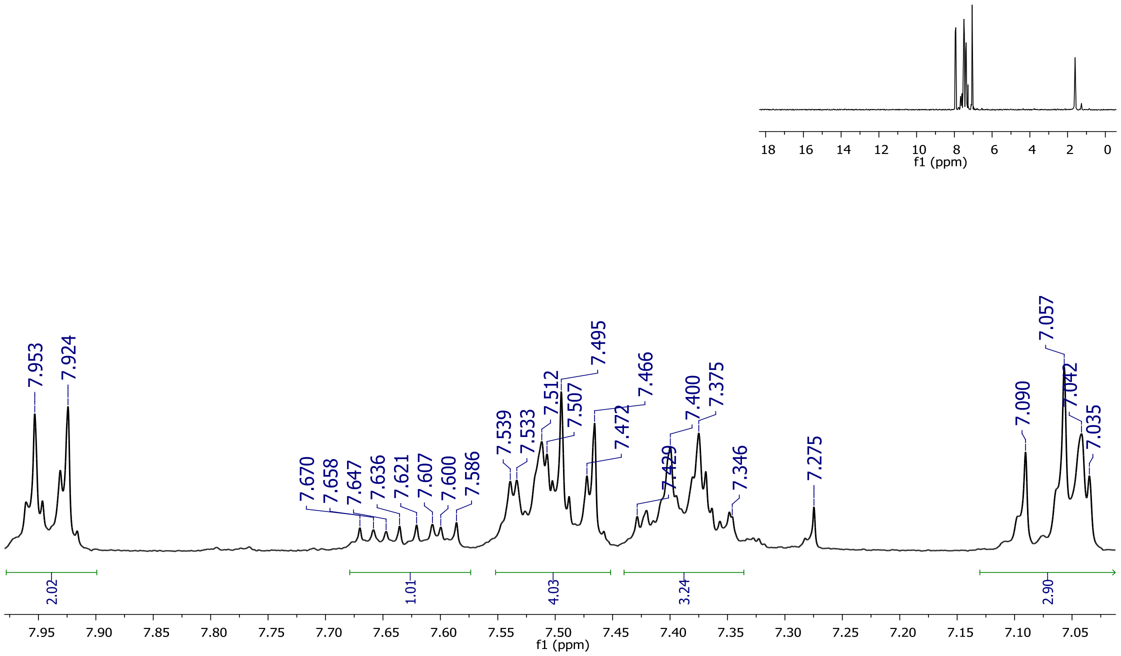

Figure S10. ii) ^{13}C NMR spectrum of cinnamylideneacetophenone **11**

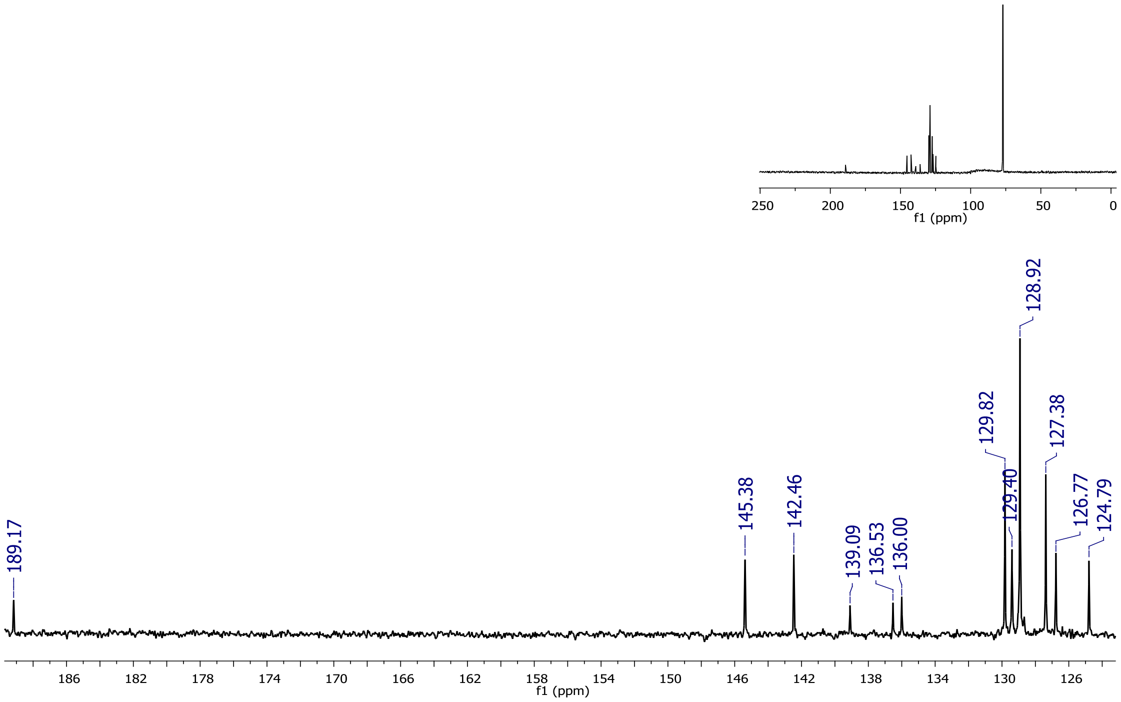

Figure S10. iii) UV-Vis spectrum of cinnamylideneacetophenone **11**, MeOH/H_2O (3:1)

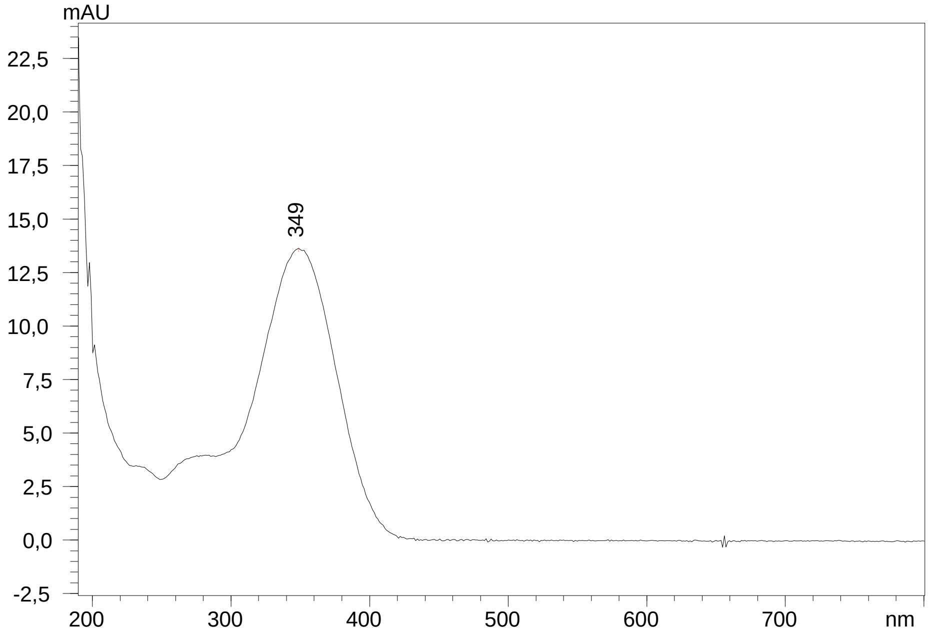

Figure S10. iv) HPLC chromatogram of cinnamylideneacetophenone **11**

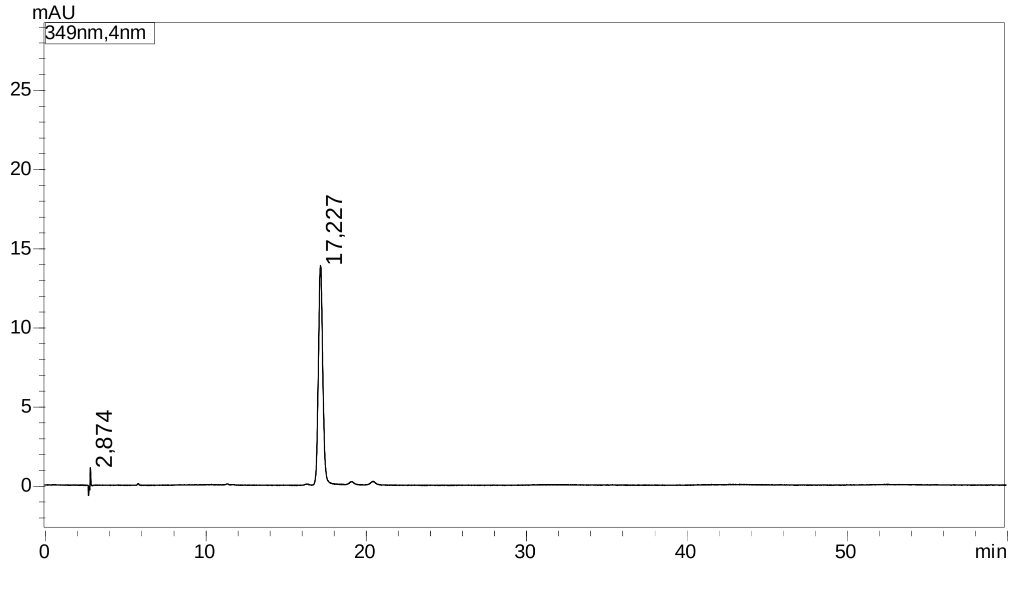

Figure S11. i) ^{1}H NMR spectrum of cinnamylideneacetophenone **12**

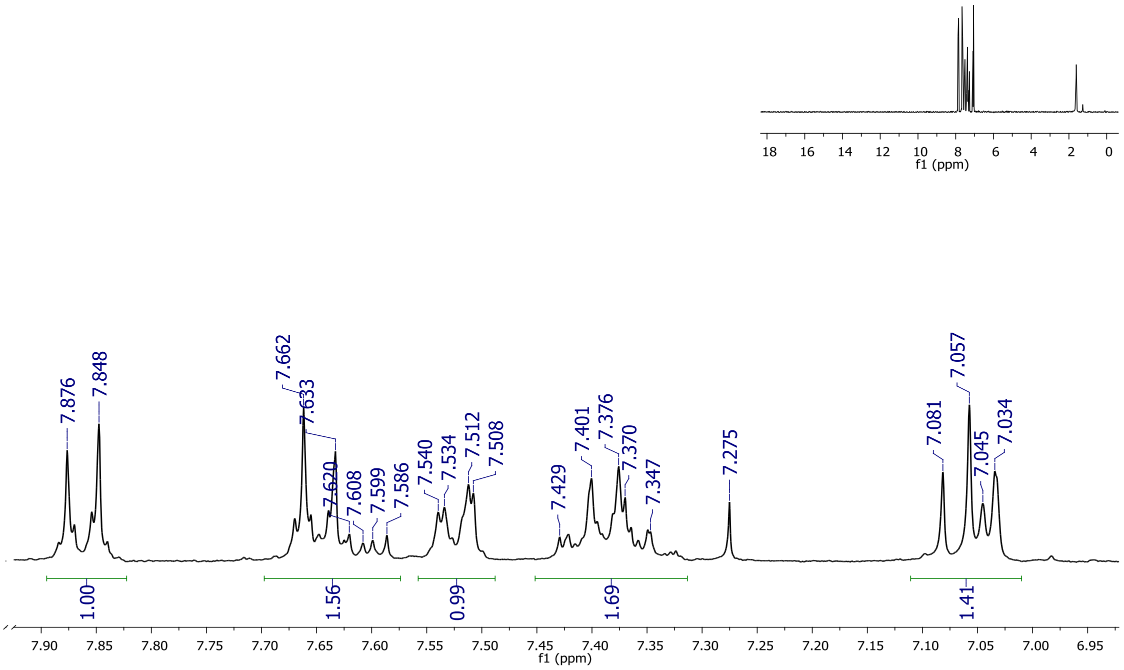

Figure S11. ii) ^{13}C NMR spectrum of cinnamylideneacetophenone **12**

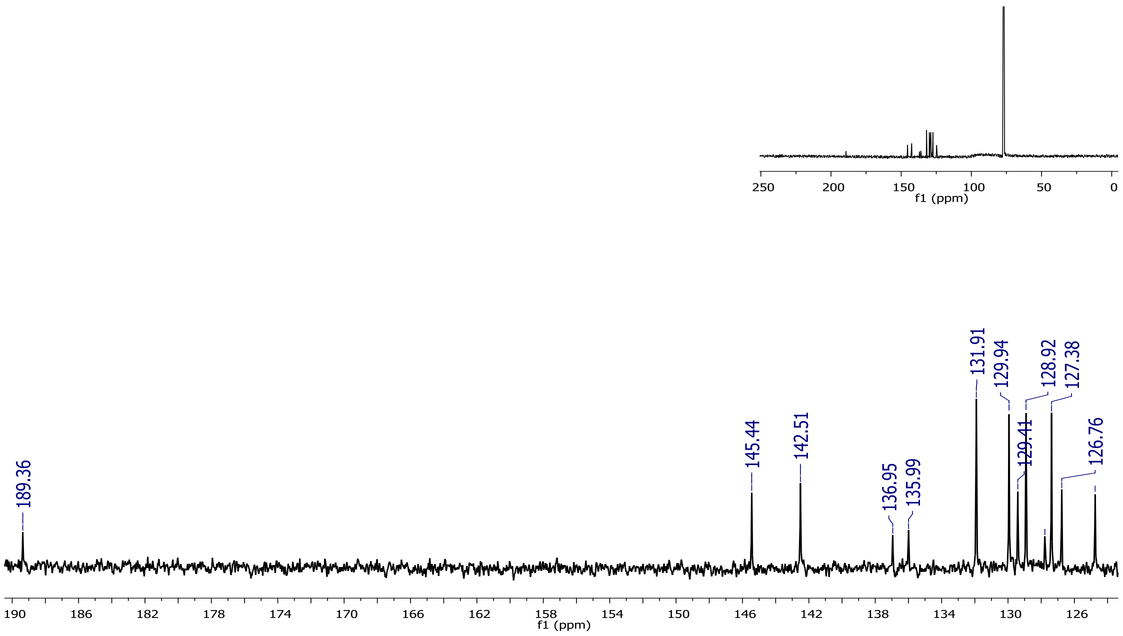

Figure S11. iii) UV-Vis spectrum of cinnamylideneacetophenone **12**, MeOH/H_2O (3:1)

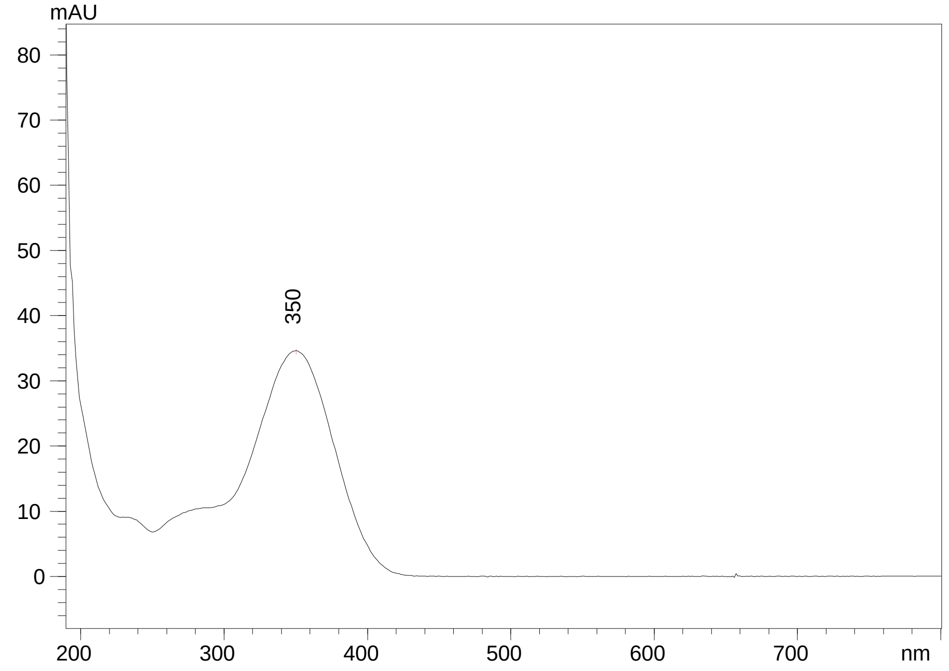

Figure S11. iv) HPLC chromatogram of cinnamylideneacetophenone **12**

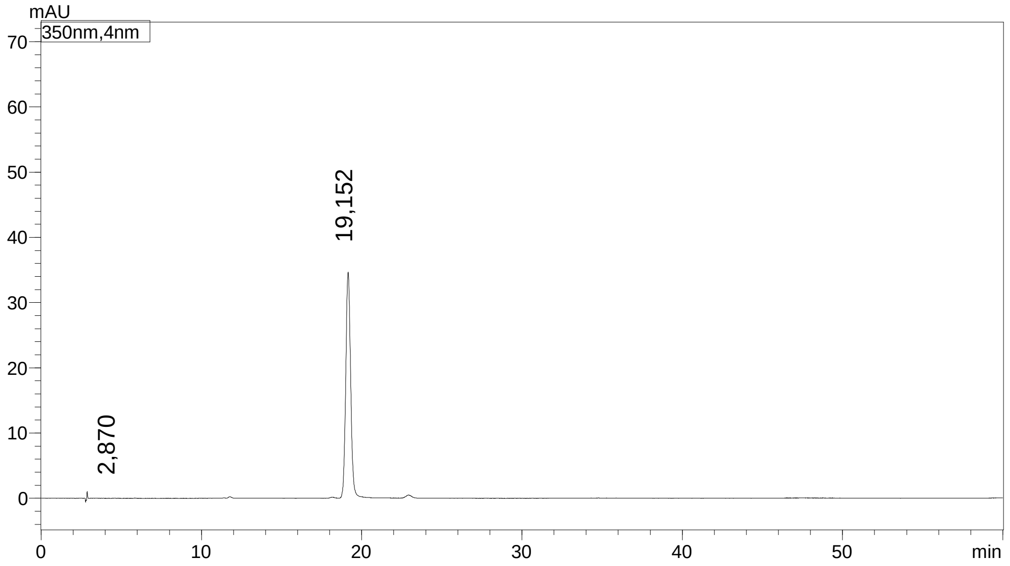

Figure S12. i) ^{1}H NMR spectrum of cinnamylideneacetophenone **13**

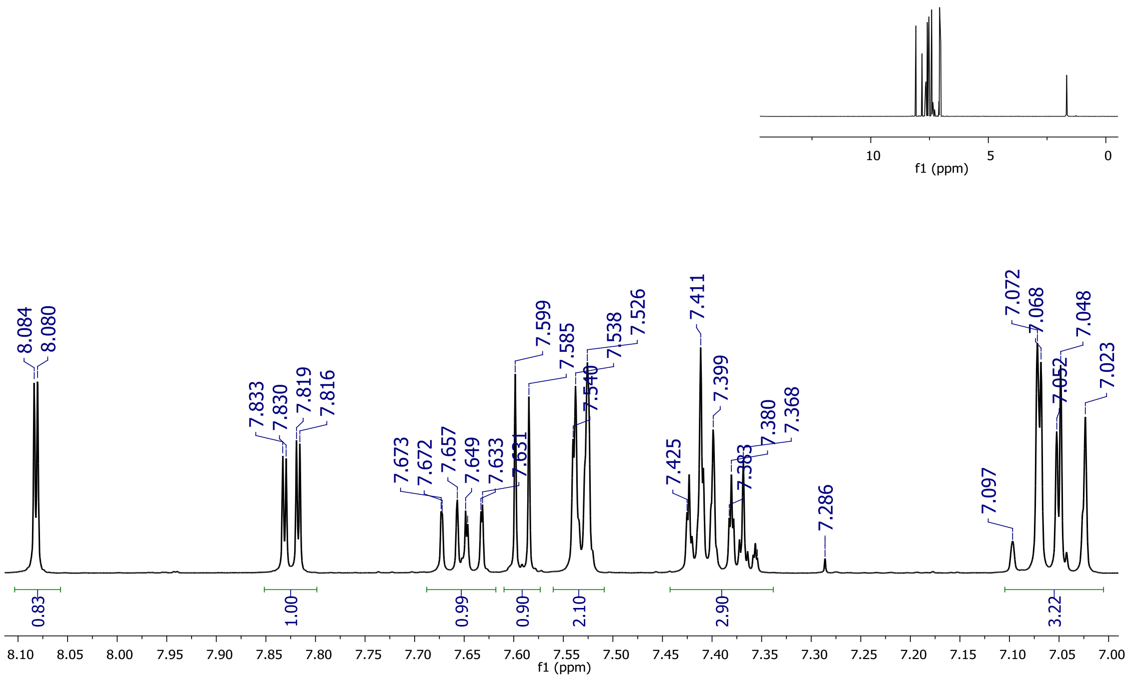

Figure S12. ii) ^{13}C NMR spectrum of cinnamylideneacetophenone **13**

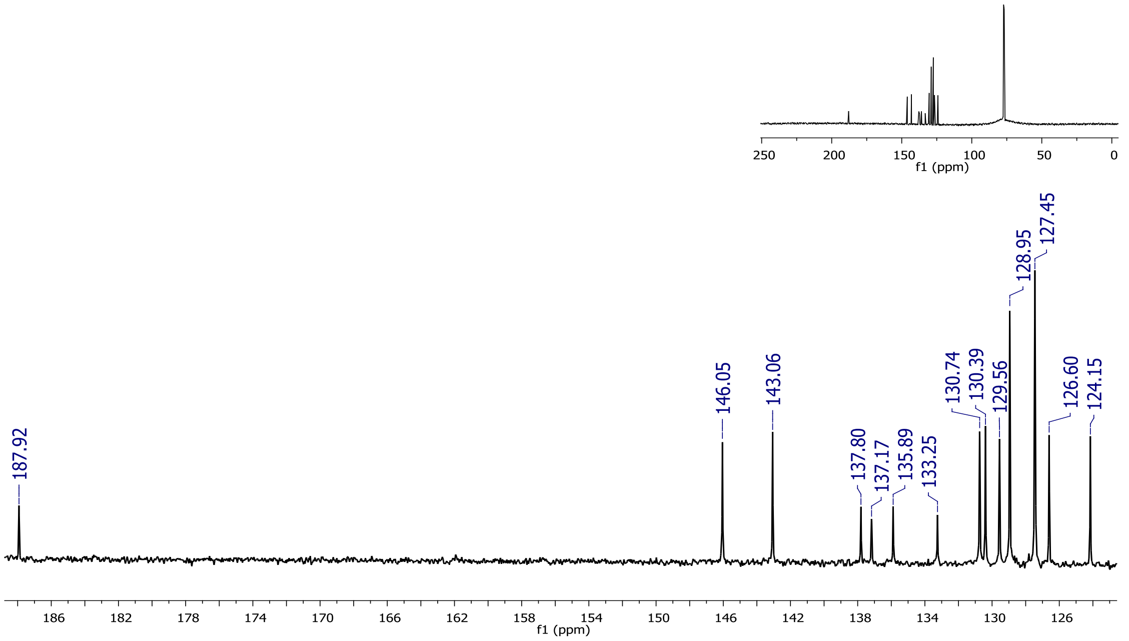

Figure S12. iii) UV-Vis spectrum of cinnamylideneacetophenone **13**, MeOH/H_2O (3:1)

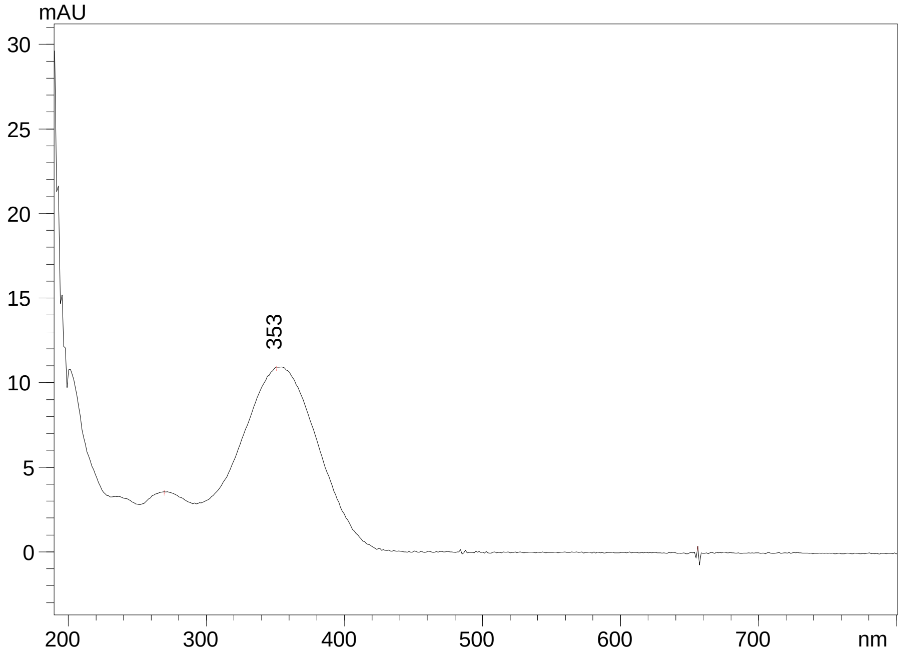

Figure S12. iv) HPLC chromatogram of cinnamylideneacetophenone **13**

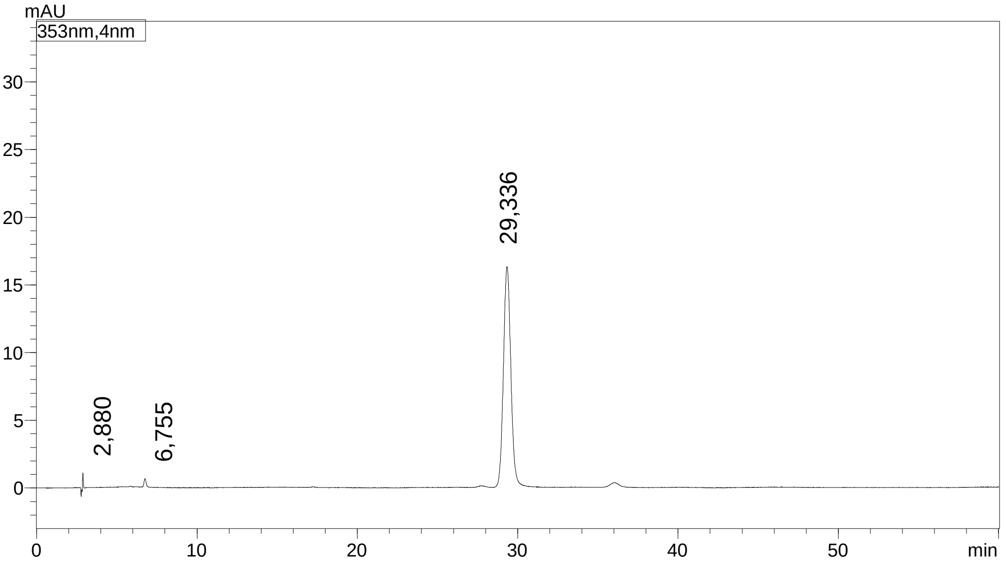

Figure S13. i) ^{1}H NMR spectrum of cinnamylideneacetophenone **14**

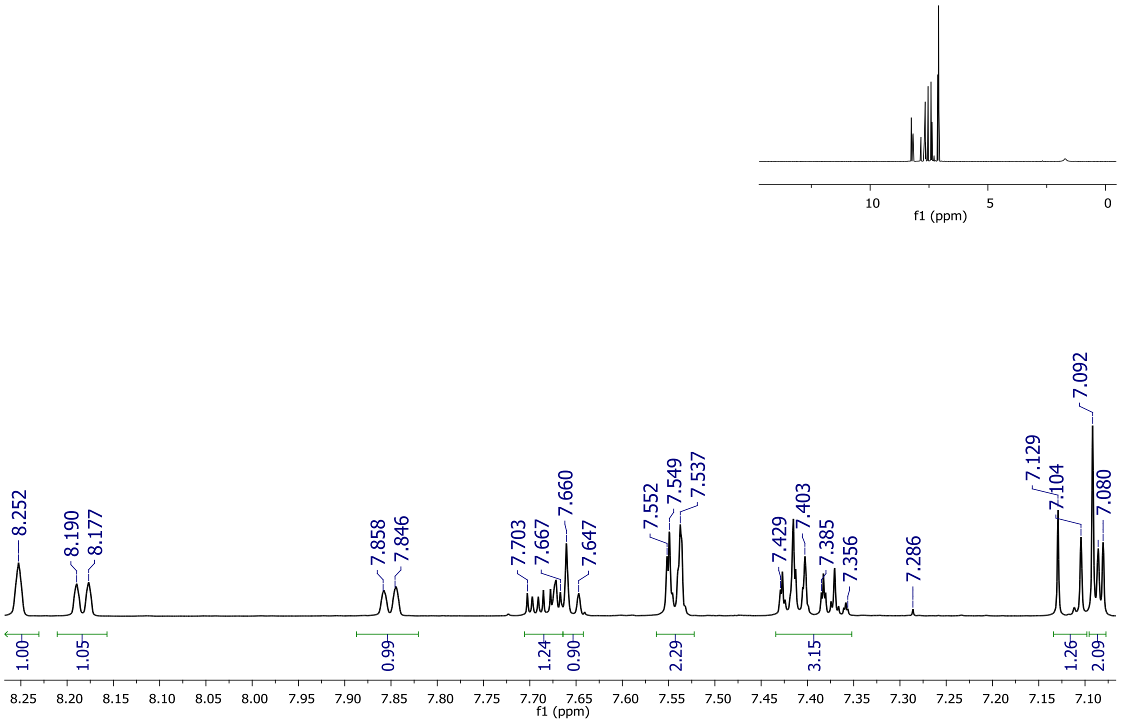

Figure S13. ii) ^{13}C NMR spectrum of cinnamylideneacetophenone **14**

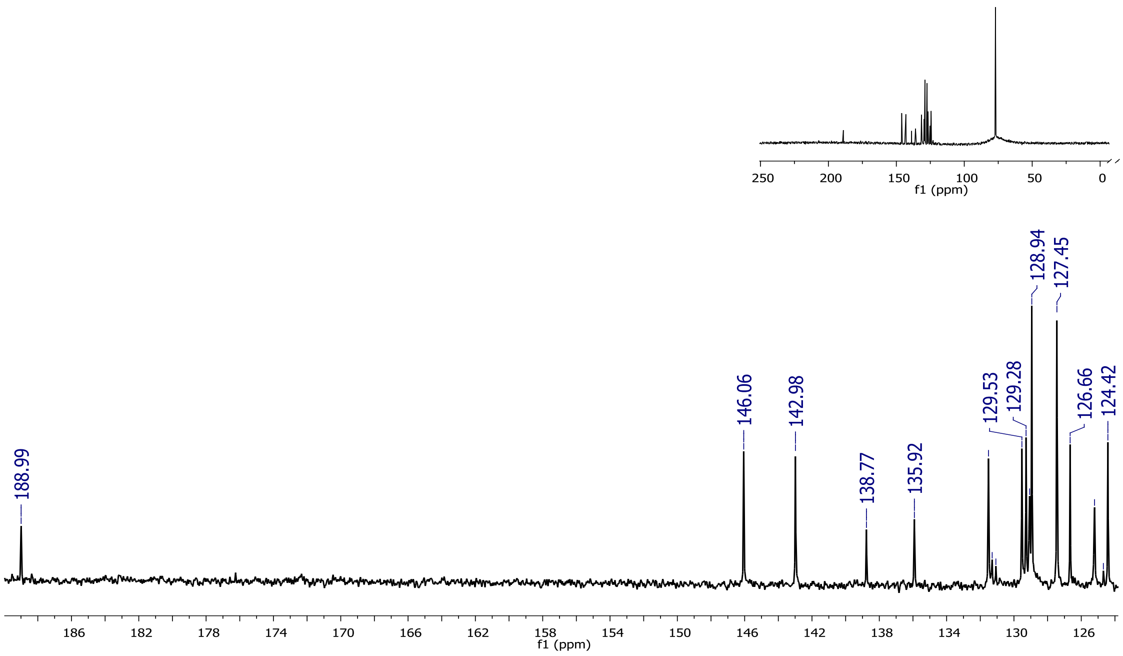

Figure S13. iii) UV-Vis spectrum of cinnamylideneacetophenone **14**, MeOH/H_2O (3:1)

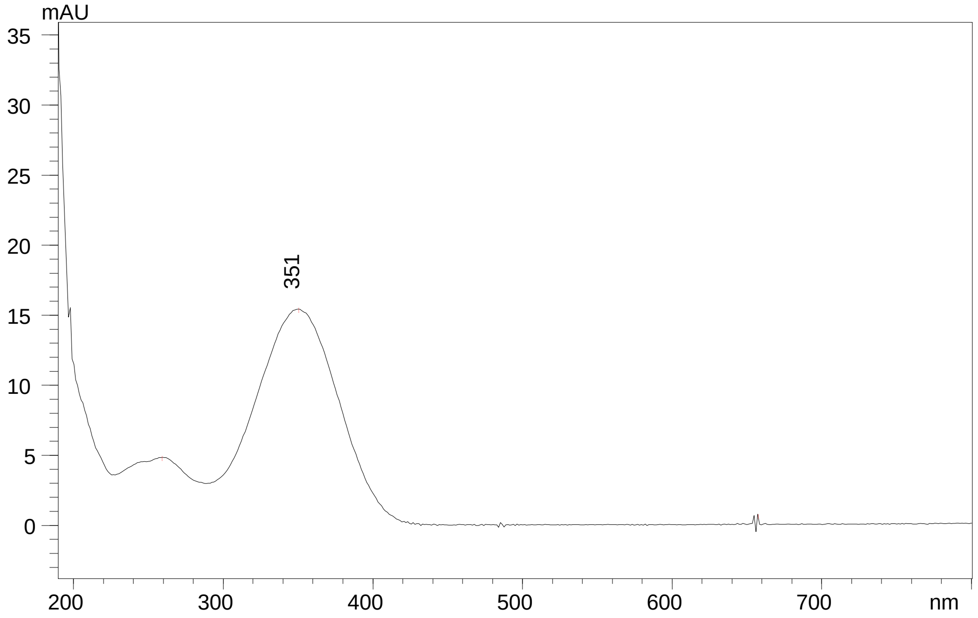

Figure S13. iv) HPLC chromatogram of cinnamylideneacetophenone **14**

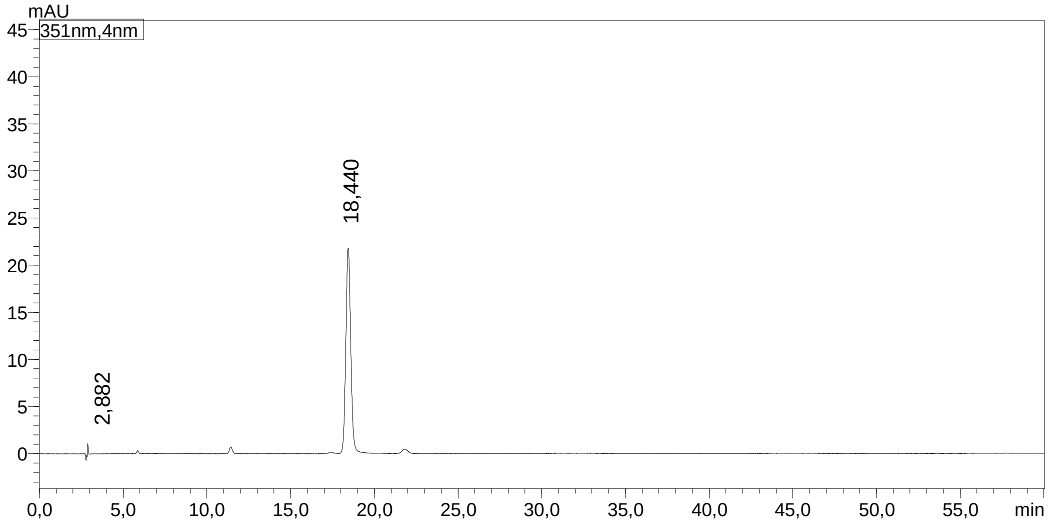

Figure S14. i) ^{1}H NMR spectrum of cinnamylideneacetophenone **15**

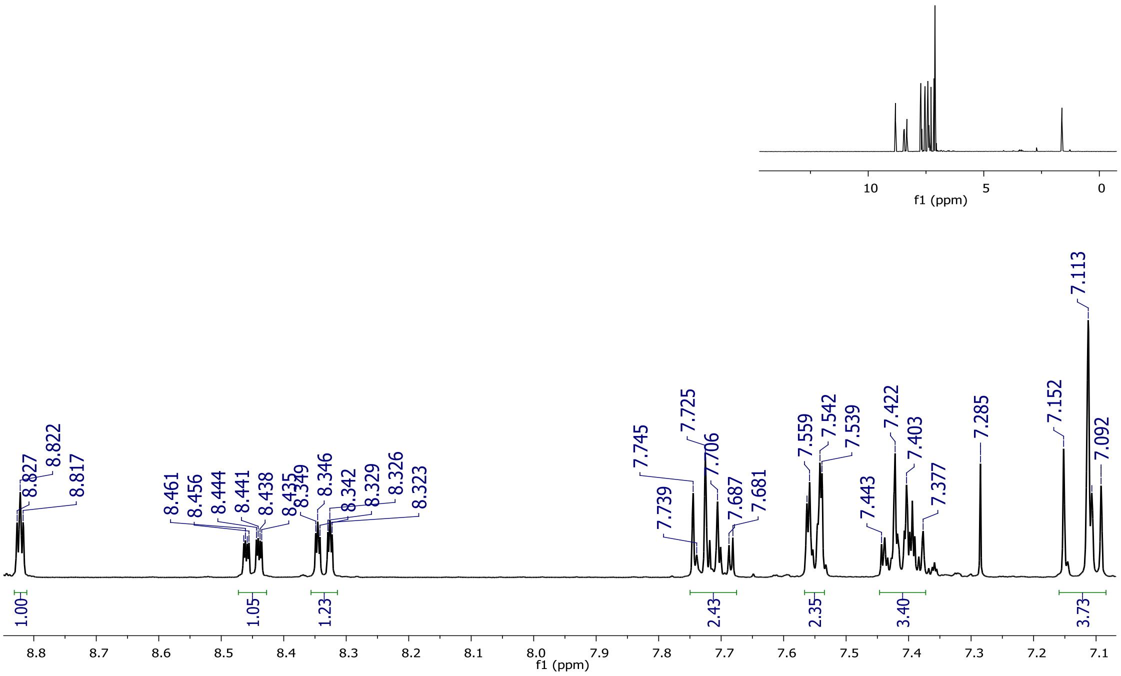

Figure S14. ii) ^{13}C NMR spectrum of cinnamylideneacetophenone **15**

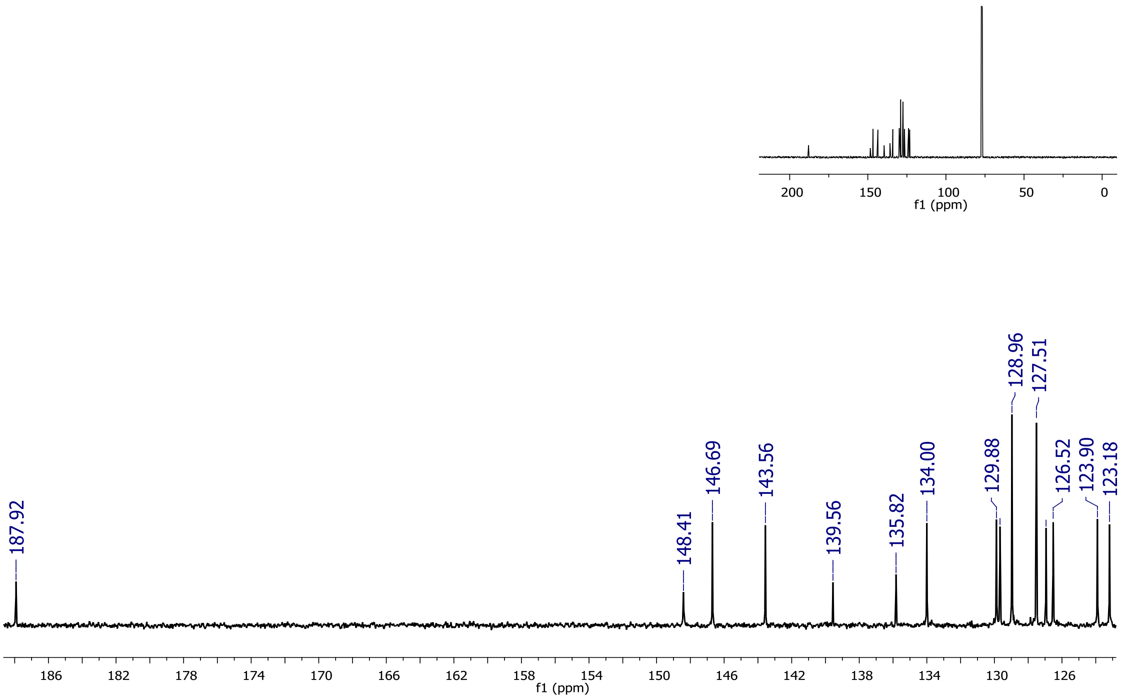

Figure S14. iii) UV-Vis spectrum of cinnamylideneacetophenone **15**, $MeOH/H_2O$ (3:1)

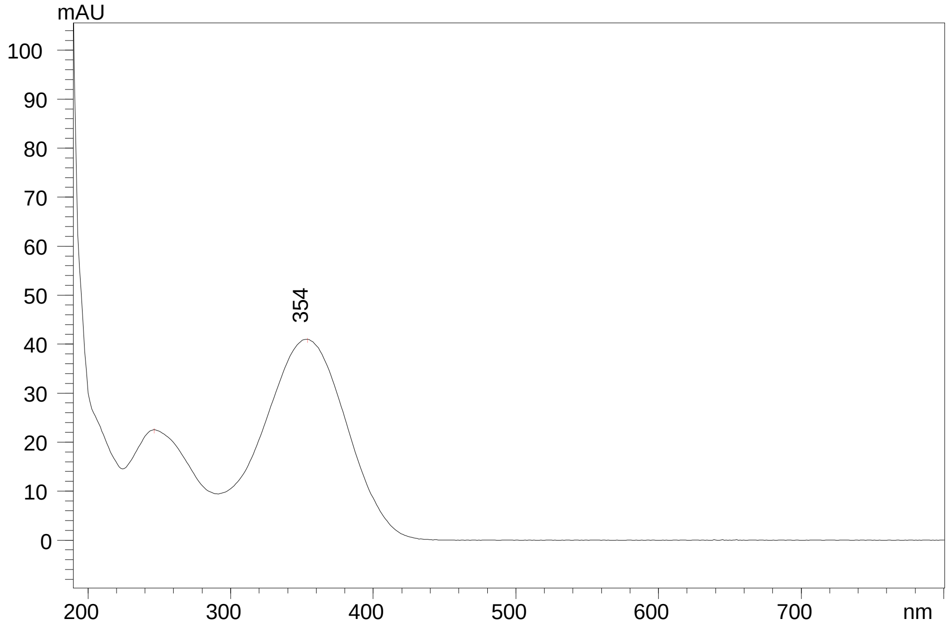

Figure S14. iv) HPLC chromatogram of cinnamylideneacetophenone **15**

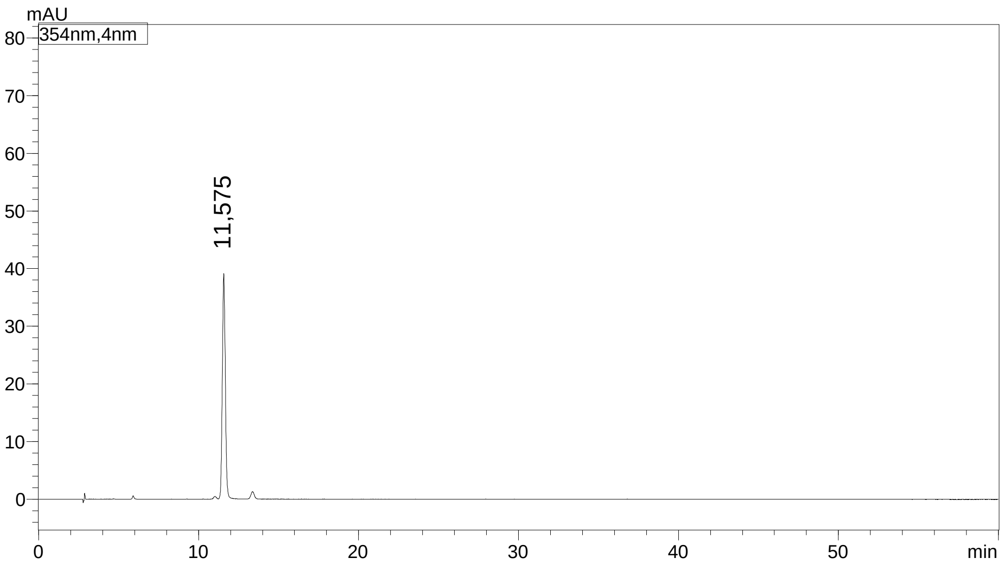

Figure S15. i) ^{1}H NMR spectrum of cinnamylideneacetophenone **16**

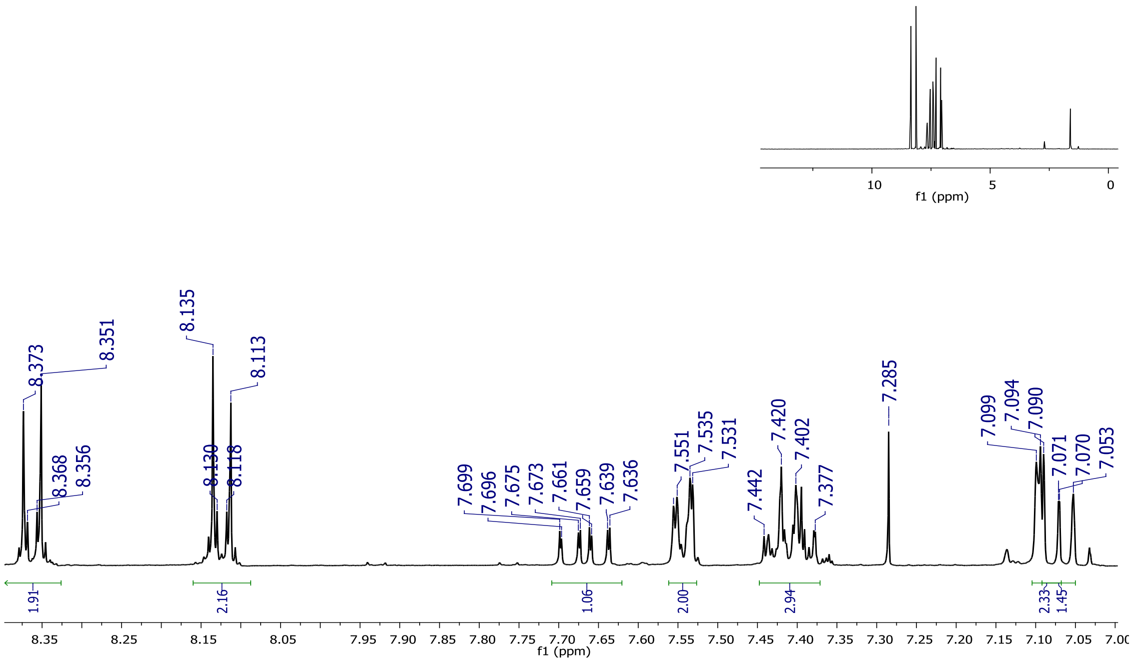

Figure S15. ii) ^{13}C NMR spectrum of cinnamylideneacetophenone **16**

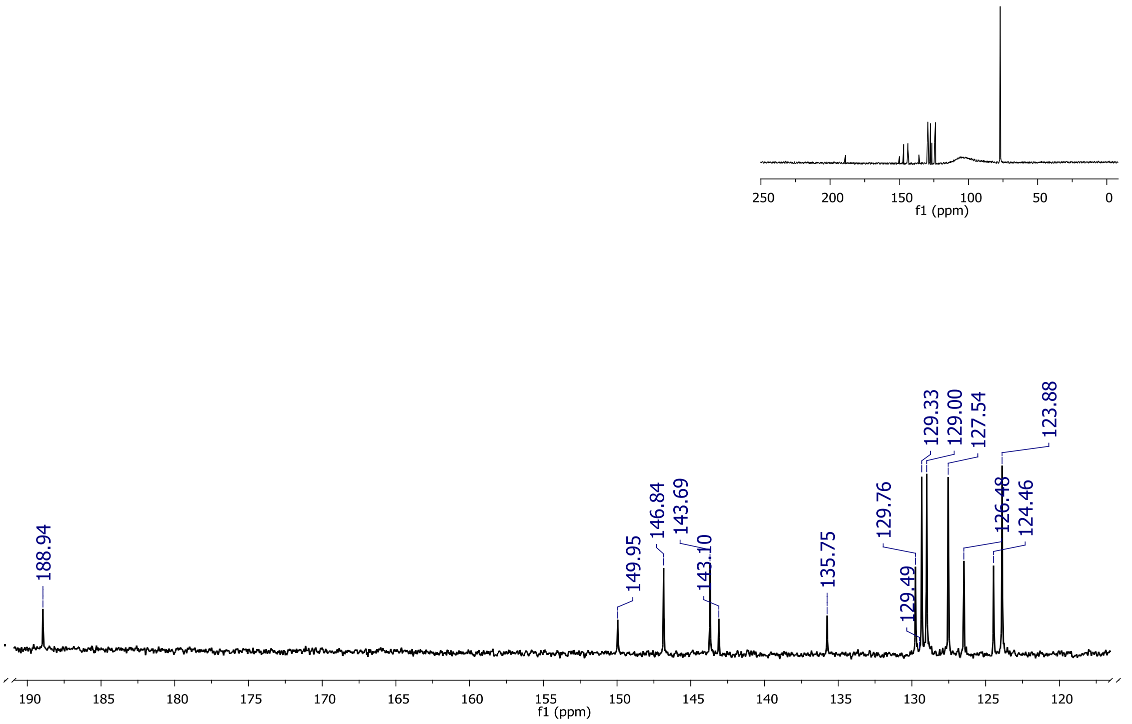

Figure S15. iii) UV-Vis spectrum of cinnamylideneacetophenone **16**, MeOH/H_2O (3:1)

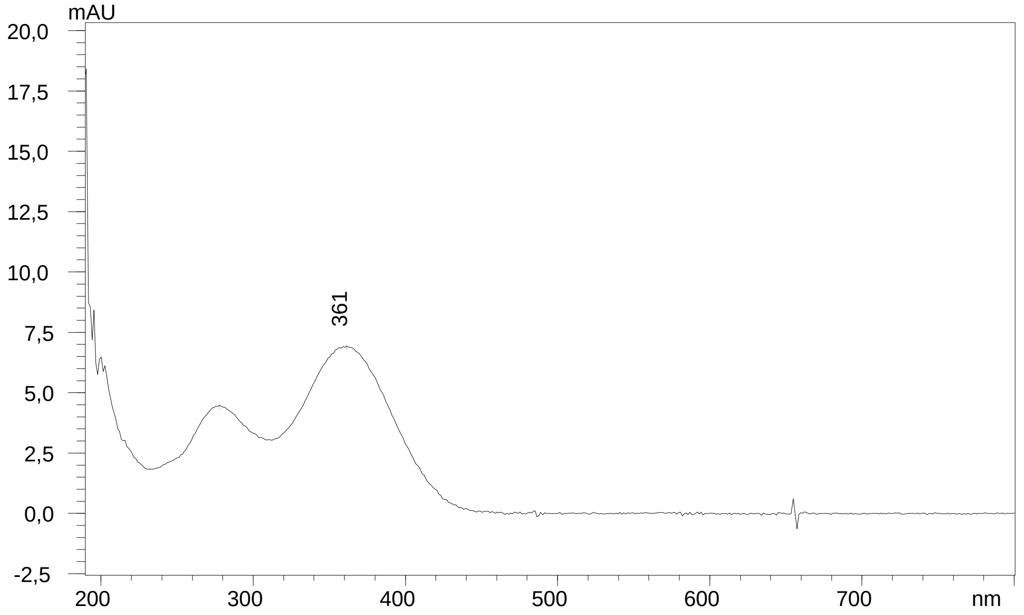

Figure S15. iv) HPLC chromatogram of cinnamylideneacetophenone **16**

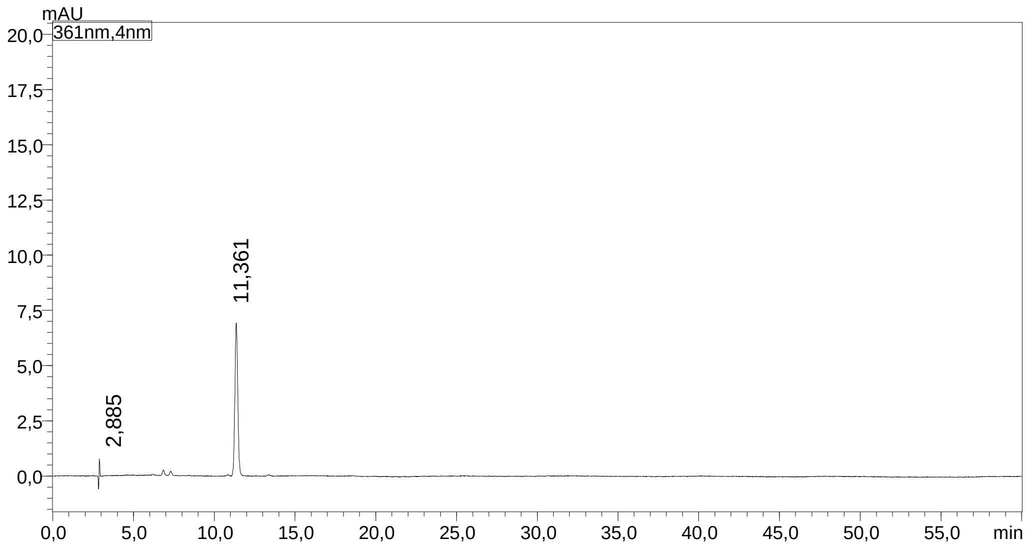

Figure S16. i) ^{1}H NMR spectrum of cinnamylideneacetophenone **17**

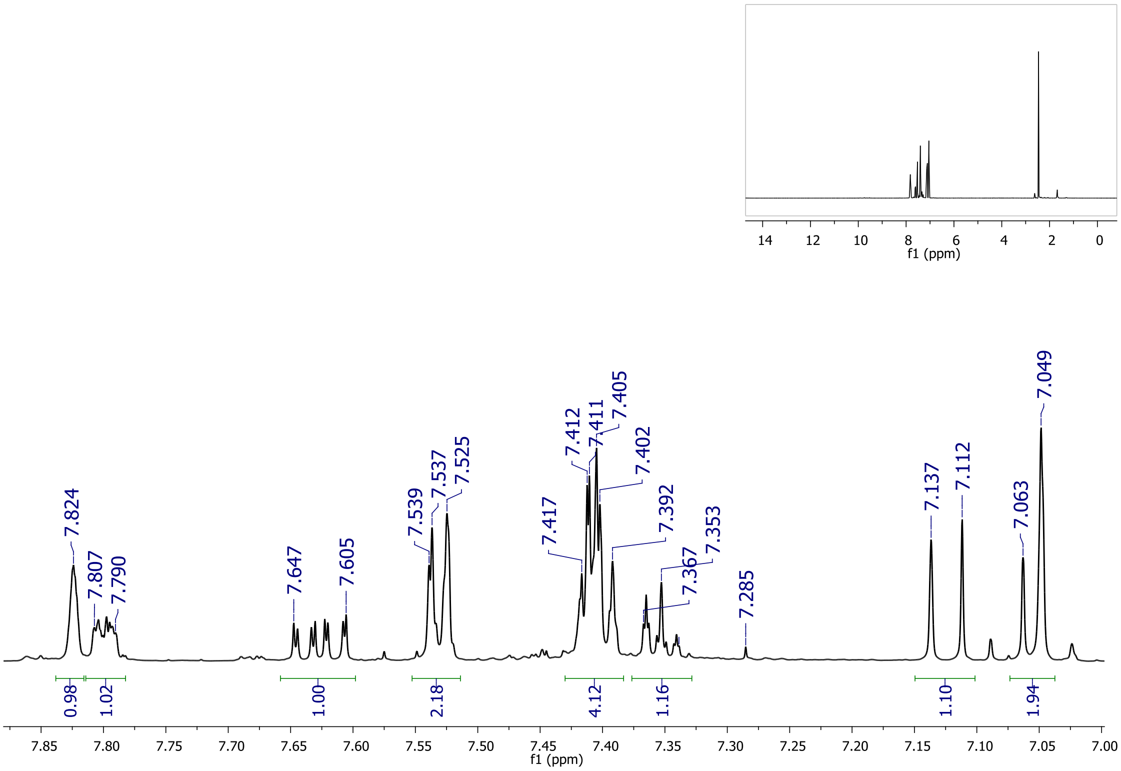

Figure S16. ii) ^{13}C NMR spectrum of cinnamylideneacetophenone **17**

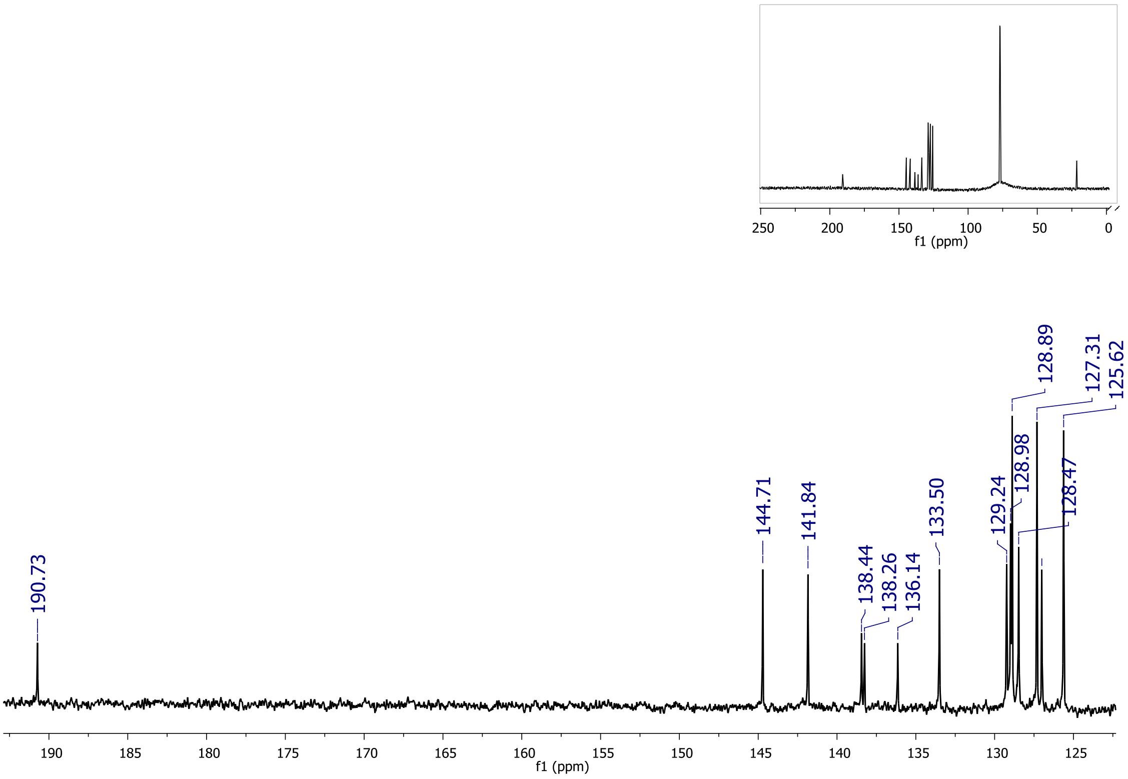

Figure S16. iii) UV-Vis spectrum of cinnamylideneacetophenone **17**, MeOH/H_2O (3:1)

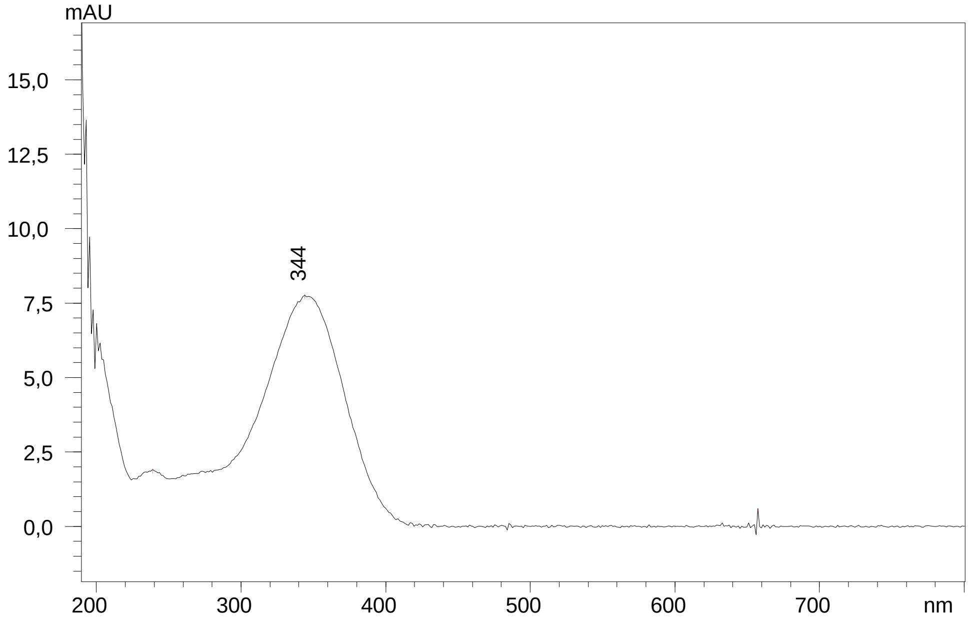

Figure S16. iv) HPLC chromatogram of cinnamylideneacetophenone **17**

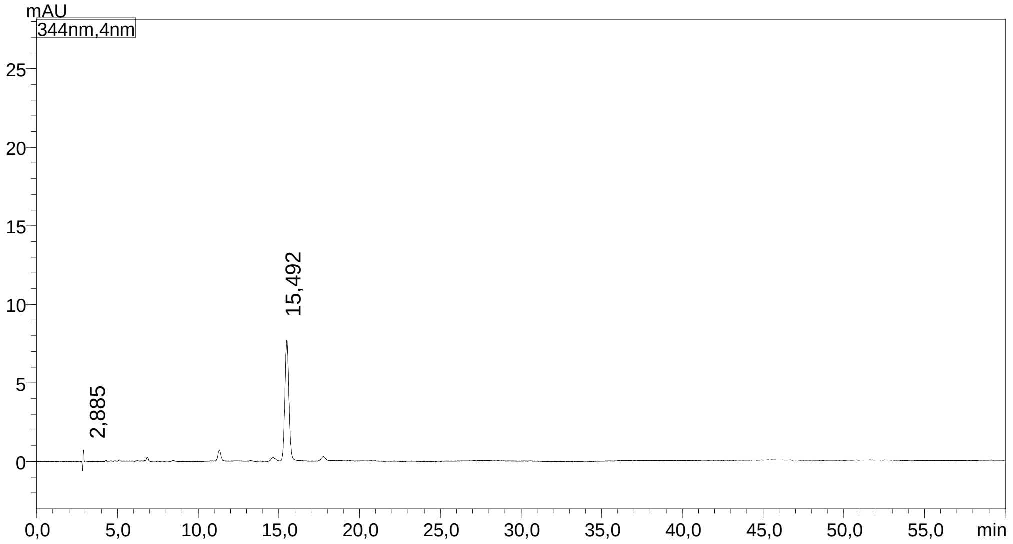

Figure S17. i) ^{1}H NMR spectrum of cinnamylideneacetophenone **18**

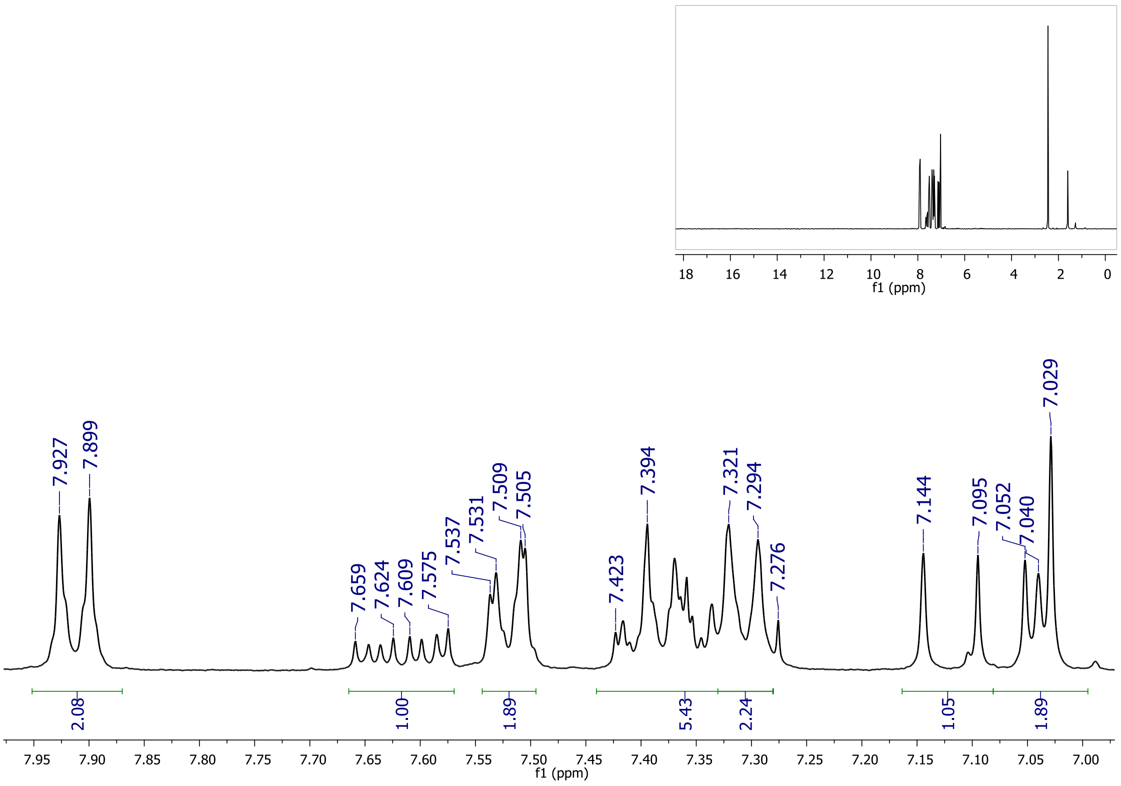

Figure S17. ii) ^{13}C NMR spectrum of cinnamylideneacetophenone **18**

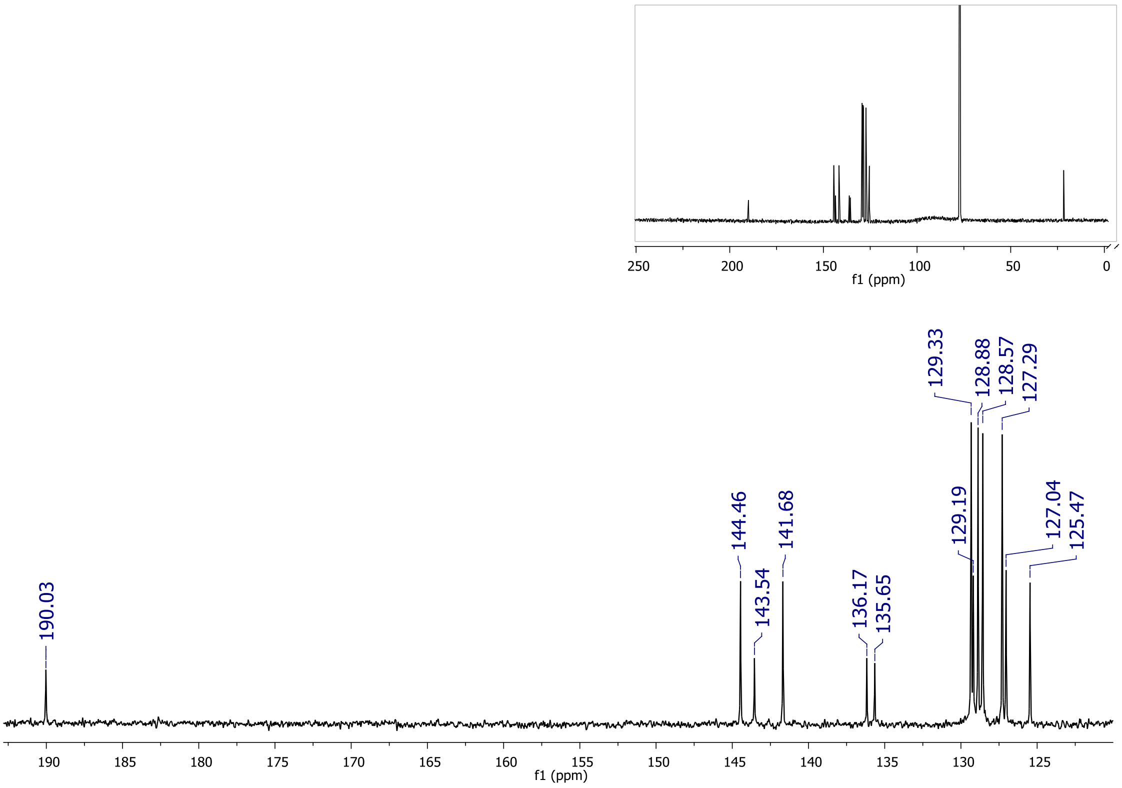

Figure S17. iii) UV-Vis spectrum of cinnamylideneacetophenone **18**, MeOH/H_2O (3:1)

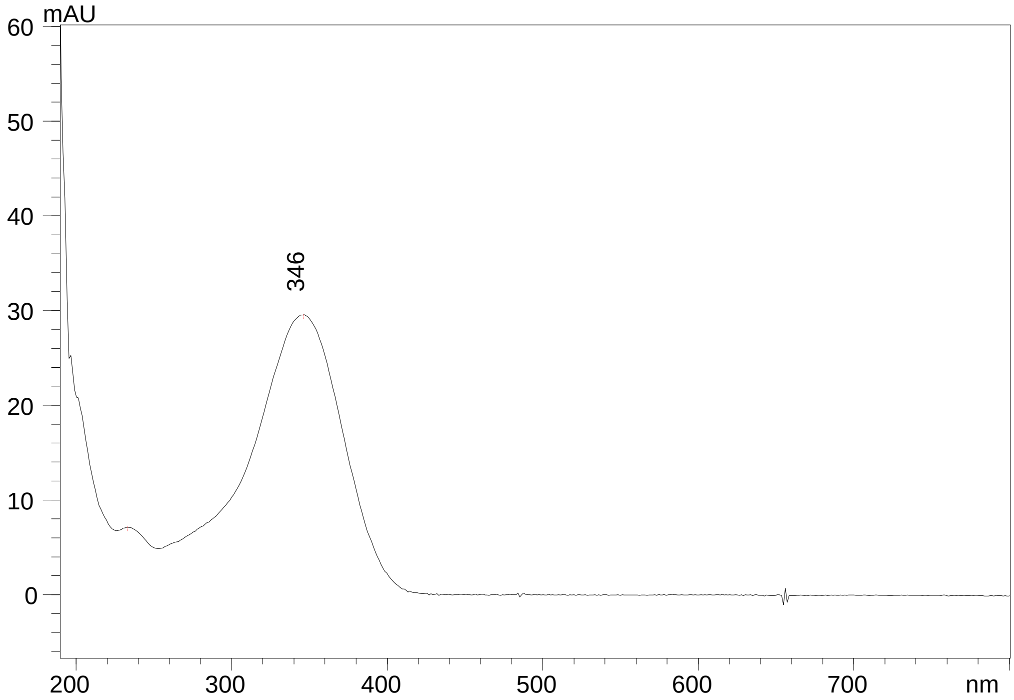

Figure S17. iv) HPLC chromatogram of cinnamylideneacetophenone **18**

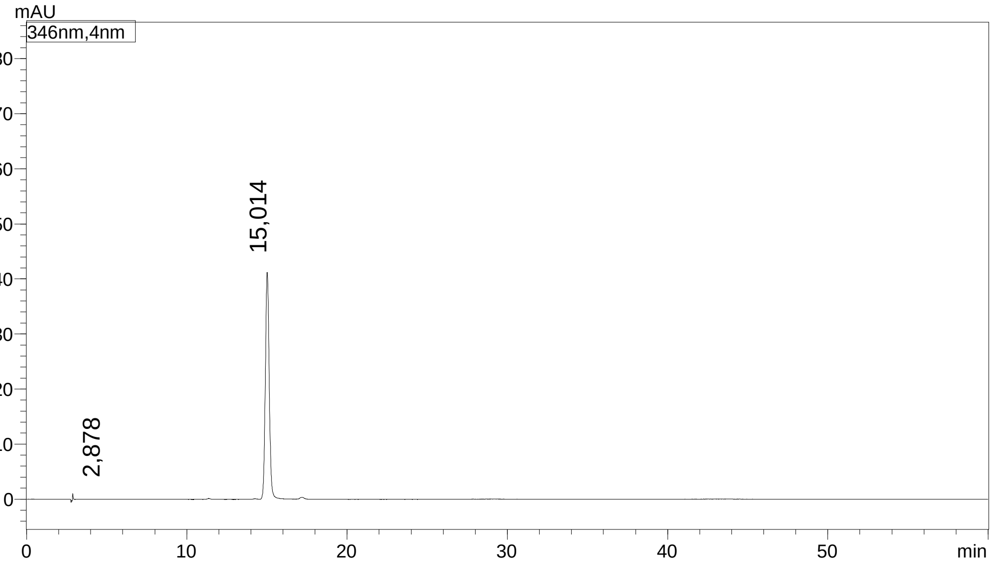

Supplement: Supplementary file 1 [file molecules-22-01685-s001.pdf]
